# Long-Term Outcomes in Nephrotic Syndrome by Kidney Biopsy Diagnosis and Proteinuria

**DOI:** 10.1681/ASN.0000000610

**Published:** 2025-04-17

**Authors:** David Pitcher, Fiona Braddon, Bruce Hendry, Alex Mercer, Jonathan Barratt, Retha Steenkamp, Katie Wong, A. Neil Turner, Wu Gong, Daniel P. Gale, Moin A. Saleem

**Affiliations:** 1 National Registry of Rare Kidney Diseases, UK Kidney Association, Bristol, United Kingdom; 2 Travere Therapeutics, Inc., San Diego, California; 3 JAMCO Pharma Consulting, Stockholm, Sweden; 4 Leicester General Hospital, University of Leicester, Leicester, United Kingdom; 5 University of Edinburgh, Edinburgh, United Kingdom; 6 Department of Renal Medicine, University College London, London, United Kingdom; 7 Bristol Royal Hospital for Children, University of Bristol, Bristol, United Kingdom

**Keywords:** chronic GN, glomerulosclerosis, idiopathic nephrotic syndrome, nephrotic syndrome, pediatric nephrology, primary GN, proteinuria, renal function decline, glomerular diseases

## Abstract

**Key Points:**

- In nephrotic syndrome, diagnosis was associated with kidney failure risk: very high in monogenic cases, substantial in FSGS, and less but not zero in minimal change disease.
- Early control of proteinuria was associated with lower kidney failure risk, and in FSGS levels <1.5 g/g are associated with good kidney outcomes at 10 years.
- Monogenic nephrotic syndrome cases had very high proteinuria and rapid progression to kidney failure with little response to current treatments.

**Background:**

The UK National Registry of Rare Kidney Diseases Idiopathic Nephrotic Syndrome cohort includes adults and children with genetic nephrotic syndrome, FSGS, and minimal change disease. This study examines long-term patient outcomes as a function of kidney biopsy diagnosis and proteinuria control.

**Methods:**

Two thousand four hundred and sixty-seven adults and 1599 children were followed to establish outcomes including eGFR slope and kidney survival by diagnosis, analyzed as a function of proteinuria from disease onset for FSGS and minimal change disease. Enrollment began in 2010, with follow-up to September 2023. Index date for the survival analyses was date of disease onset.

**Results:**

The cohort had a median (interquartile range) follow-up of 8.2 (4.3–13.1) years; 30% of patients reached kidney failure or died. In total, 1303 patients had FSGS, 1153 had minimal change disease, and 105 had monogenic nephrotic syndrome. Children showed relatively preserved mean kidney function at disease onset (eGFR >100 ml/min per 1.73 m^2^), compared with adults (FSGS 61 ml/min per 1.73 m^2^; minimal change disease 76 ml/min per 1.73 m^2^). Kidney survival probability (95% confidence interval [CI]) at 10 years varied with diagnosis: genetic 29% (20 to 38), FSGS 58% (55 to 61), minimal change disease 87% (85 to 89) with mean (SD) rates of eGFR loss −26.5 (34.7), −6.2 (14.3), and −1.9 (10.2) ml/min per 1.73 m^2^ per year, respectively. FSGS 10-year kidney survival (95% CI) for 6–12 months lowest proteinuria value in complete remission (<0.3 g/g), partial remission (0.3–3.5 g/g), and no remission (>3.5 g/g) was 88% (70 to 96), 65% (50 to 76), and 37% (26 to 48), respectively. Time-averaged proteinuria of <1.5 g/g over 6–24 months from disease onset was associated with 90% 10-year kidney survival. For minimal change disease, patients' 10-year kidney survival (95% CI) stratified by 6–12 months lowest proteinuria value was complete remission 89% (79 to 94), partial remission 75% (51 to 89), and no remission 64% (41 to 81). In FSGS and minimal change disease, 10-year eGFR slope was strongly correlated with absolute levels of proteinuria.

**Conclusions:**

Kidney outcomes were poor in genetic nephrotic syndrome; in FSGS, outcomes were strongly associated with the proteinuria level. Patients with minimal change disease had better proteinuria control than FSGS and had better outcomes at each proteinuria level.

## Visual Abstract

**Figure:**
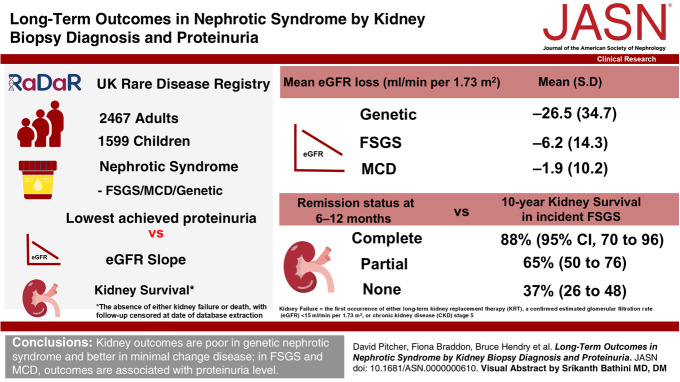

## Introduction

Idiopathic nephrotic syndrome disrupts the glomerular filtration barrier, leading to a loss of key circulating proteins into the urine.^1^ Idiopathic nephrotic syndrome is a heterogenous disease with pediatric patients classified based on steroid response and genetics and refined by kidney biopsy in steroid-resistant patients; adults are categorized primarily by their kidney histology.^1^ This results in subtypes such as monogenic nephrotic syndrome, FSGS, and minimal change disease, each guiding treatments used in clinical practice. The long-term kidney outcomes of idiopathic nephrotic syndrome and the predictive value of (changes in) proteinuria levels are not fully documented, although previous reports in certain subsets have suggested better outcomes if complete or partial remission is achieved.^2–4^ Further detailed analysis of this is of value both in providing impactful prognostic information and in designing clinical trials of new potential therapies. In this article, we systematically report the outcomes of idiopathic nephrotic syndrome in adults and children followed in the UK National Registry of Rare Kidney Diseases (RaDaR) with designation of diagnoses according to current clinical, genetic, and pathologic criteria as genetic, biopsy-confirmed primary FSGS, or minimal change disease. RaDaR included patients with idiopathic nephrotic syndrome who did not have secondary causes for proteinuria or another established glomerular disease (such as membranous nephropathy, membranoproliferative GN, or lupus nephritis).^1,5^ Patient recruitment includes retrospective and prospective follow-up with access to genetic diagnosis, kidney biopsy findings, laboratory values, and confirmed ascertainment of kidney failure and mortality.^5^ To complement our previous report of overall long-term outcomes for kidney failure and death in this idiopathic nephrotic syndrome cohort,^5^ here we provide granular data on histologically and genetically defined subgroups of idiopathic nephrotic syndrome along with correlation analysis of proteinuria and long-term outcomes in each group.

## Methods

### Data Source

This retrospective cohort study used data from the RaDaR idiopathic nephrotic syndrome cohort, for which enrollment began in 2010.^6^ Data were extracted on September 29, 2023. Patients eligible for RaDaR enrollment were children or adults with nephrotic syndrome (nephrotic range proteinuria and hypoalbuminaemia) at any stage in their disease history. Those with a biopsy diagnosis of FSGS or minimal change disease can be included if they fall in the above categories, but biopsy is not a prerequisite for inclusion. All forms of secondary nephrotic syndrome were excluded, as further explained in the Supplemental Material.

Additional details about the data source and methodology can be found in the Supplemental Methods, including a histogram of the number of patients contributing data in each calendar year (Supplemental Figure 13) and the summary statistics for number of eGFR and proteinuria results per patient (Supplemental Table 17).

### Study Population and Definitions

Summaries of the eligibility criteria, patient disposition, and study attrition are shown in Supplemental Figure 1. Subgroups of the total idiopathic nephrotic syndrome cohort were defined as patients who had a genetic diagnosis (genetic-nephrotic syndrome), patients with biopsy-proven FSGS (FSGS-biopsy; excluding confirmed genetic), patients with biopsy-proven minimal change disease, and patients who met the eligibility criteria for the study but did not have a genetic- or biopsy-confirmed diagnosis (idiopathic nephrotic syndrome–no biopsy/genetic diagnosis). Patients with an initial biopsy diagnosis of minimal change disease who also received an FSGS diagnosis on a subsequent biopsy were included in both groups and also analyzed as an additional subgroup of patients who have progressed from minimal change disease to FSGS (minimal change disease progressing to FSGS), as detailed in Supplemental Material. Disease onset was defined as the earliest of first biopsy date, primary kidney diagnosis date recorded in RaDaR, or first urine protein:creatinine ratio (UPCR) measurement ≥1.5 g/g. Kidney failure was defined as the first occurrence of either long-term KRT, a confirmed^7^ eGFR <15 ml/min per 1.73 m^2^, or CKD stage 5 recorded in RaDaR. Kidney survival was defined as the absence of either kidney failure or death, with follow-up censored at the date of database extraction. For the FSGS-biopsy and minimal change disease–biopsy subgroups, incident (baseline=disease onset) and prevalent (baseline=date of first UPCR value ≥1.5 g/g that is ≥6 months from disease onset) proteinuria analysis populations were formed to evaluate associations between proteinuria in follow-up versus eGFR slope and kidney failure/death (Supplemental Figure 2). The definitions of the achieved remission categories for proteinuria are presented in Table 1.

**Table 1:** Definitions of proteinuria remission

Proteinuria Remission Category	Proteinuria Remission Definition
Classical CR	UPCR <0.3 g/g
Classical PR	0.3 to <3.5 g/g and ≥50% reduction from baseline
Classical NR	Meeting neither classical complete nor partial remission criteria
FPR	0.3 to <1.5 g/g and ≥40% reduction from baseline
NR	Meeting none of the above remission criteria

CR, complete remission; FPR, FSGS partial remission; NR, no remission; PR, partial remission; UPCR, urine protein:creatinine ratio.

### Statistical Analyses

Kidney survival times were calculated from baseline to first kidney failure event, death from any cause, or end of follow-up for those with no event. Kaplan–Meier plots display kidney survival estimates; log-rank tests were used for comparison where appropriate. Analyses of kidney survival were conducted using Cox regression. Association between proteinuria and eGFR slope adjusting for covariates was estimated using linear mixed models with random intercept and slope.

eGFR slope and kidney survival analyses were adjusted for age, sex, ethnicity, baseline CKD stage, baseline UPCR category, time-averaged proteinuria category, and other factors found to have significant associations with outcome during unadjusted analysis (Supplemental Table 16). Missing data categories were included for all categorical variables so that patients with missing data were not excluded from analyses. Change in proteinuria was calculated from baseline value to either lowest proteinuria value or time-averaged proteinuria in the 6–12 and 6–24 months periods after baseline. A sensitivity analysis was performed to establish that the number of proteinuria measurements available per patient did not correlate with the relationships observed (Supplemental Figure 14). Data were analyzed using SAS9.4. A two-sided *P* value of < 0.05 was considered statistically significant with no correction for multiple comparisons.

## Results

### Cohort in Total

The idiopathic nephrotic syndrome cohort consisted of 4066 patients, comprising 2467 adults and 1599 children; key demographic and clinical characteristics (including completeness of data for each variable) for the full cohort, pediatric, and adult subgroups are presented in Table 2 and Supplemental Tables 1–3. The cohort had long follow-up (median [interquartile range (IQR)], 8.2 years [4.3–13.1]), and 30% of patients reached kidney failure or died within that time. For additional cohort information, please refer to the Supplemental Material.

**Table 2:** Demographic and clinical characteristics at disease onset and clinical outcomes during follow-up for idiopathic nephrotic syndrome patients (idiopathic nephrotic syndrome cohort)

Category	Idiopathic Nephrotic Syndrome–Genetic		FSGS-Biopsy		Minimal Change Disease–Biopsy		Idiopathic Nephrotic Syndrome–No Biopsy/Genetic Diagnosis		Minimal Change Disease Progressing to FSGS	
	*n*	%	*n*	%	*n*	%	*n*	%	*n*	%
**Age at disease onset**	105	100	1303	100	1153	100	1550	100	45	100
Median years (Q1–Q3)	0.4 (0.1–7.5)		35.5 (15.3–53.4)		34.6 (13.3–54.6)		13.6 (3.8–45.9)		20.3 (4.9–37.9)	
Pediatric at disease onset	86	82	356	27	342	30	836	54	21	47
**Sex**	105	100	1303	100	1153	100	1550	100	45	100
Female	53	50	552	42	519	45	639	41	19	42
**Ethnicity**	105	100	1303	100	1153	100	1550	100	45	100
Asian	26	25	142	11	158	14	192	12	6	13
Black	5	5	87	7	39	3	67	4	4	9
Not stated/missing	9	9	115	9	107	9	345	22	3	7
Other	6	6	39	3	37	3	58	4	1	2
White	59	56	920	71	812	70	888	57	31	69
**UPCR at disease onset**	26	25	339	26	325	28	254	16	16	36
Median, g/g (Q1–Q3)	28.5 (6.6–37.8)		5.7 (2.9–9.6)		6.6 (3.4–10.3)		6.9 (3.1–12.7)		5.0 (1.9–10.5)	
**Serum albumin at disease onset**	52	50	529	41	485	42	478	31	20	44
Median g/dl (Q1–Q3)	1.5 (1.0–2.7)		2.5 (1.9–3.4)		2.1 (1.5–2.9)		2.4 (1.7–3.6)		2.1 (1.7–2.9)	
**eGFR at disease onset**	32	31	442	34	377	33	317	21	17	38
Mean, ml/min per 1.73 m^2^ (SD)	96 (62)		71 (41)		84 (38)		84 (45)		77 (32)	
**Length of follow-up**	105	100	1303	100	1153	100	1550	100	45	100
Median, yr (Q1–Q3)	4.1 (2.1–8.6)		7.4 (2.7–13.1)		9.1 (5.4–14.6)		8.3 (5.0–12.6)		9.7 (3.8–16.7)	
**Kidney failure or death event**	105	100	1303	100	1153	100	1550	100	45	100
Yes	78	74	638	49	168	15	343	22	18	40
**Survival rate, estimate (95% CI)**	105	100	1303	100	1153	100	1550	100	45	100
1-yr	0.88 (0.80 to 0.93)		0.90 (0.88 to 0.92)		0.96 (0.95 to 0.97)		0.96 (0.95 to 0.97)		0.96 (0.83 to 0.99)	
2.5-yr	0.70 (0.61 to 0.78)		0.80 (0.78 to 0.82)		0.94 (0.92 to 0.95)		0.92 (0.90 to 0.93)		0.78 (0.63 to 0.87)	
5-yr	0.49 (0.39 to 0.58)		0.70 (0.67 to 0.72)		0.91 (0.89 to 0.93)		0.87 (0.86 to 0.89)		0.71 (0.56 to 0.82)	
10-yr	0.29 (0.20 to 0.38)		0.58 (0.55 to 0.61)		0.87 (0.85 to 0.89)		0.81 (0.78 to 0.83)		0.66 (0.50 to 0.78)	
**eGFR slope, all follow-up**	69	66	895	69	867	75	836	54	38	84
Mean, ml/min per 1.73 m^2^ (SD)	−26.5 (34.7)		−6.2 (14.3)		−1.9 (10.2)		−3.6 (15.2)		−6.1 (11.0)	
Median, ml/min per 1.73 m^2^ (Q1–Q3)	−15.8 (−35.9 to −4.0)		−3.0 (−7.0 to −0.6)		−0.8 (−2.9 to 0.9)		−1.8 (−5.5 to 0.6)		−2.3 (−8.1 to −0.2)	
**TAP, all follow-up**	55	52	812	62	828	72	864	56	39	87
Mean, g/g (SD)	20.3 (28.0)		3.6 (5.5)		2.2 (6.5)		2.8 (10.8)		6.3 (9.2)	
Median, g/g (Q1–Q3)	7.8 (1.9–29.1)		1.9 (0.7–4.1)		0.8 (0.3–2.0)		0.9 (0.3–2.9)		2.1 (0.5–8.3)	

CI, confidence interval; TAP, time-averaged proteinuria; UPCR, urine protein:creatinine ratio.

### Subgroups

**Figure 1:**
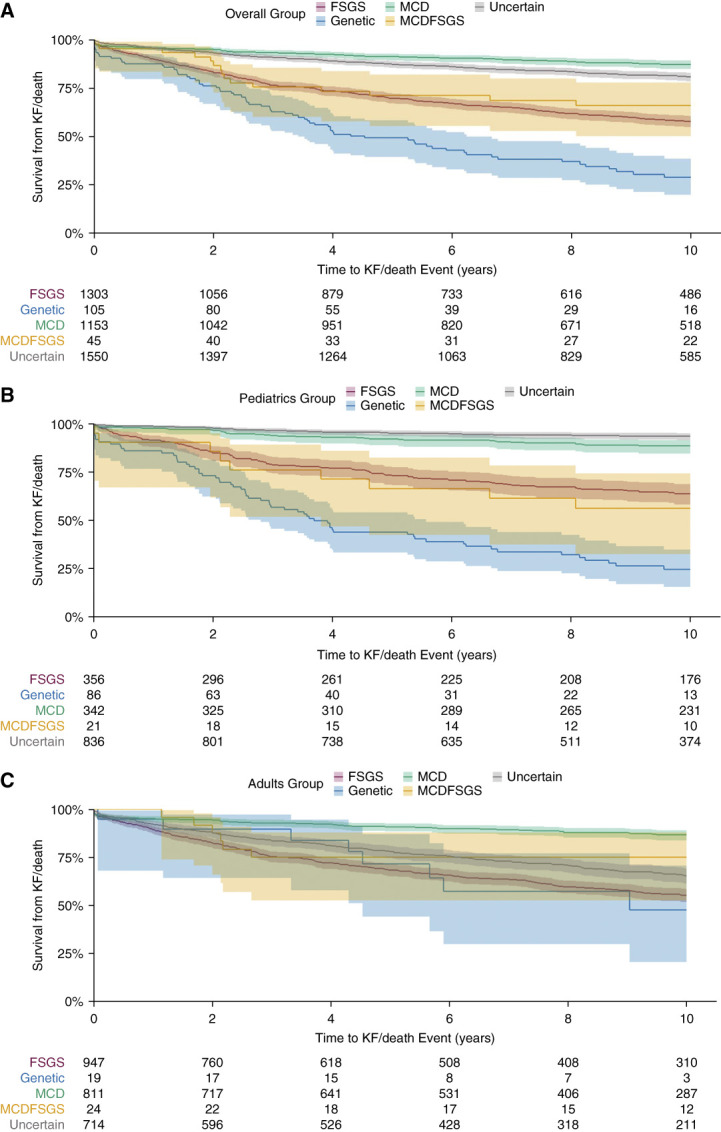
**Kaplan–Meier survival curves of time to kidney failure/death for idiopathic nephrotic syndrome patients by diagnosis category (idiopathic nephrotic syndrome cohort).** (A) Overall, (B) pediatrics, (C) adults, by diagnosis category. KF, kidney failure; MCD, minimal change disease.

Most (59%) patients in the idiopathic nephrotic syndrome cohort had a diagnosis from a kidney biopsy. There were 1303 patients with FSGS and 1153 patients with minimal change disease; one third of whom were children (Table 2). A small proportion (4%) of patients with minimal change disease (*n*=45) were confirmed as progressing to FSGS by second biopsy of a total of 116 patients with minimal change disease who had a second biopsy; further study of the new diagnoses made in these patients is ongoing. Approximately 3% of all patients (*n*=105) received a genetic diagnosis (82% children; Table 2). A proportion of patients (*n*=1550) lacked a record of a kidney biopsy or a genetic diagnosis, of whom 836 were children at disease onset; this group has uncertain diagnoses and is not analyzed in detail beyond the data presented in Figure 1, Table 2, and Supplemental Figure 3. Overall, sex and race and ethnicity distributions were similar in adults and children. Pediatric subgroups showed relatively preserved kidney function at disease onset (Supplemental Table 2), while in adults, eGFR at disease onset was much lower (Supplemental Table 3A).

### eGFR Slope and Time-Averaged Proteinuria

Figure 1 shows kidney survival estimates for the populations, and these reflect the rate of decline in eGFR over follow-up. For instance, children with genetic nephrotic syndrome showed a 10-year kidney survival rate of 25% (95% confidence interval [CI], 16 to 35; Figure 1 and Supplemental Table 2A), with mean (SD) and median (IQR) annual rates of eGFR loss (ml/min per 1.73 m^2^) at −32.7 (37.3) and −20.2 (−46.9 to −13.3), respectively (Supplemental Table 2A). By contrast, children diagnosed with minimal change disease on biopsy had a 10-year kidney survival rate estimate of 89% (95% CI, 85 to 92) with annual rates of eGFR loss (ml/min per 1.73 m^2^) at mean (SD) −4.5 (17.0) and median (IQR) −1.8 (−6.0 to 0.8; Supplemental Table 2A). The time-averaged proteinuria during follow-up seemed to correlate with survival rate estimates and the annual eGFR decline rate across different subpopulations (Table 2 and Supplemental Tables 2 and 3).

### Relationship between Proteinuria versus Disease Progression and Kidney Failure

Early changes in proteinuria, evaluated on the basis of the lowest proteinuria value and the time-averaged proteinuria, during the periods of 6–12 and 6–24 months postbaseline, were examined as categorical variables as shown in Tables 3–6 (see Supplemental Figures 4–7 and Supplemental Tables 6–8 and 11–13). On applying complete remission, classical partial remission, and FSGS partial remission (FPR) definitions of proteinuria, along with defined threshold levels, higher levels of proteinuria showed a significant association with deteriorating kidney outcomes and accelerated eGFR loss in both incident FSGS-biopsy and minimal change disease–biopsy proteinuria analysis populations. A detailed description of the proteinuria analysis populations is provided in the Supplemental Material and demographic and clinical characteristics shown in Supplemental Tables 4 and 5.

**Table 3:** FSGS-biopsy outcomes for incident populations

Incident FSGS-Biopsy	*n*	Kidney Survival Rate, Proportion (95% CI)		Kidney Failure Risk (10-yr), Hazard Ratio (95% Wald CL)		*n*	eGFR Slope, 6–30 mo (ml/min per 1.73 m^2^ per year)		*n*	eGFR Slope, 6 mo to 10 yr (ml/min per 1.73 m^2^ per year)	
LPV, 6–12 mo		5-yr	10-yr	Unadjusted	Adjusted		Mean (SD)	Median (IQR)		Mean (SD)	Median (IQR)
Combined	234	0.69 (0.62 to 0.75)	0.58 (0.50 to 0.65)	N/A	N/A	210	−8.1 (23.9)	−4.3 (−13.5 to 2.0)	219	−7.7 (22.2)	−3.0 (−9.3 to 0.4)
**Classical**											
CR	56	0.96 (0.85 to 0.99)	0.88 (0.70 to 0.96)	0.10 (0.04–0.27)	0.10 (0.04–0.29)	51	−1.5 (19.6)	0.1 (−7.4 to 2.7)	53	−0.9 (17.7)	−1.2 (−4.1 to 1.9)
PR	86	0.71 (0.59 to 0.80)	0.65 (0.50 to 0.76)	0.45 (0.28–0.74)	0.52 (0.31–0.88)	79	−5.0 (17.9)	−3.0 (−12.3 to 3.7)	82	−4.6 (15.9)	−2.0 (−7.2 to 1.2)
NR	92	0.52 (0.41 to 0.62)	0.37 (0.26 to 0.48)	Ref	Ref	80	−15.2 (29.3)	−7.1 (−20.0 to −1.7)	84	−15.1 (27.5)	−8.1 (−19.8 to −1.6)
**FPR**											
CR	56	0.96 (0.85 to 0.99)	0.88 (0.70 to 0.96)	0.11 (0.04–0.30)	0.11 (0.04–0.30)	51	−1.5 (19.6)	0.1 (−7.4 to 2.7)	53	−0.9 (17.7)	−1.2 (−4.1 to 1.9)
FPR	55	0.81 (0.67 to 0.90)	0.75 (0.56 to 0.87)	0.34 (0.17–0.66)	0.38 (0.19–0.77)	51	−3.8 (20.6)	−0.5 (−8.7 to 5.0)	52	−3.3 (18.9)	−1.1 (−4.8 to 1.8)
N-FPR	123	0.53 (0.44 to 0.62)	0.40 (0.30 to 0.50)	Ref	Ref	108	−13.1 (26.1)	−6.9 (−16.9 to −1.6)	114	−12.9 (24.2)	−7.2 (−15.8 to −1.8)
**Threshold, g/g**											
<0.3	56	0.96 (0.85 to 0.99)	0.88 (0.70 to 0.96)	0.09 (0.03–0.25)	0.08 (0.03–0.22)	51	−1.5 (19.6)	0.1 (−7.4 to 2.7)	53	−0.9 (17.7)	−1.2 (−4.1 to 1.9)
0.3 to <1.5	68	0.74 (0.61 to 0.84)	0.67 (0.51 to 0.79)	0.38 (0.21–0.69)	0.30 (0.15–0.57)	61	−3.9 (19.0)	−1.7 (−7.8 to 4.7)	64	−3.7 (17.2)	−1.5 (−5.9 to 1.7)
1.5 to <3.5	55	0.55 (0.39 to 0.68)	0.46 (0.30 to 0.60)	0.68 (0.41–1.15)	0.50 (0.27–0.92)	47	−6.2 (15.8)	−5.4 (−13.8 to 1.5)	50	−6.3 (12.9)	−5.4 (−9.3 to −0.4)
≥3.5	55	0.51 (0.37 to 0.63)	0.35 (0.21 to 0.48)	Ref	Ref	51	−21.2 (33.1)	−12.4 (−27.5 to −2.3)	52	−21.0 (31.8)	−13.0 (−25.8 to −2.7)

The analysis includes complete remission, FSGS partial remission, and threshold approaches, using the lowest proteinuria value within 6–12 months postbaseline. CI, confidence interval; CL, confidence limit; CR, complete remission; FPR, FSGS partial remission; IQR, interquartile range; LPV, lowest proteinuria value; N/A, not applicable; N-FPR, no FSGS partial remission; NR, no remission; PR, partial remission.

**Table 4:** Minimal change disease–biopsy outcomes for incident populations

Incident MCD-Biopsy	*n*	Kidney Survival Rate, Proportion (95% CI)		Kidney Failure Risk (10-yr), Hazard Ratio (95% Wald CL)		*n*	eGFR Slope, 6–30 mo (ml/min per 1.73 m^2^ per year)		*n*	eGFR Slope, 6 mo to 10 yr (ml/min per 1.73 m^2^ per year)	
LPV, 6–12 mo		5-yr	10-yr	Unadjusted	Adjusted		Mean (SD)	Median (IQR)		Mean (SD)	Median (IQR)
Combined	228	0.90 (0.85 to 0.93)	0.83 (0.74 to 0.88)	N/A	N/A	204	−3.0 (11.1)	−0.8 (−8.7 to 3.3)	215	−2.9 (7.2)	−1.0 (−4.6 to 0.6)
**Classical**											
CR	151	0.94 (0.88 to 0.97)	0.89 (0.79 to 0.94)	0.21 (0.08–0.55)	0.16 (0.04–0.57)	135	−1.8 (9.8)	0.0 (−6.2 to 3.7)	142	−1.9 (5.8)	−0.9 (−3.2 to 0.6)
PR	51	0.83 (0.68 to 0.92)	0.75 (0.51 to 0.89)	0.54 (0.20–1.44)	0.33 (0.08–1.30)	46	−5.0 (10.0)	−2.9 (−11.0 to 1.5)	49	−4.6 (9.1)	−1.3 (−6.9 to 0.5)
NR	26	0.80 (0.57 to 0.91)	0.64 (0.41 to 0.81)	Ref	Ref	23	−5.8 (17.9)	−4.0 (−21.0 to 4.8)	24	−5.3 (9.5)	−3.5 (−9.0 to 1.6)
**FPR**											
CR	151	0.94 (0.88 to 0.97)	0.89 (0.79 to 0.94)	0.23 (0.10–0.51)	0.18 (0.07–0.52)	135	−1.8 (9.8)	0.0 (−6.2 to 3.7)	142	−1.9 (5.8)	−0.9 (−3.2 to 0.6)
FPR	31	0.92 (0.72 to 0.98)	0.74 (0.26 to 0.93)	0.34 (0.1–1.2)	0.23 (0.05–0.94)	29	−2.1 (7.5)	−0.7 (−6.5 to 2.7)	30	−2.1 (4.3)	−0.8 (−4.1 to 0.5)
N-FPR	46	0.76 0.59 to 0.86)	0.66 (0.48 to 0.79)	Ref	Ref	40	−7.6 (15.6)	−6.7 (−18.2 to 1.1)	43	−6.7 (11.0)	−4.2 (−10.9 to 1.1)
**Threshold, g/g**											
<0.3	151	0.94 (0.88 to 0.97)	0.89 (0.79 to 0.94)	0.13 (0.05–0.33)	0.07 (0.02–0.28)	135	−1.8 (9.8)	0.0 (−6.2 to 3.7)	142	−1.9 (5.8)	−0.9 (−3.2 to 0.6)
0.3 to <1.5	36	0.93 (0.75 to 0.98)	0.75 (0.26 to 0.94)	0.17 (0.04–0.65)	0.08 (0.01–0.44)	34	−1.5 (7.2)	−0.2 (−6.2 to 3.0)	35	−1.8 (4.1)	−0.6 (−4.1 to 1.0)
1.5 to <3.5	26	0.78 (0.55 to 0.90)	0.73 (0.49 to 0.87)	0.43 (0.14–1.27)	0.28 (0.08–1.05)	22	−11.8 (14.0)	−10.6 (−20.3 to −1.9)	24	−6.8 (12.1)	−4.2 (−11.6 to 1.2)
≥3.5	15	0.65 (0.34 to 0.84)	0.48 (0.21 to 0.72)	Ref	Ref	13	−4.1 (18.9)	−7.7 (−15.3 to −0.1)	14	−9.0 (10.5)	−6.9 (−15.3 to −1.2)

The analysis includes complete remission, FSGS partial remission, and threshold approaches, using the lowest proteinuria value within 6–12 months postbaseline. CI, confidence interval; CL, confidence limit; CR, complete remission; FPR, FSGS partial remission; IQR, interquartile range; LPV, lowest proteinuria value; MCD, minimal change disease; N/A, not applicable; N-FPR, no FSGS partial remission; NR, no remission; PR, partial remission.

**Table 5:** Clinical outcomes for incident FSGS-biopsy proteinuria analysis populations: complete remission, FSGS partial remission, and threshold approaches applying time-averaged proteinuria within 6–24 months postbaseline

Incident FSGS-Biopsy	*n*	Kidney Survival Rate, Proportion (95% CI)		Kidney Failure Risk (10-yr), Hazard Ratio (95% Wald CL)		*n*	eGFR Slope, 6–30 mo (ml/min per 1.73 m^2^ per year)		*n*	eGFR Slope, 6 mo to 10 yr (ml/min per 1.73 m^2^ per year)	
TAP, 6–24 mo		5-yr	10-yr	Unadjusted	Adjusted		Mean (SD)	Median (IQR)		Mean (SD)	Median (IQR)
Combined	277	0.67 (0.61 to 0.73)	0.55 (0.48 to 0.62)	N/A	N/A	242	−8.4 (23.8)	−4.6 (−13.8 to 2.0)	256	−7.7 (20.9)	−3.5 (−9.2 to 0.2)
**Classical**											
CR	7	1.00 (1.00 to 1.00)	1.00 (1.00 to 1.00)	0 (0–NE)	0 (0–NE)	6	−7.6 (6.1)	−6.5 (−11.1 to −2.7)	6	−2.3 (1.0)	−2.0 (−2.7 to −1.4)
PR	100	0.93 (0.85 to 0.97)	0.88 (0.76 to 0.94)	0.12 (0.06–0.25)	0.12 (0.06–0.27)	95	−1.2 (17.2)	−0.2 (−7.4 to 4.7)	97	−0.2 (15.0)	−0.7 (−4.1 to 2.5)
NR	170	0.53 (0.45 to 0.60)	0.38 (0.30 to 0.47)	Ref	Ref	141	−13.4 (26.7)	−6.7 (−17.8 to 0.1)	153	−12.6 (23.1)	−6.6 (−15.8 to −1.6)
**FPR**											
CR	7	1.00 (1.00 to 1.00)	1.00 (1.00 to 1.00)	0 (0–NE)	0 (0–NE)	6	−7.6 (6.1)	−6.5 (−11.1 to −2.7)	6	−2.3 (1.0)	−2.0 (−2.7 to −1.4)
FPR	49	1.00 (1.00 to 1.00)	1.00 (1.00 to 1.00)	0 (0–NE)	0 (0–NE)	49	1.5 (15.7)	1.3 (−2.8 to 5.0)	49	2.2 (12.8)	0.3 (−2.4 to 3.4)
N-FPR	221	0.60 (0.53 to 0.66)	0.46 (0.38 to 0.54)	Ref	Ref	187	−11.0 (25.2)	−5.9 (15.8 to 1.0)	201	−10.2 (22.1)	−4.9 (−13.1 to −0.5)
**Threshold, g/g**											
<0.3	7	1.00 (1.00 to 1.00)	1.00 (1.00 to 1.00)	0 (0–NE)	0 (0–NE)	6	−7.6 (6.1)	−6.5 (−11.1 to −2.7)	6	−2.3 (1.0)	−2.0 (−2.7 to −1.4)
0.3 to <1.5	62	0.92 (0.80 to 0.97)	0.89 (0.75 to 0.95)	0.12 (0.05–0.30)	0.06 (0.02–0.16)	60	1.2 (14.5)	0.8 (−4.3 to 5.4)	60	1.2 (11.8)	−0.3 (−3.6 to 2.6)
1.5 to <3.5	101	0.65 (0.54 to 0.74)	0.58 (0.46 to 0.68)	0.60 (0.39–0.91)	0.37 (0.23–0.61)	82	−7.8 (19.1)	−5.7 (−12.3 to 1.0)	90	−5.1 (14.4)	−3.1 (−7.3 to 0.5)
≥3.5	107	0.54 (0.44 to 0.63)	0.36 (0.25 to 0.46)	Ref	Ref	94	−15.2 (30.1)	−8.8 (−25.0 to 0.1)	100	−15.6 (27.2)	−8.2 (−20.2 to −1.6)

CI, confidence interval; CL, confidence limit; CR, complete remission; FPR, FSGS partial remission; IQR, interquartile range; N/A, not applicable; NE, not estimable (no events in group); N-FPR, no FSGS partial remission; NR, no remission; PR, partial remission; TAP, time-averaged proteinuria.

**Table 6:** Clinical outcomes for incident minimal change disease–biopsy proteinuria analysis populations: complete remission, FSGS partial remission, and threshold approaches applying time-averaged proteinuria within 6–24 months postbaseline

Incident MCD-Biopsy	*n*	Kidney Survival Rate, Proportion (95% CI)		Kidney Failure Risk (10-yr), Hazard Ratio (95% Wald CL)		*n*	eGFR Slope, 6–30 mo (ml/min per 1.73 m^2^ per year)		*n*	eGFR Slope, 6 mo to 10 yr (ml/min per 1.73 m^2^ per year)	
TAP, 6–24 mo		5-yr	10-yr	Unadjusted	Adjusted		Mean (SD)	Median (IQR)		Mean (SD)	Median (IQR)
Combined	260	0.90 (0.85 to 0.93)	0.82 (0.75 to 0.88)	N/A	N/A	231	−3.7 (14.0)	−1.0 (−9.1 to 3.3)	244	−3.0 (8.7)	−1.0 (−4.6 to 0.6)
**Classical**											
CR	19	0.92 (0.57 to 0.99)	0.92 (0.57 to 0.99)	0.30 (0.04–2.29)	0.65 (0.08–5.27)	17	−5.4 (11.1)	−5.8 (−9.1 to 2.6)	18	−2.8 (5.9)	−1.6 (−5.8 to 1.8)
PR	168	0.94 (0.89 to 0.97)	0.87 (0.76 to 0.93)	0.30 (0.15–0.63)	0.20 (0.09–0.46)	146	−2.1 (8.7)	−0.1 (−6.3 to 3.4)	156	−2.2 (6.0)	−0.8 (−3.3 to 0.5)
NR	73	0.81 (0.69 to 0.88)	0.71 (0.57 to 0.81)	Ref	Ref	68	−6.7 (21.6)	−3.1 (−15.1 to 2.2)	70	−5.1 (13.1)	−1.7 (−7.7 to 1.2)
**FPR**											
CR	19	0.92 (0.57 to 0.99)	0.92 (0.57 to 0.99)	0.39 (0.05–2.85)	0.77 (0.09–6.27)	17	−5.4 (11.1)	−5.8 (−9.1 to 2.6)	18	−2.8 (5.9)	−1.6 (−5.8 to 1.8)
FPR	105	0.99 (0.91 to 1.00)	0.88 (0.67 to 0.96)	0.21 (0.07–0.59)	0.19 (0.06–0.58)	96	−1.1 (7.4)	0.2 (−4.2 to 3.4)	99	−1.0 (4.2)	−0.6 (−2.2 to 0.6)
N-FPR	136	0.84 (0.76 to 0.89)	0.77 (0.67 to 0.84)	Ref	Ref	118	−5.5 (17.8)	−2.5 (−13.1 to 3.0)	127	−4.7 (11.1)	−1.6 (−7.0 to 0.4)
**Threshold, g/g**											
<0.3	19	0.92 (0.57 to 0.99)	0.92 (0.57 to 0.99)	0.22 (0.03–1.65)	0.33 (0.04–2.87)	17	−5.4 (11.1)	−5.8 (−9.1 to 2.6)	18	−2.8 (5.9)	−1.6 (−5.8 to 1.8)
0.3 to <1.5	111	0.99 (0.92 to 1.00)	0.89 (0.70 to 0.96)	0.11 (0.04–0.32)	0.07 (0.02–0.26)	102	−0.7 (7.5)	0.4 (−3.4 to 3.5)	105	−1.0 (4.1)	−0.6 (−2.2 to 0.6)
1.5 to <3.5	82	0.88 (0.78 to 0.94)	0.84 (0.71 to 0.91)	0.37 (0.17–0.81)	0.28 (0.12–0.66)	68	−6.8 (16.1)	−2.8 (−13.2 to 2.7)	75	−4.7 (9.5)	−1.3 (−7.0 to 0.2)
≥3.5	48	0.73 (0.58 to 0.84)	0.62 (0.46 to 0.75)	Ref	Ref	44	−5.1 (20.8)	−4.2 (−14.3 to 0.9)	46	−5.0 (13.8)	−2.2 (−9.0 to 1.3)

CI, confidence interval; CL, confidence limit; CR, complete remission; FPR, FSGS partial remission; IQR, interquartile range; MCD, minimal change disease; N/A, not applicable; N-FPR, no FSGS partial remission; NR, no remission; PR, partial remission; TAP, time-averaged proteinuria.

**Figure 2:**
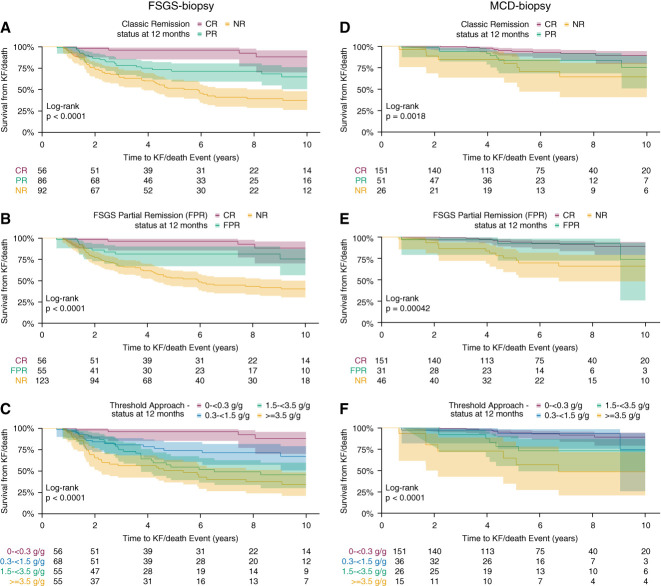
**Kaplan–Meier survival curves for incident biopsy populations.** FSGS using (A) classical remission, (B) FPR, (C) threshold approach, and minimal change disease using (D) classical remission, (E) FPR, and (F) threshold approaches. All analyses were based on the LPV recorded within 6–12 months postbaseline. CR, complete remission; FPR, FSGS partial remission; LPV, lowest proteinuria value; NR, no remission; PR, partial remission.

Figure 2 and Tables 3 and 4 present the Kaplan–Meier survival curves and clinical outcomes for the incident FSGS and minimal change disease populations. In patients with incident FSGS, those whose lowest 6- to 12-month proteinuria value did not meet classical complete remission or partial remission definitions had a 10-year kidney survival rate of 37% (95% CI, 26 to 48), compared with 65% (95% CI, 50 to 76) for partial remission and 88% (95% CI, 70 to 96) for complete remission (Figure 2A and Table 3). Mean (SD) annual eGFR decline between 6 months and 10 years for the FSGS classical no remission group was −15.1 (27.5) ml/min per 1.73 m^2^ compared with −4.6 (15.9) ml/min per 1.73 m^2^ and −0.9 (17.7) ml/min per 1.73 m^2^ for the partial remission and complete remission groups, respectively (Table 3).

**Figure 3:**
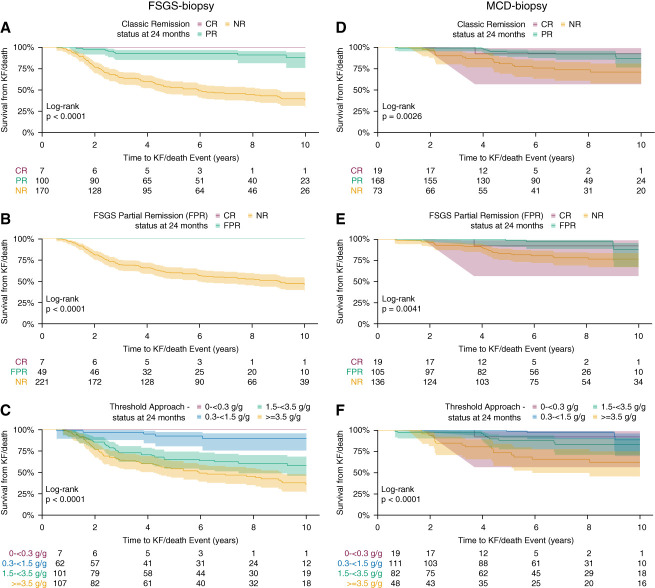
**Kaplan–Meier survival curves for incident biopsy populations.** FSGS using (A) classical remission, (B) FPR, (C) threshold approach, and minimal change disease using (D) classical remission, (E) FPR, and (F) threshold approaches. All analyses were based on the TAP within 6–24 months postbaseline. TAP, time-averaged proteinuria.

Figure 3 and Tables 5 and 6 present survival curves and clinical outcomes for two biopsy populations, incident FSGS-biopsy and minimal change disease–biopsy after the application of time-averaged proteinuria within 6–24 months postbaseline. Stratifying by time-averaged proteinuria over 6–24 months, only 3% of patients with FSGS attained complete remission, while 18% achieved FPR (<1.5 g/g); the 10-year survival rate of patients achieving complete remission or FPR was 100% compared with 46% for the no FPR group (Figure 3B and Table 5). Comparable associations were noted for the prevalent cohort of patients with FSGS, although the 10-year survival of patients achieving FPR by time-averaged proteinuria over 6–24 months was lower at 82% (95% CI, 63 to 92; Supplemental Figure 12B and Supplemental Table 13A).

Relationships between early change in proteinuria versus eGFR loss and kidney survival were evident in minimal change disease incident and prevalent populations; however, the associations were not as marked as compared with the FSGS populations (*e.g*., Figure 2 and Tables 3 and 4, also Supplemental Tables 6–12). Most (66%) patients with incident minimal change disease achieved a lowest proteinuria value <0.3 g/g within 6–12 months, with a 10-year survival in this group of 89% (95% CI, 79 to 94; Table 4), comparable with that of patients with incident FSGS achieving this lowest proteinuria value category (88% [95% CI, 70 to 96]; Table 3). Rates of eGFR loss in these complete remission groups were similar (mean [SD] 6 months to 10 years slope −1.9 [5.8] ml/min per 1.73 m^2^ per year for minimal change disease and −0.9 [17.7] ml/min per 1.73 m^2^ per year for FSGS).

The small proportion (11%) of patients with minimal change disease failing to achieve either complete or partial remission by lowest proteinuria value tended to have a better 10-year kidney survival (64% [95% CI, 41 to 81] than their FSGS counterparts (37% [95% CI, 26 to 48]; Figure 2 and Tables 3 and 4). This was reflected in slower progression of minimal change disease than FSGS no remission patients with a mean (SD) annual decline of eGFR over 6 months to 10 years of −5.3 (9.5) ml/min per 1.73 m^2^ per year, compared with −15.1 (27.5) ml/min per 1.73 m^2^ per year, respectively (Tables 3 and 4).

Applying a categorical threshold-based end point approach for the lowest proteinuria value within 6–12 months (Tables 3 and 4) and time-averaged proteinuria within 6–24 months postbaseline for the incident FSGS-biopsy (Table 5) and minimal change disease–biopsy (Table 6), proteinuria analysis populations demonstrated a robust correlation between the categorical thresholds and eGFR decline, as well as the risk of kidney failure. Slower decline in eGFR and better outcomes for patients with FSGS achieving time-averaged proteinuria in all the categories <1.5 versus ≥1.5 g/g is seen.

### Relationship between Percent Change in Proteinuria versus Disease Progression and Kidney Failure

**Figure 4:**
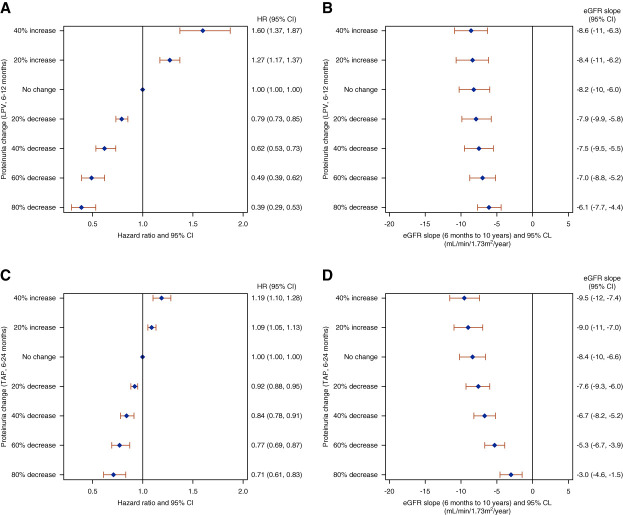
**Forest plots of FSGS-biopsy proteinuria analysis population.** Percentage change from baseline for LPV within 6–12 months postbaseline versus (A) hazard ratio for kidney failure/death event and versus (B) eGFR slope over 6 months to 10 years. Percentage change from baseline for TAP within 6–24 months postbaseline versus (C) hazard ratio for kidney failure/death event and versus (D) eGFR slope over 6 months to 10 years. CI, confidence interval; CL, confidence limit; HR, hazard ratio.

The early change in proteinuria, evaluated through lowest proteinuria value and time-averaged proteinuria, spanning 6–12 and 6–24 months postbaseline, was analyzed as a continuous variable by percentage. Figure 4 shows forest plots of FSGS-biopsy proteinuria analysis population using percentage change from baseline for lowest proteinuria value within 6–12 months postbaseline with outcomes related to kidney failure/death events and eGFR slope over varying durations. After categorizing by the percentage change from baseline, declining proteinuria was associated with slower loss of eGFR and lower risk of kidney failure outcomes in both the FSGS and minimal change disease populations.

In patients with incident FSGS, the adjusted hazard ratio of kidney failure for a 40% reduction in lowest proteinuria value over 6–12 months was 0.62 (95% CI, 0.53 to 0.73) compared with those exhibiting no change in proteinuria. However, with time-averaged proteinuria over 6–24 months, the reduction in risk was less pronounced, with a 40% reduction in proteinuria associated with a hazard ratio of 0.84 (95% CI, 0.78 to 0.91) (Figure 4). Proteinuria change (assessed using either time-averaged proteinuria over 6–24 months or lowest proteinuria value over 6–12 months) was also significantly associated with 6-month to 10-year eGFR slope; patients with greater reductions in proteinuria had significantly (*P* < 0.001) slower decline in eGFR across 6 months to 10 years (Figure 4). Similar findings were noted in the prevalent FSGS population (see Supplemental Figure 8).

In the prevalent minimal change disease population, percentage change from baseline in time-averaged proteinuria over 6–24 months was more strongly associated with kidney failure than percentage change from baseline in lowest proteinuria value over 6–12 months (Supplemental Figure 10); adjusted hazard ratios (95% CI) for a 40% reduction were 0.72 (0.66 to 0.78) and 0.87 (0.80 to 0.95), respectively (Supplemental Figure 10). Although in minimal change disease, significant associations with 6-month to 10-year eGFR slope were observed for percentage change for both time-averaged proteinuria over 6–24 months (*P* value < 0.001) and lowest proteinuria value over 6–12 months (*P* value = 0.007), the effect sizes were small.

In the incident minimal change disease population, no significant association between kidney failure and change from baseline to lowest proteinuria value over 6–12 months was observed; however, there was a significant association when assessing change from baseline to time-averaged proteinuria over 6–24 months where a 40% reduction had an adjusted hazard ratio (95% CI) of 0.59 (0.45 to 0.77; Supplemental Figure 9). No significant associations between proteinuria change and 6-month to 10-year eGFR slope were observed in the incident minimal change disease population.

### Relationship between Incremental Proteinuria Response Thresholds and Disease Progression in Patients with FSGS

**Figure 5:**
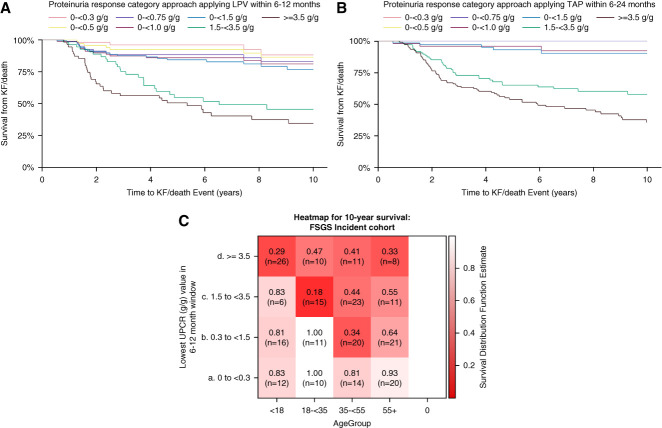
**Kaplan–Meier survival curves of incident FSGS-biopsy proteinuria analysis population.** Proteinuria response category approach applying (A) lowest proteinuria value within 6–12 months postbaseline, (B) time-averaged proteinuria within 6–24 months postbaseline, and (C) heatmap for 10-year survival within 6–12 months postbaseline. UPCR, urine protein:creatinine ratio.

Figure 5 shows Kaplan–Meier survival curves for the incident FSGS-biopsy proteinuria analysis population, using lowest proteinuria value between 6 and 12 months postbaseline, and time-averaged proteinuria values between 6 and 24 months postbaseline. Considering the robust correlation between the categorical threshold-based end point approach and eGFR decline, as well as the risk of kidney failure in patients with FSGS, an exploratory analysis was undertaken to investigate how incrementally higher cut-points for proteinuria cut-points (incremental proteinuria response thresholds), commencing at <0.3 g/g (corresponding to complete remission of proteinuria), were associated with the risk of kidney failure. Applying the lowest proteinuria value over 6–12 months among the incident FSGS population with a baseline proteinuria of 6.1 (95% CI, 3.7 to 9.7) g/g, 36% and 53% of patients achieved response thresholds of <0.75 and <1.5 g/g, respectively (Table 7A). This was accompanied by associated 10-year kidney survival estimates of 83% (95% CI, 70 to 91) and 77% (95% CI, 65 to 85; Figure 5 and Table 7A). Results applying lowest proteinuria value over 6–24 months and time-averaged proteinuria over 6–12 months are shown in Supplemental Figure 11 and Supplemental Tables 9 and 10, and results for the prevalent FSGS population shown in Supplemental Tables 14 and 15.

**Table 7:** Clinical outcomes for incident FSGS-biopsy proteinuria analysis population: proteinuria response category approach applying lowest proteinuria value within 6–12 months postbaseline (A) and time-averaged proteinuria within 6–24 months postbaseline (B)

(A)										
LPV, 6–12 mo	*n* (%)	Proteinuria at Disease Onset (g/g)	Kidney Failure or Death Event	Kidney Survival Rate, Proportion (95% CI)		Kidney Failure Risk (10-yr), Hazard Ratio (95% Wald CL)		*n*	eGFR Slope, 6 mo to 10 yr (ml/min per 1.73 m^2^ per year)	
		Median (Q1–Q3)	*n* (%)	5-yr	10-yr	Unadjusted	Adjusted		Mean (SD)	Median (IQR)
Combined	234 (100)	6.1 (3.7–9.7)	79 (34)	0.69 (0.62 to 0.75)	0.58 (0.50 to 0.65)	N/A	N/A	222	−7.1 (21.1)	−3.0 (−8.7 to 0.3)
<0.3 g/g	56 (24)	7.1 (4.4–10.4)	4 (7)	0.96 (0.85 to 0.99)	0.88 (0.70 to 0.96)	0.09 (0.03–0.26)	0.09 (0.03–0.25)	53	0.7 (12.8)	−1.2 (−3.5 to 2.0)
<0.5 g/g	72 (31)	7.1 (4.1–10.4)	7 (10)	0.92 (0.83 to 0.97)	0.86 (0.72 to 0.94)	0.13 (0.06–0.28)	0.11 (0.05–0.26)	69	−0.2 (12.0)	−1.2 (−4.0 to 2.0)
<0.75 g/g	85 (36)	6.6 (3.7–10.0)	11 (13)	0.88 (0.79 to 0.94)	0.83 (0.70 to 0.91)	0.18 (0.09–0.36)	0.16 (0.08–0.33)	80	−0.6 (13.8)	−1.4 (−4.1 to 1.8)
<1.0 g/g	96 (41)	6.5 (3.7–9.7)	14 (15)	0.86 (0.76 to 0.92)	0.81 (0.69 to 0.89)	0.21 (0.11–0.39)	0.19 (0.10–0.36)	91	−1.7 (17.4)	−1.4 (−4.4 to 2.0)
<1.5 g/g	124 (53)	6.1 (3.3–9.3)	21 (17)	0.84 (0.76 to 0.90)	0.77 (0.65 to 0.85)	0.24 (0.14–0.41)	0.22 (0.13–0.39)	119	−1.9 (15.3)	−1.4 (−4.7 to 1.7)
1.5 to <3.5 g/g	55 (24)	4.3 (3.0–7.0)	25 (45)	0.55 (0.39 to 0.68)	0.46 (0.30 to 0.60)	0.69 (0.41–1.17)	0.68 (0.39–1.16)	51	−6.3 (13.2)	−5.4 (−10.2 to −0.1)
≥3.5 g/g	55 (24)	8.2 (5.3–12.5)	33 (60)	0.51 (0.37 to 0.63)	0.35 (0.21 to 0.48)	Ref	Ref	52	−19.6 (31.6)	−11.1 (−24.3 to −2.6)

(B)										
TAP, 6–24 mo	*n* (%)	Proteinuria at Disease Onset (g/g)	Kidney Failure or Death Event	Kidney Survival Rate, Proportion (95% CI)		Kidney Failure Risk (10-yr), Hazard Ratio (95% Wald CL)		*n*	eGFR Slope, 6 mo to 10 yr (ml/min per 1.73 m^2^ per year)	
		Median (Q1–Q3)	*n* (%)	5-yr	10-yr	Unadjusted	Adjusted		Mean (SD)	Median (IQR)
Combined	277 (100)	6.1 (3.6–10.0)	101 (36)	0.67 (0.61 to 0.73)	0.55 (0.48 to 0.62)	N/A	N/A	259	−7.1 (20.0)	−3.1 (−9.2 to 0.1)
<0.3 g/g	7 (3)	4.5 (1.6–5.7)	0 (0)	1.00 (1.00 to 1.00)	1.00 (1.00 to 1.00)	0.00 (0.00–NE)	0.00 (0.00–NE)	6	−2.1 (1.2)	−2.0 (−2.7 to −1.4)
<0.5 g/g	14 (5)	4.1 (3.6–5.3)	0 (0)	1.00 (1.00 to 1.00)	1.00 (1.00 to 1.00)	0.00 (0.00–NE)	0.00 (0.00–NE)	13	−1.3 (2.8)	−1.9 (−2.7 to −0.7)
<0.75 g/g	28 (10)	4.4 (2.8–7.1)	0 (0)	1.00 (1.00 to 1.00)	1.00 (1.00 to 1.00)	0.00 (0.00–NE)	0.00 (0.00–NE)	27	−0.4 (3.2)	−1.2 (−2.7 to 3.1)
<1.0 g/g	51 (18)	5.3 (3.2–8.6)	3 (6)	0.96 (0.85 to 0.99)	0.92 (0.77 to 0.98)	0.09 (0.03–0.29)	0.09 (0.03–0.28)	49	1.5 (12.9)	−1.2 (−2.7 to 2.1)
<1.5 g/g	69 (25)	5.0 (2.6–7.6)	5 (7)	0.93 (0.82 to 0.97)	0.90 (0.77 to 0.96)	0.11 (0.04–0.27)	0.10 (0.04–0.25)	67	0.8 (11.2)	−0.7 (−3.5 to 2.1)
1.5 to <3.5 g/g	101 (36)	4.3 (2.8–7.6)	35 (35)	0.65 (0.54 to 0.74)	0.58 (0.46 to 0.68)	0.60 (0.39–0.91)	0.57 (0.38–0.87)	92	−4.5 (11.5)	−3.4 (−7.4 to −0.2)
≥3.5 g/g	107 (39)	8.9 (5.6–15.7)	61 (57)	0.54 (0.44 to 0.63)	0.36 (0.25 to 0.46)	Ref	Ref	100	−14.7 (27.1)	−7.1 (−19.3 to −1.4)

CI, confidence interval; CL, confidence limit; IQR, interquartile range; LPV, lowest proteinuria value; N/A, not applicable; NE, not estimable (no events in group); TAP, time-averaged proteinuria.

## Discussion

This study highlights the significant burden of idiopathic nephrotic syndrome; one third of 4066 patients reached kidney failure or died over a median 8.2-year follow-up. There were 102 deaths before kidney failure, comprising 8% of events. The analysis of subgroups, particularly focusing on FSGS and minimal change disease, provides valuable insights into the clinical course and outcomes of these distinct entities within idiopathic nephrotic syndrome.

The analysis highlights that proteinuria is an early marker associated with disease progression and kidney outcomes. FSGS and minimal change disease differ in clinical presentation, trajectory, and treatment response. Patients with monogenic nephrotic syndrome respond poorly to current treatments, linking glomerular filtration barrier damage to outcomes.^8^ This is evident from the highest proteinuria levels at onset, kidney failure risk rate, and eGFR decline.^9,10^ High proteinuria levels in this cohort prevented a quantitative analysis of the relationship between proteinuria and outcomes within the group. Potential ascertainment bias exists, as severe cases are more likely tested for genetic causes.

Many idiopathic nephrotic syndrome cohort patients lacked biopsy or genetic diagnosis. In children, this reflects clinical practice, where steroid-sensitive patients usually avoid biopsy; most would likely have minimal change disease if biopsies were performed. Infrequent kidney failure events in nonbiopsied children (Supplemental Table 2A) suggest milder disease. In adults, missing biopsy data likely indicate contraindications, technical failures, or unrecorded biopsies in RaDaR. In view of the diagnostic uncertainty, this no-diagnosis group was not analyzed by proteinuria categories.

The use of the first biopsy data as disease onset in the minimal change disease progressing to FSGS group is based on the nephrologic view that these patients had a single disease detected at first biopsy but have had a delay in establishing the final diagnosis.

FSGS represents a severe form of idiopathic nephrotic syndrome, characterized by podocyte injury and glomerular sclerosis.^11,12^ Patients with FSGS face rapid eGFR decline and worse long-term outcomes compared with those with minimal change disease. Proteinuria levels exceeding 1.5 g/g, assessed by lowest proteinuria value or time-averaged proteinuria, were associated with a markedly higher kidney failure risk, consistent with clinical reports and the FSGS-specific surrogate end point, FPR.^13,14^ The study links persistent proteinuria to higher kidney failure risk and accelerated eGFR decline. The difference in eGFR slope associated with a 40% reduction in time-averaged proteinuria over the 6- to 24-month period after baseline was 1.7 ml/min per 1.73 m^2^ per year, as seen in Figure 4D. This is comparable with the results obtained by Troost *et al.*^15^ in an analysis of the FSGS Clinical Trial study, of about 2 ml/min per 1.73 m^2^ per year improvement associated with a 40% reduction in proteinuria at 26 weeks. However, the study populations and timing of data acquisition are different, so detailed comparisons should be viewed with caution. The current results are in line with the very poor outcomes reported in patients who fail to achieve any proteinuria remission below the classical threshold of <3.5 g/g.^2,14^ The idiopathic nephrotic syndrome FSGS data also concur with the prognostic value of achieving a threshold of <1.5 g/g and 40% reduction (FPR), as reported by Troost *et al*.^14^ Importantly, in the current work, achieving complete remission or FPR for time-averaged proteinuria within the first 2 years of care in patients with FSGS correlates with at least 85% 10-year kidney survival, emphasizing the importance of effective remission strategies.^14,16^

In contrast to FSGS, patients with a biopsy showing minimal change disease typically have a more favorable prognosis.^17^ Most patients with minimal change disease achieve remission of proteinuria quickly, leading to better long-term kidney outcomes compared with FSGS. Even among patients with minimal change disease who did not reach complete or partial remission, outcomes were still better than in patients with FSGS in similar proteinuria ranges. Adult patients with minimal change disease often include both steroid-sensitive and steroid-resistant individuals, while pediatric patients with minimal change disease are mostly steroid-resistant due to biopsy selection practices. Interestingly, although changes in proteinuria are not associated with eGFR decline in minimal change disease, they did correlate with a higher risk of kidney failure. This suggests that increasing proteinuria may contribute to faster time to kidney failure, even if the statistical association with eGFR decline is not evident.

A comparison between FSGS and minimal change disease reveals notable differences in disease progression and treatment response. Patients with FSGS face a more rapid decline in kidney function and higher kidney failure rates despite treatments aimed at reducing proteinuria. By contrast, patients with minimal change disease, even those not in remission, generally have better outcomes than patients with FSGS. Early differentiation of these subtypes is crucial for effective management and prognostication. A higher rate of undiagnosed genetic cases in FSGS could influence these outcomes, although the pattern is also seen in adults where genetic prevalence is lower than in children.^18^ The diagnostic criterion whereby finding a single glomerulus with segmental sclerosis can define FSGS means that certain patients diagnosed with minimal change disease may in fact have had FSGS diagnosed had more biopsy material been available, and these may be the patients with minimal change disease who do least well. Further analysis is needed for the small subset transitioning from minimal change disease to FSGS; their outcomes seem similar to those patients given an FSGS diagnosis at the outset. The subset of patients with minimal change disease who have a poor kidney failure outcome is worthy of further study. Some may have been undetected FSGS; others may in the future be given a more precise molecular diagnosis. For example, the prevalence and titer of antinephrin autoantibodies in subsets of this idiopathic nephrotic syndrome population are currently under investigation.

The study offers insights into FSGS and minimal change disease within the idiopathic nephrotic syndrome cohort, but several limitations must be considered. The retrospective design and reliance on electronic health records may introduce biases. The study population's racial and ethnic background reflects the UK population, potentially limiting generalizability. Another potential limitation is that the change in proteinuria may reflect differences in underlying pathology, which could be partly responsible for differences in eGFR loss rather than proteinuria itself.

In conclusion, the study provides valuable insights into the clinical course and outcomes of patients with biopsies showing FSGS or minimal change disease within the idiopathic nephrotic syndrome cohort, highlighting the importance of early markers such as proteinuria, which are associated with disease progression and could guide therapeutic interventions. These results highlight a difference in risk according to biopsy findings, with a considerably higher risk in patients with FSGS at a threshold proteinuria level of 1.5 g/g (both for lowest proteinuria value and time-averaged proteinuria). With minimal change disease, the risk is less at the same levels of proteinuria. This underscores the need for careful selection of patient subgroups for clinical trials, with a particular emphasis on achieving remission of proteinuria to improve long-term outcomes in patients with idiopathic nephrotic syndrome. The work presented here is being used to assist the Proteinuria and GFR as Clinical Trial Endpoints in FSGS project on end points and study design in FSGS.^19^ The findings of this study illustrate the potential for application of time-averaged proteinuria and lowest proteinuria value as clinical trial end point variables. Time-averaged proteinuria is a more complete record of proteinuria in the period of observation, while lowest proteinuria value has merit as an accessible snapshot with value in clinical practice. It is noteworthy that time-averaged proteinuria thresholds were harder to achieve (as expected), but both time-averaged proteinuria and lowest proteinuria value were associated with long-term outcomes, *e.g*., kidney survival over 10 years.

In the future, the classification of idiopathic nephrotic syndrome will be informed by increasing molecular understanding as biomarker technology improves, for example, in the identification of potential circulating factors. In particular, the discovery of antinephrin antibodies found predominantly in patients with minimal change disease could lead to improved risk stratification, alongside proteinuria responses.^20,21^ However, kidney biopsy is likely to remain a vital diagnostic tool for the foreseeable future and further refined to incorporate molecular characterisations.^22^

## Supplementary Material

## Data Availability

Data cannot be shared. Partial restrictions to the data and/or materials apply. Data cannot be shared as these are confidential clinical records. Metadata are publicly available.

## Supplemental Material

Supplemental Methods

Supplemental Results

Supplemental Table 1. Demographic and clinical characteristics at disease onset and clinical outcomes during follow-up for patients with idiopathic nephrotic syndrome (idiopathic nephrotic syndrome cohort).

Supplemental Table 2. (A) Demographic and clinical characteristics at disease onset and clinical outcomes during follow-up for pediatric patients with idiopathic nephrotic syndrome (idiopathic nephrotic syndrome cohort—pediatrics), including (B) additional demographic and clinical characteristics.

Supplemental Table 3. (A) Demographic and clinical characteristics at disease onset and clinical outcomes during follow-up for adult patients with idiopathic nephrotic syndrome (idiopathic nephrotic syndrome cohort—adults), including (B) additional demographic and clinical characteristics.

Supplemental Table 4. Demographic and clinical characteristics at disease onset and clinical outcomes during follow-up for incident FSGS-biopsy and minimal change disease–biopsy proteinuria analysis populations: overall, pediatrics, adults.

Supplemental Table 5. Demographic and clinical characteristics at disease onset and clinical outcomes during follow-up for prevalent FSGS-biopsy and minimal change disease–biopsy proteinuria analysis populations: overall, pediatrics, adults.

Supplemental Table 6. Clinical outcomes for incident (A) FSGS-biopsy and (B) minimal change disease–biopsy proteinuria analysis populations: complete remission, FPR, and threshold approaches applying lowest proteinuria value within 6–24 months postbaseline.

Supplemental Table 7. Clinical outcomes for prevalent (A) FSGS-biopsy and (B) minimal change disease–biopsy proteinuria analysis populations: complete remission, FPR, and threshold approaches applying lowest proteinuria value within 6–12 months postbaseline.

Supplemental Table 8. Clinical outcomes for prevalent (A) FSGS-biopsy and (B) minimal change disease–biopsy proteinuria analysis populations: complete remission, FPR, and threshold approaches applying lowest proteinuria value within 6–24 months postbaseline.

Supplemental Table 9. Clinical outcomes for incident FSGS-biopsy proteinuria analysis population: proteinuria response category approach applying lowest proteinuria value within 6–24 months postbaseline.

Supplemental Table 10. Clinical outcomes for incident FSGS-biopsy proteinuria analysis population: proteinuria response category approach applying time-averaged proteinuria within 6–12 months postbaseline.

Supplemental Table 11. Clinical outcomes for incident (A) FSGS-biopsy and (B) minimal change disease–biopsy proteinuria analysis populations: complete remission, FPR, and threshold approaches applying time-averaged proteinuria within 6–12 months postbaseline.

Supplemental Table 12. Clinical outcomes for prevalent (A) FSGS-biopsy and (B) minimal change disease–biopsy proteinuria analysis populations: complete remission, FPR, and threshold approaches applying time-averaged proteinuria within 6–12 months postbaseline.

Supplemental Table 13. Clinical outcomes for prevalent (A) FSGS-biopsy and (B) minimal change disease–biopsy proteinuria analysis populations: complete remission, FPR, and threshold approaches applying time-averaged proteinuria within 6–24 months postbaseline.

Supplemental Table 14. Clinical outcomes for prevalent FSGS-biopsy proteinuria analysis population: proteinuria response category approach applying lowest proteinuria value within (A) 6–12 months and (B) 6–24 months postbaseline.

Supplemental Table 15. Clinical outcomes for prevalent FSGS-biopsy proteinuria analysis population: proteinuria response category approach applying time-averaged proteinuria within (A) 6–12 months and (B) 6–24 months postbaseline.

Supplemental Table 16. Unadjusted and multivariable analysis of 10-year survival for FSGS-biopsy and minimal change disease–biopsy proteinuria analysis populations.

Supplemental Table 17. Summary statistics for (A) the number of UPCR measurements per patient during follow-up windows and (B) the number of eGFR measurements per patient used to calculate eGFR slopes.

Supplemental Figure 1. (A) Summary of the total RaDaR idiopathic nephrotic syndrome population, and (B) eligibility criteria, patient disposition, and study attrition for the incident and prevalent proteinuria analysis populations of FSGS and minimal change disease.

Supplemental Figure 2. Venn diagram displaying the degree of overlap between proteinuria analysis populations within the idiopathic nephrotic syndrome cohort (A) FSGS-biopsy subgroup and (B) minimal change disease–biopsy subgroup.

Supplemental Figure 3. Kaplan–Meier survival curves by age at kidney failure/death for idiopathic nephrotic syndrome patients: (A) idiopathic nephrotic syndrome cohort, (B) idiopathic nephrotic syndrome cohort—pediatrics, and (C) idiopathic nephrotic syndrome cohort—adults, by diagnosis category.

Supplemental Figure 4. Kaplan–Meier survival curves for incident biopsy populations: (A) FSGS classical remissions, (B) FPR, (C) FSGS threshold approach, (D) minimal change disease classical remissions, (E) minimal change disease partial remission, and (F) minimal change disease threshold approaches.

Supplemental Figure 5. Kaplan–Meier survival curves for prevalent biopsy populations: (A) FSGS classical remissions, (B) FPR, (C) FSGS threshold approach, (D) minimal change disease classical remissions, (E) minimal change disease partial remission, and (F) minimal change disease threshold approaches.

Supplemental Figure 6. Kaplan–Meier survival curves for incident biopsy populations: (A) FSGS classical remissions, (B) FPR, (C) FSGS threshold approach, (D) minimal change disease classical remissions, (E) minimal change disease partial remission, and (F) minimal change disease threshold approaches.

Supplemental Figure 7. Kaplan–Meier survival curves for prevalent biopsy populations: (A) FSGS classical remissions, (B) FPR, (C) FSGS threshold approach, (D) minimal change disease classical remissions, (E) minimal change disease partial remission, and (F) minimal change disease threshold approaches.

Supplemental Figure 8. Forest plots of prevalent FSGS-biopsy proteinuria analysis population. Percentage change from baseline for lowest proteinuria value within 6–12 months postbaseline versus (A) hazard ratio for kidney failure/death event and versus (B) eGFR slope over 6 months to 10 years. Percentage change from baseline for time-averaged proteinuria within 6–24 months postbaseline versus (C) hazard ratio for kidney failure/death event and versus (D) eGFR slope over 6 months to 10 years.

Supplemental Figure 9. Forest plots of incident minimal change disease–biopsy proteinuria analysis population. Percentage change from baseline for lowest proteinuria value within 6–12 months postbaseline versus (A) hazard ratio for kidney failure/death event and versus (B) eGFR slope over 6 months to 10 years. Percentage change from baseline for time-averaged proteinuria within 6–24 months postbaseline versus (C) hazard ratio for kidney failure/death event and versus (D) eGFR slope over 6 months to 10 years.

Supplemental Figure 10. Forest plots of prevalent minimal change disease–biopsy proteinuria analysis population. Percentage change from baseline for lowest proteinuria value within 6–12 months postbaseline versus (A) hazard ratio for kidney failure/death event and versus (B) eGFR slope over 6 months to 10 years. Percentage change from baseline for time-averaged proteinuria within 6–24 months postbaseline versus (C) hazard ratio for kidney failure/death event and versus (D) eGFR slope over 6 months to 10 years.

Supplemental Figure 11. Kaplan–Meier survival curves of incident FSGS-biopsy proteinuria analysis population: proteinuria response category approach applying (A) lowest proteinuria value within 6–24 months postbaseline and (B) time-averaged proteinuria within 6–12 months postbaseline.

Supplemental Figure 12. Kaplan–Meier survival curves of prevalent FSGS-biopsy proteinuria analysis population: proteinuria response category approach applying (A) lowest proteinuria value within 6–12 months postbaseline and (B) time-averaged proteinuria within 6–24 months postbaseline.

Supplemental Figure 13. Number of patients contributing data in each calendar year.

Supplemental Figure 14. Association between the number of UPCR measurements used in the calculation of proteinuria values and outcomes.
