# Supplementary material for: Long-Term Outcomes in Nephrotic Syndrome by Kidney Biopsy Diagnosis and Proteinuria
Source: J Am Soc Nephrol. 2025 Apr 17;36(7):1398–413. doi: 10.1681/ASN.0000000610 (PMC12187231; doi:10.1681/ASN.0000000610)
Supplement: Supplementary file 1 [file jasn-36-1398-s001.pdf]

ASN American Society of Nephrology | JASN Journal of the American Society of Nephrology | KIDNEY360 Accessing Our World From Every Angle | CJASN Clinical Journal of the American Society of Nephrology

ASN Journal Disclosure Form

As per ASN journal policy, I have disclosed any financial relationships or commitments I have held in the past 36 months as included below. I have listed my Current Employer below to indicate there is a relationship requiring disclosure. If no relationship exists, my Current Employer is not listed.

J. Barratt reports the following:
Employer: UNIVERSITY OF LEICESTER; Consultancy: Alexion, Astellas, Alebund, Alnylam, Alpine, Argenx, BioCryst, Calliditas, Chinook, Dimerix, HiBio, Kira, Novartis, Omeros, Otsuka, Travere Therapeutics, Q32 Bio, Roche, Sanofi, Takeda, Vera Therapeutics, Vifor, Visterra; Research Funding: Alexion, Novartis; GlaxoSmithKline; Calliditas, Visterra, Chinook, Omeros, Galapagos, argenx, Travere Therapeutics; and Advisory or Leadership Role: Editorial Board of Kidney International, CJASN, Glomerular Diseases & Clinical Science; Treasurer International IgA Nephropathy Network.

I understand that the information above will be published within the journal article, if accepted, and that failure to comply and/or to accurately and completely report the potential financial conflicts of interest could lead to the following: 1) Prior to publication, article rejection, or 2) Post-publication, sanctions ranging from, but not limited to, issuing a correction, reporting the inaccurate information to the authors' institution, banning authors from submitting work to ASN journals for varying lengths of time, and/or retraction of the published work.

Name: Jonathan Barratt
Manuscript ID: JASN-2024-001050R1
Manuscript Title: Long term outcomes in nephrotic syndrome: analysis by kidney biopsy diagnosis and proteinuria of UK RaDaR Registry patients
Date of Completion: November 25, 2024
Disclosure Updated Date: August 28, 2024

ASN American Society of Nephrology | JASN Journal of the American Society of Nephrology | KIDNEY360 Accessing Our World From Every Angle | CJASN Clinical Journal of the American Society of Nephrology

ASN Journal Disclosure Form

As per ASN journal policy, I have disclosed any financial relationships or commitments I have held in the past 36 months as included below. I have listed my Current Employer below to indicate there is a relationship requiring disclosure. If no relationship exists, my Current Employer is not listed.

F. Braddon reports the following:
Employer: UK Kidney Association

I understand that the information above will be published within the journal article, if accepted, and that failure to comply and/or to accurately and completely report the potential financial conflicts of interest could lead to the following: 1) Prior to publication, article rejection, or 2) Post-publication, sanctions ranging from, but not limited to, issuing a correction, reporting the inaccurate information to the authors' institution, banning authors from submitting work to ASN journals for varying lengths of time, and/or retraction of the published work.

Name: Fiona E. M. Braddon
Manuscript ID: JASN-2024-001050R1
Manuscript Title: Long-Term Outcomes in Nephrotic Syndrome: Analysis by Kidney Biopsy Diagnosis and Proteinuria of UK RaDaR Registry Patients
Date of Completion: January 2, 2025
Disclosure Updated Date: January 2, 2025

ASN American Society of Nephrology | JASN Journal of the American Society of Nephrology | KIDNEY360 Accessing Our World From Every Angle | CJASN Clinical Journal of the American Society of Nephrology

ASN Journal Disclosure Form

As per ASN journal policy, I have disclosed any financial relationships or commitments I have held in the past 36 months as included below. I have listed my Current Employer below to indicate there is a relationship requiring disclosure. If no relationship exists, my Current Employer is not listed.

D. Gale reports the following:
Employer: University College London; Consultancy: Novartis; Alexion; Calliditas; Vifor; Judo Bio; Alnylam; SOBI; Bayer; Otsuka; Apellis; GSK; Research Funding: Travere; Novartis; Honoraria: Otsuka, Sanofi, STADA; and Other Interests or Relationships: Trustee for AlportUK; Chair of UK Kidney Association Rare Diseases Committee.

I understand that the information above will be published within the journal article, if accepted, and that failure to comply and/or to accurately and completely report the potential financial conflicts of interest could lead to the following: 1) Prior to publication, article rejection, or 2) Post-publication, sanctions ranging from, but not limited to, issuing a correction, reporting the inaccurate information to the authors' institution, banning authors from submitting work to ASN journals for varying lengths of time, and/or retraction of the published work.

Name: Daniel P. Gale
Manuscript ID: JASN-2024-001050R2
Manuscript Title: Long term outcomes in nephrotic syndrome by kidney biopsy diagnosis and proteinuria
Date of Completion: March 25, 2025
Disclosure Updated Date: March 25, 2025

ASN
American Society of Nephrology
JASN
Journal of the American Society of Nephrology
KIDNEY360
Accessing Our World From Every Angle
CJASN
Clinical Journal of the American Society of Nephrology

ASN Journal Disclosure Form

As per ASN journal policy, I have disclosed any financial relationships or commitments I have held in the past 36 months as included below. I have listed my Current Employer below to indicate there is a relationship requiring disclosure. If no relationship exists, my Current Employer is not listed.

W. Gong reports the following:
Employer: Travere Therapeutics; and Ownership Interest: Travere Therapeutics.

I understand that the information above will be published within the journal article, if accepted, and that failure to comply and/or to accurately and completely report the potential financial conflicts of interest could lead to the following: 1) Prior to publication, article rejection, or 2) Post-publication, sanctions ranging from, but not limited to, issuing a correction, reporting the inaccurate information to the authors' institution, banning authors from submitting work to ASN journals for varying lengths of time, and/or retraction of the published work.

Name: Wu Gong
Manuscript ID: JASN-2024-001050R1
Manuscript Title: Long term outcomes in nephrotic syndrome: analysis by kidney biopsy diagnosis and proteinuria of UK RaDaR Registry patients
Date of Completion: November 25, 2024
Disclosure Updated Date: November 25, 2024

ASN
American Society of Nephrology
JASN
Journal of the American Society of Nephrology
KIDNEY360
Accessing Our World From Every Angle
CJASN
Clinical Journal of the American Society of Nephrology

ASN Journal Disclosure Form

As per ASN journal policy, I have disclosed any financial relationships or commitments I have held in the past 36 months as included below. I have listed my Current Employer below to indicate there is a relationship requiring disclosure. If no relationship exists, my Current Employer is not listed.

B. Hendry reports the following:
Employer: Travere Therapeutics; Ownership Interest: Travere Therapeutics; Advisory or Leadership Role: South West Thames Institute for Renal Research (Board Chair);; and Other Interests or Relationships: Emeritus Professor of Medicine, King's College London.

I understand that the information above will be published within the journal article, if accepted, and that failure to comply and/or to accurately and completely report the potential financial conflicts of interest could lead to the following: 1) Prior to publication, article rejection, or 2) Post-publication, sanctions ranging from, but not limited to, issuing a correction, reporting the inaccurate information to the authors' institution, banning authors from submitting work to ASN journals for varying lengths of time, and/or retraction of the published work.

Name: Bruce M. Hendry
Manuscript ID: JASN-2024-001050R1
Manuscript Title: Long term outcomes in nephrotic syndrome: analysis by kidney biopsy diagnosis and proteinuria of UK RaDaR Registry patients
Date of Completion: November 26, 2024
Disclosure Updated Date: May 10, 2024

ASN American Society of Nephrology | JASN Journal of the American Society of Nephrology | KIDNEY360 Accessing Our World From Every Angle | CJASN Clinical Journal of the American Society of Nephrology

ASN Journal Disclosure Form

As per ASN journal policy, I have disclosed any financial relationships or commitments I have held in the past 36 months as included below. I have listed my Current Employer below to indicate there is a relationship requiring disclosure. If no relationship exists, my Current Employer is not listed.

A. Mercer reports the following:
Employer: JAMCO Pharma Consulting AB; Consultancy: Travere Therapeutics, Vera Therapeutics, HiBio/Biogen, Dimerix, Timberlyne Therapeutics, Advanz Pharma; and Ownership Interest: JAMCO Pharma Consulting AB.

I understand that the information above will be published within the journal article, if accepted, and that failure to comply and/or to accurately and completely report the potential financial conflicts of interest could lead to the following: 1) Prior to publication, article rejection, or 2) Post-publication, sanctions ranging from, but not limited to, issuing a correction, reporting the inaccurate information to the authors' institution, banning authors from submitting work to ASN journals for varying lengths of time, and/or retraction of the published work.

Name: Alex Mercer
Manuscript ID: JASN-2024-001050R3
Manuscript Title: Long-Term Outcomes in Nephrotic Syndrome: Analysis by Kidney Biopsy Diagnosis and Proteinuria of UK RaDaR Registry Patients
Date of Completion: April 8, 2025
Disclosure Updated Date: April 8, 2025

ASN American Society of Nephrology
JASN Journal of the American Society of Nephrology
KIDNEY360 Accessing Our World From Every Angle
CJASN Clinical Journal of the American Society of Nephrology

ASN Journal Disclosure Form

As per ASN journal policy, I have disclosed any financial relationships or commitments I have held in the past 36 months as included below. I have listed my Current Employer below to indicate there is a relationship requiring disclosure. If no relationship exists, my Current Employer is not listed.

D. Pitcher reports the following:
Employer: UK Kidney Association; and Research Funding: Current ongoing work as part of employment with UK Kidney Association to analyse data held in the UK National Registry of Rare Kidney Diseases (RaDaR) with Travere Therapeutics, Pfizer, Sanofi, Novartis, Biogen, STADA, Calliditas, Bayer, Takeda.

I understand that the information above will be published within the journal article, if accepted, and that failure to comply and/or to accurately and completely report the potential financial conflicts of interest could lead to the following: 1) Prior to publication, article rejection, or 2) Post-publication, sanctions ranging from, but not limited to, issuing a correction, reporting the inaccurate information to the authors' institution, banning authors from submitting work to ASN journals for varying lengths of time, and/or retraction of the published work.

Name: David Pitcher
Manuscript ID: JASN-2024-001050R1
Manuscript Title: Long term outcomes in nephrotic syndrome: analysis by kidney biopsy diagnosis and proteinuria of UK RaDaR Registry patients
Date of Completion: November 25, 2024
Disclosure Updated Date: November 25, 2024

ASN
American Society of Nephrology
JASN
Journal of the American Society of Nephrology
KIDNEY360
Accessing Our World From Every Angle
CJASN
Clinical Journal of the American Society of Nephrology

ASN Journal Disclosure Form

As per ASN journal policy, I have disclosed any financial relationships or commitments I have held in the past 36 months as included below. I have listed my Current Employer below to indicate there is a relationship requiring disclosure. If no relationship exists, my Current Employer is not listed.

M. Saleem reports the following:
Employer: University of Bristol; Consultancy: Travere Therapeutics; Mission Therapeutics; Pfizer; Confo Therapeutics; Novartis; Santhera; Ownership Interest: Stock options in Purespring Therapeutics; Research Funding: UCB; Evotec; Travere; Honoraria: Purespring Therapeutics - Director and Chief Scientific Officer; Travere Therapeutics;; Patents or Royalties: Purespring Therapeutics; University of Bristol; and Advisory or Leadership Role: Director and Board Member, Purespring Therapeutics; Director, Kidney Research UK.

I understand that the information above will be published within the journal article, if accepted, and that failure to comply and/or to accurately and completely report the potential financial conflicts of interest could lead to the following: 1) Prior to publication, article rejection, or 2) Post-publication, sanctions ranging from, but not limited to, issuing a correction, reporting the inaccurate information to the authors' institution, banning authors from submitting work to ASN journals for varying lengths of time, and/or retraction of the published work.

Name: Moin A. Saleem
Manuscript ID: JASN-2024-001050R1
Manuscript Title: Long term outcomes in nephrotic syndrome: analysis by kidney biopsy diagnosis and proteinuria of UK RaDaR Registry patients
Date of Completion: November 25, 2024
Disclosure Updated Date: November 25, 2024

ASN American Society of Nephrology | JASN Journal of the American Society of Nephrology | KIDNEY360 Accessing Our World From Every Angle | CJASN Clinical Journal of the American Society of Nephrology

ASN Journal Disclosure Form

As per ASN journal policy, I have disclosed any financial relationships or commitments I have held in the past 36 months as included below. I have listed my Current Employer below to indicate there is a relationship requiring disclosure. If no relationship exists, my Current Employer is not listed.

R. Steenkamp reports the following:
Employer: Renal Association, trading UK Kidney Association

I understand that the information above will be published within the journal article, if accepted, and that failure to comply and/or to accurately and completely report the potential financial conflicts of interest could lead to the following: 1) Prior to publication, article rejection, or 2) Post-publication, sanctions ranging from, but not limited to, issuing a correction, reporting the inaccurate information to the authors' institution, banning authors from submitting work to ASN journals for varying lengths of time, and/or retraction of the published work.

Name: Retha Steenkamp
Manuscript ID: JASN-2024-001050R1
Manuscript Title: Long term outcomes in nephrotic syndrome: analysis by kidney biopsy diagnosis and proteinuria of UK RaDaR Registry patients
Date of Completion: December 11, 2024
Disclosure Updated Date: December 11, 2024

ASN American Society of Nephrology | JASN Journal of the American Society of Nephrology | KIDNEY360 Accessing Our World From Every Angle | CJASN Clinical Journal of the American Society of Nephrology

ASN Journal Disclosure Form

As per ASN journal policy, I have disclosed any financial relationships or commitments I have held in the past 36 months as included below. I have listed my Current Employer below to indicate there is a relationship requiring disclosure. If no relationship exists, my Current Employer is not listed.

A. Turner reports the following:
Employer: University of Edinburgh; Consultancy: Purespring 2021-current; Enyo Pharma from 2022; Calliditas Therapeutics from 2023; Speakers Bureau: For staff education, Chiesi 2022; and Other Interests or Relationships: Trustee and supporter of the patient-led charity Alport UK.

I understand that the information above will be published within the journal article, if accepted, and that failure to comply and/or to accurately and completely report the potential financial conflicts of interest could lead to the following: 1) Prior to publication, article rejection, or 2) Post-publication, sanctions ranging from, but not limited to, issuing a correction, reporting the inaccurate information to the authors' institution, banning authors from submitting work to ASN journals for varying lengths of time, and/or retraction of the published work.

Name: A. Neil Turner
Manuscript ID: JASN-2024-001050R1
Manuscript Title: Long term outcomes in nephrotic syndrome: analysis by kidney biopsy diagnosis and proteinuria of UK RaDaR Registry patients
Date of Completion: November 25, 2024
Disclosure Updated Date: November 25, 2024

ASN American Society of Nephrology | JASN Journal of the American Society of Nephrology | KIDNEY360 Accessing Our World From Every Angle | CJASN Clinical Journal of the American Society of Nephrology

ASN Journal Disclosure Form

As per ASN journal policy, I have disclosed any financial relationships or commitments I have held in the past 36 months as included below. I have listed my Current Employer below to indicate there is a relationship requiring disclosure. If no relationship exists, my Current Employer is not listed.

K. Wong reports the following:
Employer: UK Kidney Association

I understand that the information above will be published within the journal article, if accepted, and that failure to comply and/or to accurately and completely report the potential financial conflicts of interest could lead to the following: 1) Prior to publication, article rejection, or 2) Post-publication, sanctions ranging from, but not limited to, issuing a correction, reporting the inaccurate information to the authors' institution, banning authors from submitting work to ASN journals for varying lengths of time, and/or retraction of the published work.

Name: Katie Wong
Manuscript ID: JASN-2024-001050R1
Manuscript Title: Long term outcomes in nephrotic syndrome: analysis by kidney biopsy diagnosis and proteinuria of UK RaDaR Registry patients
Date of Completion: December 16, 2024
Disclosure Updated Date: May 22, 2024
