# Supplementary material for: Long-Term Outcomes in Nephrotic Syndrome by Kidney Biopsy Diagnosis and Proteinuria
Source: J Am Soc Nephrol. 2025 Apr 17;36(7):1398–413. doi: 10.1681/ASN.0000000610 (PMC12187231; doi:10.1681/ASN.0000000610)
Supplement: Supplementary file 2 [file jasn-36-1398-s002.pdf]

Long term outcomes in nephrotic syndrome: analysis by kidney biopsy diagnosis and proteinuria of UK RaDaR Registry patients

Authors: David Pitcher, BSc[1]; Fiona Braddon, BSc[1]; Bruce Hendry, PhD[2]; Alex Mercer, PhD[3]; Jonathan Barratt, PhD[4]; Retha Steenkamp, PhD[1]; Katie Wong, MBBS[1]; A. Neil Turner, PhD[5]; Wu Gong, MS[2]; Daniel P. Gale, PhD[6]; Moin A. Saleem, PhD[7]

Supplemental Materials

Table of Contents

Supplemental Methods 4

Supplemental Results 9

Supplemental Tables 12

Supplemental Table 1. Additional demographic and clinical characteristics at disease onset and clinical outcomes during follow-up for idiopathic nephrotic syndrome patients (INS cohort) 12

Supplemental Table 2 (A) Demographic and clinical characteristics at disease onset and clinical outcomes during follow-up for pediatric idiopathic nephrotic syndrome patients (INS cohort - Pediatrics), including (B) additional demographic and clinical characteristics. 14

Supplemental Table 3. (A) Demographic and clinical characteristics at disease onset and clinical outcomes during follow-up for adult idiopathic nephrotic syndrome patients (INS cohort - Adults), including (B) additional demographic and clinical characteristics. 16

Supplemental Table 4. Demographic and clinical characteristics at disease onset and clinical outcomes during follow-up for incident FSGS-biopsy and MCD-biopsy proteinuria analysis populations: Overall, Pediatrics, Adults 19

Supplemental Table 5. Demographic and clinical characteristics at disease onset and clinical outcomes during follow-up for prevalent FSGS-biopsy and MCD-biopsy proteinuria analysis populations: Overall, Pediatrics, Adults 22

Supplemental Table 6. Clinical outcomes for incident (A) FSGS-biopsy and (B) MCD-biopsy proteinuria analysis populations: complete remission *(*CR*)*, FSGS partial remission *(*FPR*)* and Threshold approaches applying lowest proteinuria value within 6-24 months post-baseline. 25

Supplemental Table 7. Clinical outcomes for prevalent (A) FSGS-biopsy and (B) MCD-biopsy proteinuria analysis populations: complete remission *(*CR*)*, FSGS partial remission *(*FPR*)* and Threshold approaches applying lowest proteinuria value within 6-12 months post-baseline. 26

Supplemental Table 8. Clinical outcomes for prevalent (A) FSGS-biopsy and (B) MCD-biopsy proteinuria analysis populations: complete remission *(*CR*)*, FSGS partial remission *(*FPR*)*and Threshold approaches applying lowest proteinuria value within 6-24 months post-baseline. 29

Supplemental Table 9. Clinical outcomes for incident FSGS-biopsy proteinuria analysis population: Proteinuria response category approach applying lowest proteinuria value within 6-24 months post-baseline 31

Supplemental Table 10. Clinical outcomes for incident FSGS-biopsy proteinuria analysis population: Proteinuria response category approach applying time-averaged proteinuria within 6-12 months post-baseline 32

Supplemental Table 11. Clinical outcomes for incident (A) FSGS-biopsy and (B) MCD-biopsy proteinuria analysis populations: complete remission *(*CR*)*, FSGS partial remission *(*FPR*)* and Threshold approaches applying time-averaged proteinuria within 6-12 months post-baseline.. 33

Supplemental Table 12. Clinical outcomes for prevalent (A) FSGS-biopsy and (B) MCD-biopsy proteinuria analysis populations: complete remission *(*CR*)*, FSGS partial remission *(*FPR*)* and Threshold approaches applying time-averaged proteinuria within 6-12 months post-baseline.. 34

Supplemental Table 13. Clinical outcomes for prevalent (A) FSGS-biopsy and (B) MCD-biopsy proteinuria analysis populations: complete remission *(*CR*)*, FSGS partial remission *(*FPR*)*and Threshold approaches applying time-averaged proteinuria within 6-24 months post-baseline.. 37

Supplemental Table 14. Clinical outcomes for prevalent FSGS-biopsy proteinuria analysis population: Proteinuria response category approach applying lowest proteinuria value within (A) 6-12 months and (B) 6-24 months post-baseline 39

Supplemental Table 15. Clinical outcomes for prevalent FSGS-biopsy proteinuria analysis population: Proteinuria response category approach applying time-averaged proteinuria within (A) 6-12 months and (B) 6-24 months post-baseline.. 41

Supplemental Table 16. Univariable and multivariable analysis of 10-year survival for FSGS-biopsy and MCD-biopsy proteinuria analysis populations 43

Supplemental Table 17. Summary statistics for (A) the number of UPCR measurements per patient during follow-up windows and (B) the number of eGFR measurements per patient used to calculate eGFR slopes. 45

Supplemental Figures 46

Supplemental Figure 1. (A) Summary of the total RaDaR INS population, and (B) eligibility criteria, patient disposition, and study attrition for the incident and prevalent proteinuria analysis populations of FSGS and MCD. 47

Supplemental Figure 2. Venn diagram displaying the degree of overlap between proteinuria analysis populations within the idiopathic nephrotic syndrome (INS) cohort (A) FSGS-biopsy sub-group and (B) MCD-biopsy sub-group.. 49

Supplemental Figure 3. Kaplan-Meier survival curves by age at kidney failure/death for idiopathic nephrotic syndrome patients (A) idiopathic nephrotic syndrome (INS) cohort, (B) INS cohort – Pediatrics, (C) INS cohort – Adults, by diagnosis category.. 50

Supplemental Figure 4. Kaplan-Meier survival curves for incident biopsy populations: (A) FSGS classical remissions, (B) FSGS partial remission, (C) FSGS threshold approach, (D) MCD classical remissions, (E) MCD partial remission, and (F) MCD threshold approaches. All analyses were based on the lowest proteinuria value recorded within 6-24 months post-baseline. 51

Supplemental Figure 5. Kaplan-Meier survival curves for prevalent biopsy populations: (A) FSGS classical remissions, (B) FSGS partial remission, (C) FSGS threshold approach, (D) MCD classical remissions, (E) MCD partial remission, and (F) MCD threshold approaches. All analyses were based on the lowest proteinuria value recorded within 6-12 months post-baseline.. 52

Supplemental Figure 6. Kaplan-Meier survival curves for incident biopsy populations: (A) FSGS classical remissions, (B) FSGS partial remission, (C) FSGS threshold approach, (D) MCD classical remissions, (E) MCD partial remission, and (F) MCD threshold approaches. All analyses were based on the time-averaged proteinuria within 6-12 months post-baseline. 53

Supplemental Figure 7. Kaplan-Meier survival curves for prevalent biopsy populations: (A) FSGS classical remissions, (B) FSGS partial remission, (C) FSGS threshold approach, (D) MCD classical remissions, (E) MCD partial remission, and (F) MCD threshold approaches. All analyses were based on the time-averaged proteinuria within 6-24 months post-baseline. 54

Supplemental Figure 8. Forest plots of prevalent FSGS-biopsy proteinuria analysis population. Percentage change from baseline for lowest proteinuria value (LPV) within 6-12 months post-baseline vs (A) Hazard Ratio for kidney failure/death event and vs (B) eGFR slope over 6 months to 10 years. Percentage change from baseline for time-averaged proteinuria (TAP) within 6-24 months post-baseline vs (C) Hazard Ratio for kidney failure/death event and vs (D)eGFR slope over 6 months to 10 years. 55

Supplemental Figure 9. Forest plots of incident MCD-biopsy proteinuria analysis population. Percentage change from baseline for lowest proteinuria value (LPV) within 6-12 months post-baseline vs (A) Hazard Ratio for kidney failure/death event and vs (B) eGFR slope over 6 months to 10 years. Percentage change from baseline for time-averaged proteinuria (TAP) within 6-24 months post-baseline vs (C) Hazard Ratio for kidney failure/death event and vs (D) eGFR slope over 6 months to 10 years. 56

Supplemental Figure 10. Forest plots of prevalent MCD-biopsy proteinuria analysis population. Percentage change from baseline for lowest proteinuria value (LPV) within 6-12 months post-baseline vs (A) Hazard Ratio for kidney failure/death event and vs (B) eGFR slope over 6 months to 10 years. Percentage change from baseline for time-averaged proteinuria (TAP) within 6-24 months post-baseline vs (C) Hazard Ratio for kidney failure/death event and vs (D) eGFR slope over 6 months to 10 years. 57

Supplemental Figure 11. Kaplan-Meier survival curves of incident FSGS-biopsy proteinuria analysis population: Proteinuria response category approach applying (A) lowest proteinuria value within 6-24 months post-baseline and (B) time-averaged proteinuria within 6-12 months post-baseline 58

Supplemental Figure 12. Kaplan-Meier survival curves of prevalent FSGS-biopsy proteinuria analysis population: Proteinuria response category approach applying (A) lowest proteinuria value within 6-12 months post-baseline and (B) time-averaged proteinuria within 6-24 months post-baseline 59

Supplemental Figure 13. Number of patients contributing data in each calendar year. 60

Supplemental Figure 14. Association between the number of UPCR measurements used in the calculation of proteinuria values and outcomes. 61

References 62

Supplemental Methods

Description of RaDaR

An overall description of the RaDaR registries is given in Wong et al (1). In the current work, data for 4539 patients in the idiopathic nephrotic syndrome (INS) rare disease group (RDG) were extracted from RaDaR on 23rd September 2023. A histogram of the number of patients contributing data in each calendar year is shown below (Supplemental Figure 13). In total, 99 sites have recruited at least one patient to the RaDaR Idiopathic Nephrotic Syndrome RDG, and 54 sites have recruited over 20 patients each. Despite the RDG being named idiopathic NS, some patients actually get a genetic diagnosis for their nephrotic syndrome, but the nomenclature has remained the same.

Mandatory data fields are required to be completed when a patient is recruited, and optional data fields are available to capture information about pathology reports, disease progressions, etc. In addition to these data, some patients have a linkage to historical blood results and medication data supporting longitudinal analysis. Dialysis RaDaR data were enriched with dialysis and transplant data from the UK Renal Registry (UKRR), which captures data for all UK patients and therefore only patients leaving the country were potentially lost during follow-up for kidney failure outcomes. Patients registered for RaDaR have given consent for their past, present, and future clinical data to be used for ongoing and future research into kidney diseases and related conditions.

Analysis of the coverage and representativeness of patients recruited to RaDaR found no systematic recruitment biases[1] and also that patients of Asian ethnicity were over-represented in the idiopathic nephrotic syndrome cohort when compared to the rest of RaDaR.

Data Source

The idiopathic nephrotic syndrome cohort includes Congenital NS (presumed Steroid Resistance); Childhood or adult onset NS with primary Steroid Resistance; Childhood or adult onset NS with late onset Steroid Resistance; Steroid Sensitive NS (full or partial remission in response to

steroids); NS as part of a syndrome e.g. Nail Patella Syndrome and Denys-Drash Syndrome. Ethnicity in RaDaR was based on self-reporting.

Secondary FSGS

Patients with secondary causes of NS (using definitions of KDIGO guidelines) or a primary glomerular disease leading to FSGS were excluded. This exclusion relies on the opinion of expert nephrologists and the baseline characteristics shown in Table 2 and Supplemental Tables 1-3 are consistent with a population of primary and genetic FSGS and minimal change disease. Nevertheless, there may be cases included where a contribution of secondary causes could not be ruled out, particularly in older patients with multiple co-morbidity. For example, a diagnosis of diabetes mellitus would not in itself preclude inclusion, as long as no features of diabetic glomerulosclerosis were present. This is a limitation of the study and parallels the category of FSGS uncertain cause described by KDIGO. Nevertheless, the high range proteinuria present in all the idiopathic nephrotic syndrome cohorts described indicates that the criteria for the idiopathic nephrotic syndrome study appear to have been followed. Moreover, the inclusion criteria for the registry clearly states '**no other causes of NS'** (and there are separate RADAR groups for these), so the likelihood that clinicians and research nurses at the individual sites would misclassify in this way is relatively limited.

Measurements, Assessments and Definitions

Proteinuria status in follow-up was assessed by time-averaged proteinuria (TAP; defined as the time-weighted averages for UPCR[2]) or lowest attained proteinuria value (LPV) (i) over the full duration of follow-up, or (ii) in pre-defined time periods of 6-12 or 6-24 months after baseline, with baseline UPCR used as the reference value when determining relative reduction in proteinuria. Annualized eGFR slopes were calculated using linear regression to fit a straight line through patients' mean eGFR values for each 3-month period of follow-up. Slopes were calculated for total

follow-up from 6-months and 6–30 months after baseline. eGFR was calculated using the CKD epidemiology collaboration (CKD-EPI) 2021 formula[3] in adults and the bedside Schwartz equation for patients <18 years old.[4] UPCR values from spot urine collections were recorded and presented in g/g with urinary albumin-creatinine ratio (UACR) values converted to UPCR using UPCR=UACR/0.7.[5] UPCR and UACR were measured according to accredited and standardized protocols applied at each NHS laboratory at the time of assessment.

Calculation of Time-Averaged Proteinuria

The time-weighted averages for urinary protein-creatinine ratio (UPCR) used to define time-averaged proteinuria were calculated from the area under the curve of serial measurements divided by the length of follow-up. In order to calculate time-averaged proteinuria across a window, it was necessary to extrapolate proteinuria prior to the first observation in the window and after the final observation in the window, a first observation carried back and last observation carried forward approach was applied to form a complete curve. Summary statistics for the number of UPCR measurements per patient available for calculating time-averaged proteinuria are reported in Supplemental Table 17A.

eGFR slope was calculated as an annualized value. From the beginning of the time-period for which the eGFR slope was being calculated, mean averages were calculated for all eGFR values falling in 0-3months, 3-6 months etc. up until the earliest of the end of the specified time-period, and all-cause mortality/kidney failure. These mean averages were used for calculating eGFR slope using linear regression when reporting summary statistics and using linear mixed-models with random slope and intercept when adjusting for other covariates. The eGFR slopes were assumed to be linear; although a more complex model might fit the individual trajectories more closely, assuming a linear relationship allows greater interpretability and comparability with previous

studies.[6] As a sensitivity analysis, eGFR slopes were calculated excluding episodes of AKI. No significant differences in outcomes or associations were observed.

eGFR data for slope analysis was collected from 6 months as there are acute changes in eGFR in the first phase of care due to multiple possible factors (hyperfiltration in NS, AKI recovery phase, fluid management, intensive drug therapy initiation, CV events, infections). The first 6 months of eGFR data therefore carry confounding features. The choice of 30 months as a cut-off was based on the usual period of data available in a clinical trial of 24-30 months. The use of 10 years in the long term analysis was chosen as a compromise between collecting all data and the risk of slopes being inaccurate due to low numbers of datapoints at the longer times. Summary statistics for the number of eGFR measurements per patient are reported in Supplemental Table 17B.

Adjusted hazard ratios for kidney survival were calculated using Cox regression, censoring follow-up at 10-years. Variables were included in the model if there was a significant association found in a univariable analysis, and then backwards selection used to remove non-significant variables in the multivariable analysis.

To assess the association of the number of UPCR measurements used in the calculation of the proteinuria values with outcomes, we have re-run the 6-24 month time-averaged proteinuria analyses in both the incident and prevalent populations stratifying by the median number of UPCR measurements used. The median number of UPCR measurements in each case was 5, so the time to event analyses have been stratified into two groups: those with 1-5 UPCR values and those with 6+ values. (Supplemental Figure 14). Logrank tests found no evidence of significant differences in survival between the strata of patients with the lower number of UPCR datapoints compared to those with a higher number of UPCR datapoints in either the prevalent or incident proteinuria analysis populations for either of the diseases.

Statistical Analyses

Continuous variables were reported as mean (standard deviation [SD]) and median (interquartile range [IQR]), and categorical variables as frequencies and percentages.

Supplemental Results

Description of proteinuria analysis populations

Supplemental Figure 1 provides summaries of eligibility criteria, patient disposition, and study attrition for incident and prevalent proteinuria analysis populations (FSGS and minimal change disease), along with a Venn diagram illustrating the degree of overlap (Supplemental Figure 2). There are 45 patients who have an initial diagnosis of minimal change disease on biopsy and then later have a further biopsy showing FSGS. Patients progressing from minimal change disease to FSGS were included in both minimal change disease -biopsy and FSGS-biopsy populations, and they had one disease onset date defined, rather than separate dates for each disease group. These are 45 of the 1153 patients with minimal change disease (4%) and they represent 45 of the 1303 patient diagnosed with FSGS (3%) (Supplemental Figure 1A). The outcomes for this group examined separately were closer to the full group FSGS outcomes (see Table 2) than to the full-group minimal change disease outcomes. Of these 45 patients, there were 14 and 23 respectively who met the criteria to be included in the incident and prevalent proteinuria analysis populations. These were included in both minimal change disease and FSGS proteinuria analysis populations as they satisfied the diagnostic criteria for both. For the incident proteinuria analysis population these are 14 out of 260 patients in the minimal change disease proteinuria analysis population (5%) and 14 out of 277 patients in the FSGS proteinuria analysis population (5%). For the prevalent proteinuria analysis population these are 23 out of 452 patients in the minimal change disease proteinuria analysis population (5%) and 23 out of 428 patients in the FSGS proteinuria analysis population (5%) (Supplemental Figure 1B). A sensitivity analysis of 10-year survival (95%) in the FSGS cohort excluding these patients found near identical results compared to the results when including them, 0.57 (0.54, 0.60) vs 0.58 (0.55, 0.61) respectively.

Baseline characteristics, follow-up and clinical outcomes for all subpopulations are presented overall in Supplemental Table 1 and stratified by pediatrics and adults in Supplemental Tables 2 & 3 respectively. The percentage of patients with complete data for each variable is displayed in these tables on the grey rows. The incident minimal change disease and FSGS proteinuria analysis populations closely resembled their respective idiopathic nephrotic syndrome cohort subgroups in key parameters indicating that there was no bias in selection. No notable demographic differences emerged between the incident and prevalent populations. For the prevalent populations, selected as populations at high risk of progression (requirement for a proteinuria value ≥1.5 g/g at least 6 months from disease onset), median time (Q1, Q3) from disease onset to baseline was 1.7 (0.6, 6.1) years and 2.1 (0.8, 7.8) years for FSGS and minimal change disease populations, respectively (Supplemental Table 5). Median proteinuria and mean serum albumin at disease onset were comparable between incident and prevalent populations, however, in the prevalent populations, proteinuria was lower and serum albumin was higher at baseline.

CKD stage was the strongest predictor at baseline of 10-year kidney survival in unadjusted and adjusted analyses (Supplemental Table 16). Female sex was also associated with reduced risk of disease progression in both FSGS and minimal change disease subgroups (adjusted hazard ratios [95% CI]: 0.81 [0.67, 0.97] and 0.62 [0.42, 0.91] respectively).

Relationship between proteinuria threshold-based endpoints vs. disease progression and kidney failure in FSGS patients

Table 7B displays the clinical results for the incident FSGS-biopsy proteinuria analysis population following the application of time-averaged proteinuria within 6-24 months post-baseline. Fewer patients achieved these response threshold levels when applying time-averaged proteinuria over 6-24 months (10% achieved <0.75 g/g; 25% achieved <1.5g/g). However, no patients progressed to kidney failure in the <0.75 g/g time-averaged proteinuria group, and for the <1.5 g/g group, the 10-year survival rate (95% CI) was 90% (77-96) with a mean (SD) annual change of eGFR of 0.8

(11.2) mL/min/1.73m^2 (Table 7B). In contrast, the 10-year survival rates (95% CI) and mean (SD) annual changes of eGFR for the time-averaged proteinuria 1.5 to <3.5 g/g and ≥3.5 g/g groups were 58% (46-68) and -4.5 (11.5) mL/min/1.73m^2, and 36% (25-46) and -14.7 (27.1) mL/min/1.73m^2, respectively (Table 7B). Although prevalent patients who achieved time-averaged proteinuria proteinuria response thresholds <1.5 g/g demonstrated good outcomes, the overall 10-year survival estimates (95% CI) were slightly lower compared to the incident population (79% (67-87) vs. 90% (77-96) respectively (Supplemental Table 15B; Table 7B). There was a 100% 10-year kidney survival rate observed in the time-averaged proteinuria 6-24 month <0.5 g/g group (Supplemental Figure 12B; Supplemental Table 15B).

Limitations and Future Perspectives

The retrospective design and reliance on electronic health records may introduce biases. To assess the impact that survivor bias might have had on the study conclusions a sensitivity analysis restricting the prevalent proteinuria analysis to data after the date of recruitment to RaDaR was performed. Similar rates of kidney failure were observed in this sensitivity analysis as the main manuscript, and also the same conclusions of lower proteinuria levels being associated with better survival rates in both the FSGS-biopsy and Minimal Change Disease-biopsy populations. A sensitivity analysis assessing whether patients with greater contact with health services had different outcomes to those with less contact found that the number of UPCR measurements that were used in the determination of proteinuria response group had no association with kidney failure.

Supplemental Tables

Supplemental Table 1. Additional demographic and clinical characteristics at disease onset and clinical outcomes during follow-up for idiopathic nephrotic syndrome patients (INS cohort)

Category	INS-genetic		FSGS-biopsy		MCD-biopsy		INS-no biopsy/genetic diagnosis		MCD progressing to FSGS	
	n	%	n	%	n	%	n	%	n	%
**Age at disease onset**	**105**	**100**	**1303**	**100**	**1153**	**100**	**1550**	**100**	**45**	**100**
**Mean years (SD)**	9.2 (17.8)		35.4 (22.2)		35.3 (23.8)		24.7 (24.0)		23.8 (21.6)	
**BMI at disease onset**	**23**	**22**	**197**	**15**	**135**	**12**	**121**	**8**	**10**	**22**
**Median (Q1, Q3)**	13.9 (9.0 ,17.0)		21.7 (17.8, 29.4)		24.6 (18.2, 30.0)		19.1 (16.5, 22.6)		17.7 (16.0, 20.6)	
**Systolic BP at disease onset**	**19**	**18**	**241**	**19**	**160**	**14**	**135**	**9**	**10**	**22**
**Mean, mmHg (SD)**	100 (17)		133 (24)		127 (21)		116 (18)		120 (19)	
**UPCR at disease onset**	**26**	**25**	**339**	**26**	**325**	**28**	**254**	**16**	**16**	**36**
**Mean, g/g (SD)**	32.6 (33.3)		8.5 (12.3)		7.7 (6.9)		10.2 (13.1)		6.9 (6.4)	
**Serum albumin at disease onset**	**52**	**50**	**529**	**41**	**485**	**42**	**478**	**31**	**20**	**44**
**Mean, g/dl (SD)**	1.9 (1.1)		2.6 (1.0)		2.2 (1.0)		2.6 (1.1)		2.3 (0.9)	
**eGFR at disease onset**	**32**	**31**	**442**	**34**	**377**	**33**	**317**	**21**	**17**	**38**
**Median, ml/min/1.73m^2 (Q1,Q3)**	95 (41, 127)		62 (37, 102)		84 (56,110)		83 (50, 113)		78 (50, 103)	
**Length of follow-up**	**105**	**100**	**1303**	**100**	**1153**	**100**	**1550**	**100**	**45**	**100**
**Mean, years (SD)**	5.7 (5.0)		9.3 (8.7)		11.6 (9.8)		10.3 (8.7)		11.6 (9.1)	
**First event**	**78**	**100**	**638**	**100**	**168**	**100**	**343**	**100**	**18**	**100**
**Death**	1	1	34	5	26	15	41	12	.	.
**Kidney Replacement Therapy**	38	49	269	42	58	35	132	38	10	56
**eGFR <15 ml/min/1.73m^2**	39	50	335	53	84	50	170	50	8	44
**Time to first event**	**78**	**100**	**638**	**100**	**168**	**100**	**343**	**100**	**18**	**100**
**Mean, years (SD)**	4.4 (4.2)		6.2 (7.2)		7.3 (10.3)		7.6 (9.7)		4.8 (5.3)	
**Median, years (Q1, Q3)**	3.1 (1.6, 5.9)		3.7 (1.3, 8.7)		3.9 (0.5, 9.3)		4.2 (1.5, 10.0)		2.2 (2.0, 6.6)	
**Age at first event**	**78**	**100**	**638**	**100**	**168**	**100**	**343**	**100**	**18**	**100**
**Mean, years (SD)**	10.2 (14.8)		42.7 (21.6)		49.4 (27.8)		48.0 (22.3)		28.5 (22.0)	
**Median, years (Q1, Q3)**	3.7 (2.2, 13.2)		45.1 (24.3, 60.5)		59.8 (19.7, 72.6)		51.8 (31.1 ,65.3)		18.9 (9.7, 49.8)	
**Quartile survival estimate, year (95% CI)**	**105**	**100**	**1303**	**100**	**1153**	**100**	**1550**	**100**	**45**	**100**
**75%**	2.1 (1.4, 2.6)		3.6 (2.9, 4.3)		29.3 (20.7, 52.9)		13.6 (12.2, 15.7)		3.8 (2.0, 14.7)	
**50%**	4.5 (3.4, 6.2)		14.1 (12.1, 16.2)		52.9 (50.3, 55.2)		39.4 (31.1, 46.7)		. (10.0, .)	
**25%**	11.5 (8.6, 16.6)		32.7 (29, 44.1)		55.2 (52.9, .)		63 (50.3, .)		. (., .)	

Notes:BMI, body mass index; eGFR, estimated glomerular filtration rate; FSGS, Focal Segmental Glomerulosclerosis; INS, idiopathic nephrotic syndrome patients; MCD, minimal change disease; UPCR, urine protein:creatinine ratio.

Supplemental Table 2 (A) Demographic and clinical characteristics at disease onset and clinical outcomes during follow-up for pediatric idiopathic nephrotic syndrome patients (INS cohort - Pediatrics), including (B) additional demographic and clinical characteristics.

(A)

Category	INS-genetic		FSGS-biopsy		MCD-biopsy		INS-no biopsy/genetic diagnosis		MCD progressing to FSGS	
	n	%	n	%	n	%	n	%	n	%
**Age at disease onset**	**86**	**100**	**356**	**100**	**342**	**100**	**836**	**100**	**21**	**100**
**Median years (Q1, Q3)**	0.1 (0.0, 1.9)		6.1 (3.0, 12.5)		4.8 (2.4, 10.6)		4.2 (2.6, 7.3)		4.4 (3.0, 8.0)	
**Gender**	**86**	**100**	**356**	**100**	**342**	**100**	**836**	**100**	**21**	**100**
**Female**	43	50	173	49	138	40	330	39	10	48
**Ethnicity**	**86**	**100**	**356**	**100**	**342**	**100**	**836**	**100**	**21**	**100**
**Asian**	24	28	55	15	57	17	149	18	1	5
**Black**	5	6	14	4	16	5	34	4	3	14
**Other**	6	7	16	4	14	4	44	5	.	.
**White**	45	52	246	69	216	63	455	54	15	71
**Not stated/missing**	6	7	25	7	39	11	154	18	2	10
**UPCR at disease onset**	**23**	**27**	**91**	**26**	**67**	**20**	**127**	**15**	**8**	**38**
**Median, g/g (Q1, Q3)**	29.9 (13.9, 42.6)		9.6 (4.4, 17.0)		5.4 (2.1, 10.7)		9.2 (4.7, 16.6)		2.3 (0.1, 10.5)	
**Serum albumin at disease onset**	**47**	**55**	**170**	**48**	**113**	**33**	**193**	**23**	**11**	**52**
**Median g/dl (Q1, Q3)**	1.3 (1.0, 2.1)		2.1 (1.6, 3.0)		2.0 (1.4, 2.6)		1.7 (1.2, 2.6)		2.0 (1.5, 3.0)	
**eGFR at disease onset**	**27**	**31**	**97**	**27**	**58**	**17**	**65**	**8**	**7**	**33**
**Mean, ml/min/1.73m^2 (SD)**	100 (64)		108 (43)		128 (39)		130 (52)		105 (23)	
**Length of follow-up**	**86**	**100**	**356**	**100**	**342**	**100**	**836**	**100**	**21**	**100**
**Median, years (Q1, Q3)**	3.8 (1.8, 8.1)		9.7 (3.6, 15.4)		13.4 (8.4, 20.5)		9.3 (6.2, 13.4)		8.6 (3.8, 15.9)	
**Kidney failure or death event**	**86**	**100**	**356**	**100**	**342**	**100**	**836**	**100**	**21**	**100**
**Yes**	68	79	154	43	54	16	72	9	10	48
**Survival rate, estimate (95% CI)**										
**1-year**	0.86 (0.77, 0.92)		0.92 (0.88, 0.94)		0.98 (0.95, 0.99)		0.99 (0.98, 0.99)		0.90 (0.67, 0.98)	
**2.5-year**	0.66 (0.55, 0.75)		0.82 (0.78, 0.86)		0.95 (0.92, 0.97)		0.97 (0.96, 0.98)		0.76 (0.52, 0.89)	
**5-year**	0.44 (0.34, 0.54)		0.74 (0.69, 0.78)		0.92 (0.89, 0.95)		0.95 (0.94, 0.97)		0.67 (0.43, 0.83)	
**10-year**	0.25 (0.16, 0.35)		0.64 (0.58, 0.69)		0.89 (0.85, 0.92)		0.94 (0.92, 0.95)		0.56 (0.33, 0.75)	
**eGFR slope, all follow-up**	**53**	**62**	**211**	**59**	**199**	**58**	**304**	**36**	**16**	**76**
**Mean, ml/min/1.73m^2 (SD)**	-32.7 (37.3)		-12.6 (23.6)		-4.5 (17.0)		-4.1 (21.7)		-7.1 (12.6)	
**Median, ml/min/1.73m^2 (Q1, Q3)**	-20.2 (-46.9, -13.3)		-4.6 (-14.7, -0.4)		-1.8 (-6.0, 0.8)		-2.4 (-8.8, 2.0)		-2.1 (-14.5, -0.1)	
**Time-averaged proteinuria, all follow-up**	**43**	**50**	**267**	**75**	**267**	**78**	**496**	**59**	**19**	**91**
**Mean, g/g (SD)**	24.9 (30.0)		5.8 (8.4)		4.2 (10.8)		3.4 (14.1)		10.5 (11.6)	
**Median, g/g (Q1, Q3)**	11.9 (3.7, 30.6)		2.8 (0.7, 7.4)		1.3 (0.5, 4.1)		1.0 (0.2, 3.1)		6.9 (1.0, 14.7)	

(B)

Category	INS-genetic		FSGS-biopsy		MCD-biopsy		INS-no biopsy/genetic diagnosis		MCD progressing to FSGS	
	n	%	n	%	n	%	n	%	n	%
**Age at disease onset**	**86**	**100**	**356**	**100**	**342**	**100**	**836**	**100**	**21**	**100**
**Mean years (SD)**	1.9 (3.6)		7.7 (5.3)		6.6 (5.1)		5.5 (4.1)		5.5 (3.6)	
**BMI at disease onset**	**23**	**27**	**99**	**28**	**56**	**16**	**86**	**10**	**8**	**38**
**Median (Q1, Q3)**	13.9 (9.0, 17.0)		17.8 (16.3, 19.8)		17.6 (16.1, 20.0)		17.6 (15.1, 20.3)		17.2 (15.8, 18.7)	
**Systolic BP at disease onset**	**18**	**21**	**100**	**28**	**50**	**15**	**82**	**10**	**8**	**38**
**Mean, mmHg (SD)**	98 (13)		116 (19)		111 (16)		110 (14)		114 (16)	
**UPCR at disease onset**	**23**	**27**	**91**	**26**	**67**	**20**	**127**	**15**	**8**	**38**
**Mean, g/g (SD)**	36.0 (33.9)		15.7 (20.9)		8.1 (9.0)		13.2 (14.2)		5.9 (8.0)	
**Serum albumin at disease onset**	**47**	**55**	**170**	**48**	**113**	**33**	**193**	**23**	**11**	**52**
**Mean, g/dl (SD)**	1.8 (1.1)		2.3 (0.9)		2.1 (0.9)		2.0 (1.1)		2.2 (1.1)	
**eGFR at disease onset**	**27**	**31**	**97**	**27**	**58**	**17**	**65**	**8**	**7**	**33**
**Median, ml/min/1.73m^2 (Q1, Q3)**	114 (35, 127)		107 (85, 130)		128 (104, 145)		122 (102, 163)		103 (88, 133)	
**CKD stage at disease onset**	**27**	**31**	**97**	**27**	**58**	**17**	**65**	**8**	**7**	**33**
**Stage 1**	14	52	64	66	46	79	53	82	4	57
**Stage 2**	5	19	18	19	10	17	6	9	3	43
**Stage 3A**	1	4	7	7	1	2	1	2	.	.
**Stage 3B**	2	7	7	7	1	2	2	3	.	.
**Stage 4**	3	11	.	.	.	.	2	3	.	.
**Stage 5**	2	7	1	1	.	.	1	2	.	.
**Length of follow-up**	**86**	**100**	**356**	**100**	**342**	**100**	**836**	**100**	**21**	**100**
**Mean, years (SD)**	5.5 (5.1)		11.5 (10.7)		16.7 (12.5)		11.7 (9.4)		10.2 (7.8)	
**First event**	**68**	**100**	**154**	**100**	**54**	**100**	**72**	**100**	**10**	**100**
**Death**	1	1	5	3	1	2	5	7	.	.
**Kidney Replacement Therapy**	35	51	103	67	39	72	45	63	7	70
**eGFR <15 ml/min/1.73m^2**	32	47	46	30	14	26	22	31	3	30
**Time to first event**	**68**	**100**	**154**	**100**	**54**	**100**	**72**	**100**	**10**	**100**
**Mean, years (SD)**	4.1 (3.9)		6.4 (7.7)		10.3 (13.9)		10.8 (14.3)		4.0 (3.4)	
**Median, years (Q1, Q3)**	2.9 (1.5, 5.5)		3.6 (1.5, 8.5)		5.2 (2.1, 11.3)		4.8 (2.0,13.4)		3.1 (2.0, 6.6)	
**Age at first event**	**68**	**100**	**154**	**100**	**54**	**100**	**72**	**100**	**10**	**100**
**Mean, years (SD)**	5.6 (5.8)		14.5 (8.8)		16.2 (15.3)		17.7 (14.4)		10.8 (5.6)	
**Median, years (Q1, Q3)**	3.2 (1.8, 7.5)		13.7 (7.9, 18.8)		11.7 (8.3, 18.4)		15.2 (6.4, 22.3)		10.4 (5.1, 15.5)	
**Quartile survival estimate, year (95% CI)**										
**75%**	1.8 (1.3, 2.5)		4.6 (2.9, 6.5)		48.1 (24.3, 54.1)		41.1 (31.0, 50.3)		3.8 (0.0, 8.1)	
**50%**	3.8 (2.8, 5.6)		22.3 (15.4, 30.9)		54.1 (52.9, .)		54.3 (44.5, .)		. (3.8, .)	
**25%**	9.6 (6.8, 13.8)		. (43.4, .)		55.2 (52.9, .)		63 (54.3, .)		. (. , .)	

Notes: eGFR, estimated glomerular filtration rate; FSGS, Focal Segmental Glomerulosclerosis; INS, idiopathic nephrotic syndrome patients; MCD, minimal change disease; UPCR, urine protein:creatinine ratio.

Supplemental Table 3. (A) Demographic and clinical characteristics at disease onset and clinical outcomes during follow-up for adult idiopathic nephrotic syndrome patients (INS cohort - Adults), including (B) additional demographic and clinical characteristics.

(A)

Category	INS-genetic		FSGS-biopsy		MCD-biopsy		INS-no biopsy/genetic diagnosis		MCD progressing to FSGS	
	n	%	n	%	n	%	n	%	n	%
**Age at disease onset**	**19**	**100**	**947**	**100**	**811**	**100**	**714**	**100**	**24**	**100**
**Median years (Q1, Q3)**	34.5 (26.1, 58.9)		44.9 (32.2, 57.9)		46.9 (32.1, 61.1)		48.1 (31.7, 60.7)		37.6 (23.5, 53.8)	
**Gender**	**19**	**100**	**947**	**100**	**811**	**100**	**714**	**100**	**24**	**100**
**Female**	10	53	379	40	381	47	309	43	9	38
**Ethnicity**	**19**	**100**	**947**	**100**	**811**	**100**	**714**	**100**	**24**	**100**
**Asian**	2	11	87	9	101	12	43	6	5	21
**Black**	.	.	73	8	23	3	33	5	1	4
**Other**	.	.	23	2	23	3	14	2	1	4
**White**	14	74	674	71	596	73	433	61	16	67
**Not stated/missing**	3	16	90	10	68	8	191	27	1	4
**UPCR at disease onset**	**3**	**16**	**248**	**26**	**258**	**32**	**127**	**18**	**8**	**33**
**Median, g/g (Q1, Q3)**	4.7 (4.6, 10.3)		5.0 (2.7, 7.9)		7.0 (3.8, 10.3)		4.9 (2.4, 9.3)		8.0 (4.1, 10.5)	
**Serum albumin at disease onset**	**5**	**26**	**359**	**38**	**372**	**46**	**285**	**40**	**9**	**38**
**Median g/dl (Q1, Q3)**	2.7 (2.5, 2.8)		2.7 (2.0,3.6)		2.1 (1.5, 3.0)		3.0 (2.1, 3.9)		2.1 (1.9, 2.5)	
**eGFR at disease onset**	**5**	**26**	**345**	**36**	**319**	**39**	**252**	**35**	**10**	**42**
**Mean, ml/min/1.73m^2 (SD)**	73 (43)		61 (34)		76 (32)		72 (33)		58 (20)	
**Length of follow-up**	**19**	**100**	**947**	**100**	**811**	**100**	**714**	**100**	**24**	**100**
**Median, years (Q1, Q3)**	5.7 (4.1, 9.3)		6.6 (2.5, 11.9)		8.0 (4.5, 12.3)		7.7 (3.8, 11.2)		10.6 (4.1, 18.4)	
**Kidney failure or death event**	**19**	**100**	**947**	**100**	**811**	**100**	**714**	**100**	**24**	**100**
**Yes**	10	53	484	51	114	14	271	38	8	33
**Survival rate, estimate (95% CI)**	**19**	**100**	**947**	**100**	**811**	**100**	**714**	**100**	**24**	**100**
**1-year**	0.95 (0.68, 0.99)		0.90 (0.87, 0.91)		0.95 (0.93, 0.96)		0.92 (0.90, 0.94)		1.00 (1.00, 1.00)	
**2.5-year**	0.89 (0.64, 0.97)		0.79 (0.76, 0.82)		0.93 (0.91, 0.95)		0.85 (0.83, 0.88)		0.79 (0.57, 0.91)	
**5-year**	0.71 (0.44, 0.87)		0.68 (0.65, 0.71)		0.91 (0.89, 0.93)		0.78 (0.75, 0.81)		0.75 (0.53, 0.88)	
**10-year**	0.48 (0.21, 0.71)		0.55 (0.52, 0.59)		0.87 (0.84, 0.89)		0.65 (0.61, 0.69)		0.75 (0.53, 0.88)	
**eGFR slope, all follow-up**	**16**	**84**	**684**	**72**	**668**	**82**	**532**	**75**	**22**	**92**
**Mean, ml/min/1.73m^2 (SD)**	-6.1 (7.7)		-4.2 (8.9)		-1.1 (6.8)		-3.3 (9.7)		-5.3 (9.9)	
**Median, ml/min/1.73m^2 (Q1, Q3)**	-3.2 (-8.2, -1.4)		-2.7 (-5.8, -0.6)		-0.6 (-2.2, 0.9)		-1.7 (-4.2, 0.2)		-2.4 (-6.8, -0.2)	
**Time-averaged proteinuria, all follow-up**	**12**	**63**	**545**	**58**	**561**	**69**	**368**	**52**	**20**	**83**
**Mean, g/g (SD)**	3.6 (4.1)		2.5 (2.7)		1.3 (2.1)		1.9 (2.4)		2.2 (2.6)	
**Median, g/g (Q1, Q3)**	1.9 (0.4, 7.5)		1.7 (0.7, 3.3)		0.7 (0.2, 1.5)		0.9 (0.3, 2.8)		1.1 (0.4, 3.2)	

(B)

Category	INS-genetic		FSGS-biopsy		MCD-biopsy		INS-no biopsy/genetic diagnosis		MCD progressing to FSGS	
	n	%	n	%	n	%	n	%	n	%
**Age at disease onset**	**19**	**100**	**947**	**100**	**811**	**100**	**714**	**100**	**24**	**100**
**Mean years (SD)**	42.5 (18.7)		45.8 (16.4)		47.4 (17.3)		47.3 (17.1)		39.8 (17.6)	
**BMI at disease onset**	**0**	**0**	**98**	**10**	**79**	**10**	**35**	**5**	**2**	**8**
**Median (Q1, Q3)**	.		29.3 (25.7, 32.6)		28.4 (24.8, 33.7)		26.2 (22.8, 34.6)		26.0 (20.6, 31.4)	
**Systolic BP at disease onset**	**1**	**5**	**141**	**15**	**110**	**14**	**53**	**7**	**2**	**8**
**Mean, mmHg (SD)**	147 (.)		144 (20)		135 (19)		126 (19)		144 (1)	
**UPCR at disease onset**	**3**	**16**	**248**	**26**	**258**	**32**	**127**	**18**	**8**	**33**
**Mean, g/g (SD)**	6.5 (3.2)		5.8 (4.5)		7.6 (6.2)		7.2 (11.1)		7.9 (4.6)	
**Serum albumin at disease onset**	**5**	**26**	**359**	**38**	**372**	**46**	**285**	**40**	**9**	**38**
**Mean, g/dl (SD)**	2.8 (0.8)		2.8 (0.9)		2.3 (1.0)		3.0 (1.0)		2.3 (0.7)	
**eGFR at disease onset**	**5**	**26**	**345**	**36**	**319**	**39**	**252**	**35**	**10**	**42**
**Median, ml/min/1.73m^2 (Q1, Q3)**	61 (46, 105)		53 (33, 85)		78 (54, 100)		73 (44, 97)		53 (44, 66)	
**CKD stage at disease onset**	**5**	**26**	**345**	**36**	**319**	**39**	**252**	**35**	**10**	**42**
**Stage 1**	2	40	80	23	120	38	79	31	1	10
**Stage 2**	1	20	66	19	98	31	75	30	2	20
**Stage 3A**	1	20	68	20	43	13	31	12	4	40
**Stage 3B**	.	.	64	19	24	8	37	15	3	30
**Stage 4**	1	20	57	17	26	8	24	10	.	.
**Stage 5**	.	.	10	3	8	3	6	2	.	.
**Length of follow-up**	**19**	**100**	**947**	**100**	**811**	**100**	**714**	**100**	**24**	**100**
**Mean, years (SD)**	6.7 (4.3)		8.5 (7.6)		9.4 (7.4)		8.7 (7.6)		12.8 (10.1)	
**First event**	**10**	**100**	**484**	**100**	**114**	**100**	**271**	**100**	**8**	**100**
**Death**	.	.	29	6	25	22	36	13	.	.
**Kidney Replacement Therapy**	3	30	166	34	19	17	87	32	3	38
**eGFR <15 ml/min/1.73m^2**	7	70	289	60	70	61	148	55	5	63
**Time to first event**	**10**	**100**	**484**	**100**	**114**	**100**	**271**	**100**	**8**	**100**
**Mean, years (SD)**	6.4 (5.5)		6.2 (7.1)		5.8 (7.7)		6.8 (7.8)		5.8 (7.1)	
**Median, years (Q1, Q3)**	5.1 (3.3, 9.0)		3.7 (1.3, 8.7)		3.3 (0.1, 7.9)		4.2 (1.3, 9.4)		2.2 (1.9, 8.7)	
**Age at first event**	**10**	**100**	**484**	**100**	**114**	**100**	**271**	**100**	**8**	**100**
**Mean, years (SD)**	41.6 (19.1)		51.7 (16.1)		65.2 (16.0)		56.0 (16.3)		50.6 (11.4)	
**Median, years (Q1, Q3)**	33.9 (29.5, 60.5)		52.1 (39.2, 63.8)		66.7 (57.3, 77.4)		57.6 (45.2, 67.9)		51.1 (39.6, 61.0)	
**Quartile survival estimate, year (95% CI)**	**19**	**100**	**947**	**100**	**811**	**100**	**714**	**100**	**24**	**100**
**75%**	4.5 (0.1, 9.0)		3.2 (2.7, 3.8)		20.4 (18.4, .)		6.2 (4.9, 7.7)		8.7 (1.1, .)	
**50%**	9.0 (4.5, .)		12.1 (10.7, 14.1)		50.3 (., .)		17.5 (14.1, 21.7)		. (14.7, .)	

**25%**	11.0 (9.0, .)	27.3 (24.9, 30.7)	50.3 (., .)	38.2 (30.3, 46.7)	. (19.5, .)

Notes: eGFR, estimated glomerular filtration rate; FSGS, Focal Segmental Glomerulosclerosis; INS, idiopathic nephrotic syndrome patients; MCD, minimal change disease; UPCR, urine protein:creatinine ratio.

Supplemental Table 4. Demographic and clinical characteristics at disease onset and clinical outcomes during follow-up for incident FSGS-biopsy and MCD-biopsy proteinuria analysis populations: Overall, Pediatrics, Adults

	Overall				Pediatric				Adult			
**Category**	FSGS-biopsy		MCD-biopsy		FSGS-biopsy		MCD-biopsy		FSGS-biopsy		MCD-biopsy	
	**n**	**%**	**n**	**%**	**n**	**%**	**n**	**%**	**n**	**%**	**n**	**%**
**Age at disease onset**	**277**	**100**	**260**	**100**	**79**	**100**	**50**	**100**	**198**	**100**	**210**	**100**
**Mean years (SD)**	35.2 (22.4)		41.5 (22.5)		8.0 (4.9)		7.2 (5.1)		46.0 (16.6)		49.6 (16.5)	
**Median years (Q1, Q3)**	35.1 (14.4, 54.4)		46.2 (24.2, 59.5)		7.1 (3.2, 12.7)		6.2 (3.0, 10.4)		44.7(32.4,59.4)		49.4 (36.9, 62.7)	
**Pediatric at disease onset**	79	29	50	19								
**Gender**	**274**	**100**	**260**	**100**	**79**	**100**	**50**	**100**	**198**	**100**	**210**	**100**
**Female**	125	45	115	44	41	52	24	48	84	42	91	43
**Ethnicity**	**274**	**100**	**260**	**100**	**79**	**100**	**50**	**100**	**198**	**100**	**210**	**100**
**Asian**	39	14	38	15	13	16	10	20	26	13	28	13
**Black**	22	8	7	3	5	6	1	2	17	9	6	3
**Other**	5	2	6	2	2	3	1	2	3	2	5	2
**White**	192	69	196	75	58	73	37	74	134	68	159	76
**Not stated/missing**	19	7	13	5	1	1	1	2	18	9	12	6
**UPCR at disease onset**	**231**	**83**	**236**	**91**	**53**	**67**	**42**	**84**	**178**	**90**	**194**	**92**
**Mean, g/g (SD)**	8.5 (12.2)		7.8 (6.3)		16.7 (22.5)		8.8 (7.1)		6.0 (4.1)		7.6 (6.0)	
**Median, g/g (Q1, Q3)**	5.9 (3.5, 9.5)		6.7 (4.3, 10.3)		10.8 (6.6, 17.4)		6.2 (4.1, 12.1)		5.2 (3.2, 7.9)		6.9 (4.5, 10.2)	
**Serum albumin at disease onset**	**228**	**82**	**218**	**84**	**71**	**90**	**46**	**92**	**157**	**79**	**172**	**82**
**Mean, g/dl (SD)**	2.5 (0.9)		2.1 (0.9)		2.1 (0.8)		1.9 (0.9)		2.7 (0.9)		2.1 (0.8)	
**Median g/dl (Q1, Q3)**	2.3 (1.9, 3.3)		2.0 (1.4, 2.6)		2.0 (1.6, 2.5)		1.9 (1.2, 2.4)		2.6 (2.0, 3.5)		2.0 (1.4, 2.6)	
**eGFR at disease onset**	**194**	**70**	**183**	**70**	**43**	**54**	**28**	**56**	**151**	**76**	**155**	**74**
**Mean, ml/min/1.73m^2 (SD)**	78 (39)		85 (35)		112 (40)		129 (29)		69 (33)		78 (30)	
**Median, ml/min/1.73m^2 (Q1, Q3)**	73 (47, 108)		86 (58, 110)		117 (85, 139)		129 (111, 143)		59 (45, 96)		80 (55, 100)	
**Length of follow-up**	**277**	**100**	**260**	**100**	**79**	**100**	**50**	**100**	**198**	**100**	**210**	**100**
**Mean, years (SD)**	6.2 (4.4)		6.6 (3.6)		7.2 (5.0)		8.3 (3.9)		5.8 (4.1)		6.2 (3.3)	
**Median, years (Q1, Q3)**	5.2 (2.4, 9.1)		6.2 (4,8. 9.0)		6.6 (2.3, 11.3)		8.2 (5.1, 11.4)		4.6 (2.4, 8.3)		5.9 (3.7, 8.1)	
**Kidney failure or death event**	**277**	**100**	**260**	**100**	**79**	**100**	**50**	**100**	**198**	**100**	**210**	**100**
**Yes**	106	38	35	14	34	43	12	24	72	36	23	11
**Time to first event**	**106**	**100**	**35**	**100**	**34**	**100**	**12**	**100**	**72**	**100**	**23**	**100**
**Mean, years (SD)**	3.7 (2.9)		5.3 (3.8)		3.2 (2.5)		5.7 (3.0)		3.9 (3.1)		5.0 (4.2)	
**Median, years (Q1, Q3)**	2.5 (1.7, 4.6)		4.3 (2.2, 6.7)		2.3 (1.6, 4.6)		5.2 (3.3, 7.4)		2.7 (1.8, 4.6)		4.1 (2.2, 5.7)	
**Survival rate, estimate (95% CI)**	**277**	**100**	**260**	**100**	**79**	**100**	**50**	**100**	**198**	**100**	**210**	**100**
**1-year**	0.98 (0.95, 0.99)		0.99 (0.97, 1.00)		0.97 (0.90, 0.99)		1.00 (1.00, 1.00)		0.98 (0.95, 0.99)		0.99 (0.96, 1.00)	

**2.5-year**	0.80 (0.75, 0.84)		0.96 (0.93, 0.98)		0.73 (0.62, 0.82)		0.94 (0.82, 0.98)		0.83 (0.77, 0.88)		0.97 (0.93, 0.99)	
**5-year**	0.67 (0.61, 0.73)		0.90 (0.85, 0.93)		0.67 (0.55, 0.76)		0.89 (0.76, 0.95)		0.67 (0.60, 0.74)		0.90 (0.85, 0.94)	
**10-year**	0.55 (0.48, 0.62)		0.82 (0.75, 0.88)		0.56 (0.43, 0.66)		0.76 (0.60, 0.87)		0.53 (0.43, 0.62)		0.84 (0.75, 0.90)	
**eGFR slope, 6-30 months**	**242**	**87**	**231**	**89**	**65**	**82**	**36**	**72**	**177**	**89**	**195**	**95**
**Mean, ml/min/1.73m^2 (SD)**	-8.4 (23.8)		-3.7 (14.0)		-19.0 (38.2)		-8.2 (26.4)		-4.5(13.7)		-2.8(10.1)	
**Median, ml/min/1.73m^2 (Q1, Q3)**	-4.6 (-13.8, 2.0)		-1.0 (-9.1, 3.3)		-13.8 (-36.6, 5.0)		-8.0 (-18.9, 5.8)		-3.8(-10.2,1.6)		-0.7(-6.3,3.0)	
**eGFR slope, total (from 6 months)**	**256**	**92**	**245**	**94**	**70**	**89**	**44**	**88**	**186**	**94**	**201**	**96**
**Mean, ml/min/1.73m^2 (SD)**	-7.7 (20.9)		-3.1 (8.8)		-16.7 (33.5)		-6.9 (14.6)		-4.3(11.8)		-2.3(6.7)	
**Median, ml/min/1.73m^2 (Q1, Q3)**	-3.4 (-9.0, 0.0)		-1.0 (-4.6, 0.5)		-5.3 (-27.4, 1.2)		-4.6 (-13.4, 0.4)		-2.6(-7.1,-0.1)		-0.9(-3.2,0.5)	
**Lowest UPCR value, 6-12 months**	**234**	**85**	**228**	**88**	**60**	**76**	**41**	**82**	**174**	**88**	**187**	**89**
**Mean, g/g (SD)**	2.6 (3.6)		0.9 (2.0)		4.4 (5.8)		2.1 (3.4)		1.9 (2.1)		0.6 (1.4)	
**Median, g/g (Q1, Q3)**	1.4 (0.3, 3.2)		0.1 (0.0 ,0.7)		1.8 (0.4, 6.3)		0.4 (0.1, 2.6)		1.2 (0.3, 2.7)		0.1 (0.0, 0.4)	
**Lowest UPCR value, 6-24 months**	**277**	**100**	**260**	**100**	**79**	**100**	**50**	**100**	**198**	**100**	**210**	**100**
**Mean, g/g (SD)**	2.3 (4.7)		0.8 (2.6)		4.2 (8.0)		2.0 (4.9)		1.5 (1.8)		0.5 (1.4)	
**Median, g/g (Q1, Q3)**	0.9 (0.1, 2.5)		0.1 (0.0 ,0.3)		0.9 (0.1, 4.6)		0.4 (0.1, 2.1)		0.9 (0.2, 2.3)		0.1 (0.0, 0.1)	
**Lowest UPCR value, total**	**277**	**100**	**260**	**100**	**79**	**100**	**50**	**100**	**198**	**100**	**210**	**100**
**Mean, g/g (SD)**	1.8 (4.7)		0.5 (2.4)		3.6 (8.1)		1.4 (4.8)		1.1 (1.7)		0.3 (1.3)	
**Median, g/g (Q1, Q3)**	0.4 (0.1, 1.7)		0.0 (0.0 ,0.1)		0.3 (0.1, 2.3)		0.1 (0.0 ,0.4)		0.4 (0.1, 1.4)		0.0 (0.0, 0.1)	
**Time-averaged proteinuria, 6-12 months**	**234**	**85**	**228**	**88**	**60**	**76**	**41**	**82**	**174**	**88**	**187**	**89**
**Mean, g/g (SD)**	4.7 (5.5)		2.7 (2.4)		8.5 (8.5)		4.4 (3.5)		3.4 (3.1)		2.3 (1.9)	
**Median, g/g (Q1, Q3)**	2.9 (1.7, 5.9)		2.1 (1.1, 3.3)		6.0 (2.4, 12.2)		3.6 (2.1, 6.5)		2.7 (1.6, 4.2)		1.8 (0.9, 2.9)	
**Time-averaged proteinuria, 6-24 months**	**277**	**100**	**260**	**100**	**79**	**100**	**50**	**100**	**198**	**100**	**210**	**100**
**Mean, g/g (SD)**	4.3 (5.2)		2.4 (3.0)		7.4 (7.8)		4.7 (5.1)		3.0 (2.9)		1.9 (1.9)	
**Median, g/g (Q1, Q3)**	2.7 (1.5, 4.9)		1.5 (0.8, 3.1)		4.8 (2.3, 9.3)		3.2 (1.6, 5.7)		2.3 (1.3, 4.0)		1.3 (0.7, 2.5)	
**Time-averaged proteinuria, total**	**277**	**100**	**260**	**100**	**79**	**100**	**50**	**100**	**198**	**100**	**210**	**100**
**Mean, g/g (SD)**	3.8 (5.3)		2.0 (3.6)		6.7 (8.6)		4.6(6.8)		2.6 (2.3)		1.4 (1.7)	
**Median, g/g (Q1, Q3)**	2.2 (1.0, 4.3)		0.9 (0.4, 2.1)		3.7 (1.2, 7.6)		3.0 (0.8, 5.4)		2.1 (1.0, 3.7)		0.8 (0.4, 1.5)	

Notes: eGFR, estimated glomerular filtration rate; FSGS, Focal Segmental Glomerulosclerosis; INS, idiopathic nephrotic syndrome patients; MCD, minimal change disease; UPCR, urine protein:creatinine ratio.

Supplemental Table 5. Demographic and clinical characteristics at disease onset and clinical outcomes during follow-up for prevalent FSGS-biopsy and MCD-biopsy proteinuria analysis populations: Overall, Pediatrics, Adults

	Overall				Pediatric				Adult			
**Category**	FSGS-biopsy		MCD-biopsy		FSGS-biopsy		MCD-biopsy		FSGS-biopsy		MCD-biopsy	
	**n**	**%**	**n**	**%**	**n**	**%**	**n**	**%**	**n**	**%**	**n**	**%**
**Age at baseline**	**428**	**100**	**452**	**100**	**98**	**100**	**91**	**100**	**330**	**100**	**361**	**100**
**Mean years (SD)**	38.1 (21.4)		39.7 (22.0)		9.7 (4.7)		9.3 (4.8)		46.6 (16.5)		47.4 (17.6)	
**Median years (Q1, Q3)**	38.4 (19.1, 55.7)		39.8 (21.2, 57.0)		10.2 (5.5, 13.5)		8.5 (5.4, 13.3)		46.2 (33.2, 59.5)		47.4 (32.2, 60.9)	
**Pediatric at baseline**	98	23	91	20	98	100	91	100				
**Adult at baseline**	330	77	361	80					330	100	361	100
**Time from disease onset to baseline**	**428**	**100**	**452**	**100**	**98**	**100**	**91**	**100**	**330**	**100**	**361**	**100**
**Median, years (Q1, Q3)**	1.7 (0.6, 6.1)		2.1 (0.8, 7.8)		1.1 (0.6, 3.4)		2.2 (0.8, 6.0)		1.9 (0.6, 6.8)		2.0 (0.8, 8.3)	
**Gender**	**428**	**100**	**452**	**100**	**98**	**100**	**91**	**100**	**330**	**100**	**361**	**100**
**Female**	202	47	191	42	51	52	40	44	151	46	151	42
**Ethnicity**	**424**	**100**	**449**	**100**	**98**	**100**	**91**	**100**	**330**	**100**	**361**	**100**
**Asian**	56	13	64	14	16	16	15	16	40	12	49	14
**Black**	20	5	22	5	4	4	7	8	16	5	15	4
**Other**	12	3	17	4	7	7	7	8	5	2	10	3
**White**	313	73	316	70	68	69	55	60	245	74	261	72
**Missing / not stated**	27	6	33	7	3	3	7	8	24	7	26	7
**UPCR at disease onset**	**176**	**41**	**165**	**37**	**32**	**33**	**29**	**32**	**144**	**44**	**136**	**38**
**Mean, g/g (SD)**	7.7 (12.4)		7.5 (7.2)		15.4 (26.6)		8.3 (8.1)		6.0 (4.4)		7.3 (7.0)	
**Median, g/g (Q1, Q3)**	5.6 (3.1, 8.7)		6.6 (3.0, 10.3)		8.4 (4.5, 12.9)		6.4 (2.1, 12.1)		5.2 (2.9, 8.0)		6.6 (3.4, 9.9)	
**UPCR at baseline**	**428**	**100**	**452**	**100**	**98**	**100**	**91**	**100**	**330**	**100**	**361**	**100**
**Mean, g/g (SD)**	4.8 (5.3)		5.7 (5.5)		6.8 (9.1)		7.6 (9.7)		4.2 (3.2)		5.2 (3.6)	
**Median, g/g (Q1, Q3)**	3.3 (2.1, 5.7)		4.3 (2.4, 7.2)		3.7 (2.3, 8.0)		5.1 (2.7, 8.8)		3.3 (2.1, 5.2)		4.2 (2.3, 7.0)	
**Serum albumin at disease onset**	**218**	**51**	**210**	**47**	**67**	**68**	**46**	**51**	**151**	**46**	**164**	**45**
**Mean, g/dl (SD)**	2.6 (0.9)		2.3 (1.0)		2.2 (0.9)		1.9 (0.9)		2.7 (0.9)		2.4 (1.0)	
**Median g/dl (Q1, Q3)**	2.4 (1.9, 3.4)		2.1 (1.5, 3.1)		2.1 (1.6, 1.8)		1.7 (1.2, 2.2)		2.5 (2.0, 3.5)		2.2 (1.6, 3.1)	
**Serum albumin at baseline**	**354**	**83**	**393**	**87**	**85**	**87**	**76**	**84**	**269**	**82**	**317**	**88**
**Mean, g/dl (SD)**	3.1 (0.8)		3.0 (0.9)		2.7 (1.0)		2.6 (0.9)		3.3 (0.8)		3.1 (0.9)	
**Median g/dl (Q1, Q3)**	3.3 (2.5, 3.7)		3.1 (2.4, 3.7)		2.6 (2.1, 3.5)		2.5 (1.9, 3.3)		3.4 (2.8, 3.8)		3.3 (2.5, 3.7)	
**eGFR at disease onset**	**172**	**40**	**161**	**36**	**35**	**36**	**25**	**28**	**137**	**42**	**136**	**38**
**Mean, ml/min/1.73m^2 (SD)**	82 (36)		87 (37)		110 (33)		138 (35)		74 (33)		78 (29)	
**Median, ml/min/1.73m^2 (Q1, Q3)**	81 (50, 109)		84 (62, 110)		110 (94, 131)		133 (113, 148)		66 (48, 101)		79 (55, 96)	
**eGFR at baseline**	**349**	**82**	**391**	**87**	**71**	**72**	**66**	**73**	**278**	**84**	**325**	**90**
**Mean, ml/min/1.73m^2 (SD)**	78 (37)		94 (35)		106 (38)		126 (47)		71 (33)		87 (28)	

**Median, ml/min/1.73m^2 (Q1, Q3)**	76 (47, 106)		90 (72, 116)		108 (80, 132)		122 (89, 163)		68 (41, 99)		88 (68, 110)	
**Length of follow-up from baseline**	**428**	**100**	**452**	**100**	**98**	**100**	**91**	**100**	**330**	**100**	**361**	**100**
**Mean, years (SD)**	6.0 (4.1)		6.3 (3.9)		7.2 (4.2)		7.2 (4.2)		5.7 (4.1)		6.1 (3.8)	
**Median, years (Q1, Q3)**	5.2 (2.8, 8.3)		5.7 (3.5, 8.5)		6.5 (3.9, 10.7)		6.1 (4.2, 10.9)		4.8 (2.6, 7.6)		5.5 (3.3, 8.3)	
**Kidney failure or death event**	**428**	**100**	**452**	**100**	**98**	**100**	**91**	**100**	**330**	**100**	**361**	**100**
**Yes**	150	35	59	13	28	29	17	19	122	37	42	12
**Time to first event from baseline**	**150**	**100**	**59**	**100**	**28**	**100**	**17**	**100**	**122**	**100**	**42**	**100**
**Mean, years (SD)**	4.2 (3.1)		5.3 (3.7)		3.9 (2.7)		4.5 (2.8)		4.2 (3.2)		5.6 (4.0)	
**Median, years (Q1, Q3)**	3.2 (1.9, 5.5)		4.4(2.7,7.0)		3.5 (1.5, 5.7)		4.3 (1.6, 5.8)		3.1 (2.1, 5.5)		4.4 (3.1, 7.7)	
**Survival rate, estimate (95% CI)**	**428**	**100**	**452**	**100**	**98**	**100**	**91**	**100**	**330**	**100**	**361**	**100**
**1-year**	0.97 (0.95, 0.99)		1.00 (0.98, 1.00)		0.98 (0.92, 0.99)		0.98 (0.91, 0.99)		0.97 (0.95, 0.99)		1.00 (1.00, 1.00)	
**2.5-year**	0.86 (0.82, 0.89)		0.97 (0.95, 0.98)		0.88 (0.79, 0.93)		0.94 (0.87, 0.98)		0.85 (0.81, 0.89)		0.98 (0.95, 0.99)	
**5-year**	0.72 (0.67, 0.76)		0.91 (0.87, 0.93)		0.80 (0.70, 0.87)		0.88 (0.78, 0.93)		0.70 (0.64, 0.75)		0.92 (0.88, 0.94)	
**10-year**	0.55 (0.49, 0.61)		0.80 (0.74, 0.85)		0.65 (0.52, 0.75)		0.76 (0.63, 0.85)		0.52 (0.44, 0.59)		0.82 (0.74, 0.87)	
**eGFR slope, 6-30 months**	**356**	**83**	**407**	**90**	**70**	**71**	**71**	**78**	**286**	**87**	**336**	**93**
**Mean, ml/min/1.73m^2 (SD)**	-4.9 (16.2)		-3.3 (14.0)		-9.3 (25.5)		-7.2 (23.6)		-3.8 (12.9)		-2.5 (10.9)	
**Median, ml/min/1.73m^2 (Q1, Q3)**	-3.5 (-10.1, 0.9)		-1.2 (-7.2, 3.4)		-9.0 (-22.7, 3.7)		-4.8 (-18.6, 4.6)		-3.2 (-8.4, 0.6)		-1.0 (-5.8, 3.3)	
**eGFR slope, total (from 6 months)**	**382**	**89**	**437**	**97**	**79**	**81**	**84**	**92**	**303**	**92**	**353**	**98**
**Mean, ml/min/1.73m^2 (SD)**	-5.6 (11.0)		-3.5 (11.1)		-10.0 (18.7)		-8.4 (16.1)		-4.5 (7.5)		-2.4 (9.1)	
**Median, ml/min/1.73m^2 (Q1, Q3)**	-3.3 (-7.4, -0.7)		-1.3 (-4.1, 0.5)		-5.7 (-13.8, -1.3)		-4.2 (-13.9, 0.3)		-2.8 (-6.0, -0.6)		-1.0 (-3.2, 0.5)	
**Lowest UPCR value, 6-12 months**	**364**	**85**	**365**	**81**	**74**	**76**	**68**	**75**	**290**	**88**	**297**	**82**
**Mean, g/g (SD)**	2.3 (2.8)		1.4 (7.7)		3.4 (3.9)		4.7 (17.2)		2.0 (2.4)		0.7 (1.5)	
**Median, g/g (Q1, Q3)**	1.4 (0.3, 3.1)		0.1 (0.0, 0.6)		2.0 (0.3, 4.9)		0.5 (0.1, 2.8)		1.2 (0.3, 2.7)		0.1 (0.0, 0.4)	
**Lowest UPCR value, 6-24 months**	**428**	**100**	**452**	**100**	**98**	**100**	**91**	**100**	**330**	**100**	**361**	**100**
**Mean, g/g (SD)**	1.9 (3.0)		1.1 (6.9)		2.9 (4.5)		3.8 (15.0)		1.7 (2.3)		0.4 (1.2)	
**Median, g/g (Q1, Q3)**	0.9 (0.2, 2.5)		0.1 (0.0, 0.3)		1.0 (0.2, 3.6)		0.2 (0.1, 2.5)		0.9 (0.2, 2.2)		0.1 (0.0, 0.2)	
**Lowest UPCR value, total**	**428**	**100**	**452**	**100**	**98**	**100**	**91**	**100**	**330**	**100**	**361**	**100**
**Mean, g/g (SD)**	1.4 (2.6)		0.8 (6.7)		1.8 (4.2)		2.7 (14.7)		1.2 (1.9)		0.3 (0.9)	
**Median, g/g (Q1, Q3)**	0.4 (0.1, 1.6)		0.0 (0.0, 0.1)		0.3 (0.1, 1.9)		0.1 (0.0, 0.6)		0.5 (0.1, 1.6)		0.0 (0.0, 0.1)	

**Time-averaged proteinuria, 6-12 months**	**364**	**85**	**365**	**81**	**74**	**76**	**68**	**75**	**290**	**88**	**297**	**82**
**Mean, g/g (SD)**	3.4 (3.7)		2.5 (6.2)		5.0 (5.5)		6.1 (13.5)		2.9 (3.0)		1.6 (1.6)	
**Median, g/g (Q1, Q3)**	2.2 (1.3, 4.1)		1.4 (0.5, 2.5)		3.0 (1.6, 6.2)		2.4 (1.3, 5.5)		2.1 (1.2, 3.7)		1.2 (0.5, 2.1)	
**Time-averaged proteinuria, 6-24 months**	**428**	**100**	**452**	**100**	**98**	**100**	**91**	**100**	**330**	**100**	**361**	**100**
**Mean, g/g (SD)**	3.3 (3.8)		2.2 (6.5)		5.0 (5.9)		5.5 (13.7)		2.8 (2.7)		1.4 (1.7)	
**Median, g/g (Q1, Q3)**	2.2 (1.0, 4.2)		1.0 (0.4, 2.3)		3.3 (1.3, 6.1)		2.4 (0.9, 4.8)		2.0 (1.0, 3.8)		0.9 (0.4, 1.8)	
**Time-averaged proteinuria, total**	**428**	**100**	**452**	**100**	**98**	**100**	**91**	**100**	**330**	**100**	**361**	**100**
**Mean, g/g (SD)**	3.1 (3.6)		2.0 (6.4)		4.4 (5.9)		4.9 (13.7)		2.7 (2.4)		1.2 (1.5)	
**Median, g/g (Q1, Q3)**	2.0 (1.0, 3.9)		0.8 (0.3, 1.8)		2.6 (0.9, 5.3)		1.6 (0.8, 4.2)		1.9 (1.0, 3.8)		0.7 (0.3, 1.5)	

Notes: eGFR, estimated glomerular filtration rate; FSGS, Focal Segmental Glomerulosclerosis; INS, idiopathic nephrotic syndrome patients; MCD, minimal change disease; UPCR, urine protein:creatinine ratio.

Supplemental Table 6. Clinical outcomes for incident (A) FSGS-biopsy and (B) MCD-biopsy proteinuria analysis populations: complete remission *(*CR*)*, FSGS partial remission *(*FPR*)* and Threshold approaches applying lowest proteinuria value within 6-24 months post-baseline.

(A)

Incident FSGS-biopsy	n	Kidney survival rate, proportion (95% CI)		Kidney failure risk (10-year), Hazard Ratio (95% Wald CL)		n	eGFR slope, 6-30 months (mL/min/1.73m^2/year)		n	eGFR slope, 6 months to 10 years (mL/min/1.73m^2/year)	
**Lowest proteinuria value, 6-24 months**		**5-year**	**10-year**	**Unadjusted**	**Adjusted**		**Mean (SD)**	**Median (IQR)**		**Mean (SD)**	**Median (IQR)**
**Combined**	277	0.67 (0.61, 0.73)	0.55 (0.48, 0.62)	N/A	N/A	242	-8.4 (23.8)	-4.6 (-13.8, 2.0)	256	-7.7 (20.9)	-3.5 (-9.2, 0.2)
**Classical**											
**CR**	86	0.93 (0.85, 0.97)	0.87 (0.75, 0.94)	0.09 (0.04, 0.20)	0.08 (0.03, 0.17)	79	-3.3 (18.6)	-0.5 (-9.9, 3.0)	82	-2.3 (15.8)	-1.3 (-4.1, 1.7)
**PR**	105	0.71 (0.61, 0.80)	0.56 (0.43, 0.67)	0.38 (0.25, 0.59)	0.42 (0.26, 0.66)	93	-5.5 (19.3)	-3.8 (-10.2, 3.6)	99	-4.6 (14.7)	-2.9 (-7.4, 0.4)
**NR**	86	0.39 (0.28, 0.49)	0.26 (0.16, 0.37)	Ref	Ref	70	-18.1 (30.8)	-10.7 (-27.2, -2.3)	75	-17.5 (28.5)	-9.7 (-22.9, -3.4)
**FPR**											
**CR**	86	0.93 (0.85, 0.97)	0.87 (0.75, 0.94)	0.11 (0.05, 0.23)	0.09 (0.04, 0.20)	79	-3.3 (18.6)	-0.5 (-9.9, 3.0)	82	-2.3 (15.8)	-1.3 (-4.1, 1.7)
**FPR**	77	0.74 (0.61, 0.83)	0.63 (0.47, 0.75)	0.38 (0.23, 0.61)	0.42 (0.25, 0.72)	71	-5.7 (20.4)	-4.5 (-10.2, 3.3)	74	-4.2 (16.0)	-2.1 (-7.2, 0.6)
**N-FPR**	114	0.46 (0.36, 0.55)	0.30 (0.21, 0.40)	Ref	Ref	92	-15.0 (28.4)	-8.0 (-21.6, -1.3)	100	-14.6 (25.6)	-7.7 (-17.5, -2.5)
**Threshold**											
**<0.3 g/g**	86	0.93 (0.85, 0.97)	0.87 (0.75, 0.94)	0.08 (0.04, 0.17)	0.05 (0.02, 0.13)	79	-3.3 (18.6)	-0.5 (-9.9, 3.0)	82	-2.3 (15.8)	-1.3 (-4.1, 1.7)
**0.3 to <1.5 g/g**	91	0.70 (0.59, 0.79)	0.56 (0.41, 0.69)	0.31 (0.19, 0.51)	0.24 (0.13, 0.43)	82	-5.7 (19.4)	-4.7 (-9.4, 2.8)	87	-4.3 (14.9)	-2.2 (-7.3, 0.4)
**1.5 to <3.5 g/g**	52	0.53 (0.38, 0.66)	0.34 (0.20, 0.48)	0.59 (0.36, 0.96)	0.44 (0.26, 0.77)	38	-7.2 (18.3)	-5.6 (-14.8, 3.0)	44	-7.3 (14.0)	-6.1 (-12.6, -2.0)
**≥3.5 g/g**	48	0.35 (0.22, 0.48)	0.25 (0.12, 0.39)	Ref	Ref	43	-24.2 (35.4)	-15.3 (-36.6, -4.7)	43	-25.0 (33.7)	-15.3 (-30.6, -3.4)

(B)

Incident MCD-biopsy<br><br>Lowest proteinuria value, 6-24 months	n	Kidney survival rate, proportion (95% CI)		Kidney failure risk (10-year), Hazard Ratio (95% Wald CL)		n	eGFR slope, 6-30 months (mL/min/1.73m^2/year)		n	eGFR slope, 6 months to 10 years (mL/min/1.73m^2/year)	
		5-year	10-year	Unadjusted	Adjusted		Mean (SD)	Median (IQR)		Mean (SD)	Median (IQR)
**Combined**	228	0.90 (0.85, 0.93)	0.83 (0.74, 0.88)	N/A	N/A	231	-3.7 (14.0)	-1.0 (-9.1, 3.3)	244	-3.0 (8.7)	-1.0 (-4.6, 0.6)
**Classical**											
**CR**	195	0.94 (0.88, 0.96)	0.88 (0.79, 0.93)	0.20 (0.08, 0.48)	0.19 (0.07, 0.55)	178	-2.1 (10.3)	0.0 (-6.2, 3.5)	186	-2.3 (6.7)	-0.9 (-3.4, 0.5)
**PR**	43	0.80 (0.62, 0.90)	0.70 (0.49, 0.84)	0.64 (0.24, 1.65)	0.58 (0.20, 1.66)	35	-9.0 (20.7)	-7.1 (-15.8, -0.2)	39	-4.2 (12.8)	-1.6 (-9.9, 0.7)
**NR**	22	0.77 (0.53, 0.90)	0.62 (0.38, 0.79)	Ref	Ref	18	-8.6 (24.6)	-4.5 (-27.5, 4.8)	19	-7.9 (13.2)	-2.7 (-15.3, 3.0)
**FPR**											
**CR**	195	0.94 (0.88, 0.96)	0.88 (0.79, 0.93)	0.21 (0.09, 0.48)	0.19 (0.07, 0.52)	178	-2.1 (10.3)	0.0 (-6.2, 3.5)	186	-2.3 (6.7)	-0.9 (-3.4, 0.5)
**FPR**	34	0.79 (0.58, 0.90)	0.72 (0.48, 0.86)	0.63 (0.24, 1.66)	0.51 (0.17, 1.55)	28	-10.1 (17.9)	-6.7 (-13.9, -1.6)	31	-5.1 (8.2)	-2.9 (-6.9, 0.2)
**N-FPR**	31	0.80 (0.60, 0.90)	0.62 (0.41, 0.78)	Ref	Ref	25	-7.5 (25.9)	-5.0 (-25.7, 4.8)	27	-5.8 (17.1)	-2.6 (-13.0, 3.0)
**Threshold**											
**<0.3 g/g**	195	0.94 (0.88, 0.96)	0.88 (0.79, 0.93)	0.14 (0.06, 0.35)	0.16 (0.05, 0.45)	178	-2.1 (10.3)	0.0 (-6.2, 3.5)	186	-2.3 (6.7)	-0.9 (-3.4, 0.5)
**0.3 to <1.5 g/g**	39	0.82 (0.63, 0.91)	0.71 (0.48, 0.85)	0.41 (0.15, 1.15)	0.44 (0.14, 1.36)	33	-9.6 (17.4)	-6.2 (-15.0, -0.2)	36	-4.4 (8.0)	-1.6 (-6.3, 0.6)
**1.5 to <3.5 g/g**	11	0.90 (0.47, 0.99)	0.77 (0.34, 0.94)	0.36 (0.07, 1.74)	0.44 (0.07, 2.76)	8	-7.1 (31.2)	-6.1 (-34.7, 5.8)	9	-0.3 (22.8)	1.4 (-7.7, 5.4)
**≥3.5 g/g**	15	0.65 (0.36, 0.84)	0.51 (0.24, 0.73)	Ref	Ref	12	-8.2 (27.5)	-5.4 (-21.4, 4.0)	13	-11.7 (14.2)	-5.8 (-25.1, -2.4)

Notes: CR, complete remission; eGFR, estimated glomerular filtration rate; FPR, FSGS partial remission; FSGS, Focal Segmental Glomerulosclerosis; MCD, minimal change disease; NR, no remission; PR, partial remission.

Supplemental Table 7. Clinical outcomes for prevalent (A) FSGS-biopsy and (B) MCD-biopsy proteinuria analysis populations: complete remission *(*CR*)*, FSGS partial remission *(*FPR*)* and Threshold approaches applying lowest proteinuria value within 6-12 months post-baseline.

(A)

**Prevalent FSGS-biopsy**	**n**	**Kidney survival rate, proportion (95% CI)**		**Kidney failure risk (10-year), Hazard Ratio (95% Wald CL)**		**n**	**eGFR slope, 6-30 months (mL/min/1.73m^2/year)**		**n**	**eGFR slope, 6 months to 10 years (mL/min/1.73m^2/year)**	
**Lowest proteinuria value, 6-12 months**		**5-year**	**10-year**	**Unadjusted**	**Adjusted**		**Mean (SD)**	**Median (IQR)**		**Mean (SD)**	**Median (IQR)**
**Combined**	364	0.70 (0.65, 0.75)	0.54 (0.47, 0.61)	N/A	N/A	308	-5.1 (16.6)	-3.7 (-10.8, 0.6)	324	-5.7 (11.0)	-3.3 (-8.4, -0.6)
**Classical**											
**CR**	92	0.95 (0.86, 0.98)	0.82 (0.65, 0.91)	0.14 (0.07, 0.28)	0.13 (0.06, 0.29)	81	-2.1 (9.7)	-1.4 (-4.5, 1.7)	84	-1.8 (4.9)	-1.1 (-2.9, 0.5)
**PR**	102	0.69 (0.58, 0.78)	0.58 (0.45, 0.69)	0.60 (0.40, 0.91)	0.49 (0.31, 0.76)	85	-4.3 (19.5)	-4.5 (-10.0, 0.3)	91	-5.2 (9.6)	-3.8 (-7.9, -0.6)
**NR**	170	0.58 (0.50, 0.66)	0.38 (0.29, 0.47)	Ref	Ref	142	-7.4 (17.6)	-5.5 (-14.0, 0.0)	149	-8.3 (13.4)	-5.0 (-12.0, -1.6)
**FPR**											
**CR**	92	0.95 (0.86, 0.98)	0.82 (0.65, 0.91)	0.13 (0.07, 0.28)	0.15 (0.07, 0.32)	81	-2.1 (9.7)	-1.4 (-4.5, 1.7)	84	-1.8 (4.9)	-1.1 (-2.9, 0.5)
**FPR**	65	0.83 (0.70, 0.90)	0.66 (0.47, 0.79)	0.40 (0.23, 0.69)	0.47 (0.27, 0.82)	54	-2.8 (22.7)	-4.2 (-10.0, 1.5)	59	-4.5 (10.1)	-3.4 (-7.1, -0.3)
**N-FPR**	207	0.56 (0.49, 0.63)	0.39 (0.31, 0.48)	Ref	Ref	173	-7.3 (16.7)	-5.2 (-13.1, 0.0)	181	-8.0 (12.7)	-4.9 (-11.2, -1.6)
**Threshold**											
**<0.3 g/g**	92	0.95 (0.86, 0.98)	0.82 (0.65, 0.91)	0.10 (0.05, 0.22)	0.09 (0.04, 0.20)	81	-2.1 (9.7)	-1.4 (-4.5, 1.7)	84	-1.8 (4.9)	-1.1 (-2.9, 0.5)
**0.3 to <1.5 g/g**	101	0.78 (0.67, 0.85)	0.59 (0.44, 0.71)	0.37 (0.23, 0.59)	0.32 (0.19, 0.53)	86	-3.3 (18.9)	-3.7 (-8.9, 0.3)	93	-4.3 (8.6)	-3.3 (-7.1, -0.6)
**1.5 to <3.5 g/g**	89	0.57 (0.45, 0.67)	0.45 (0.31, 0.57)	0.69 (0.45, 1.04)	0.45 (0.28, 0.72)	74	-2.9 (14.0)	-3.7 (-9.3, 0.8)	77	-5.0 (7.8)	-4.2 (-8.4, -1.3)
**≥3.5 g/g**	82	0.50 (0.38, 0.60)	0.31 (0.20, 0.43)	Ref	Ref	67	-13.7 (19.8)	-11.0 (-21.5, -3.2)	70	-13.2 (17.1)	-9.2 (-17.6, -3.7)

(B)

Prevalent MCD-biopsy<br><br>Lowest proteinuria value, 6-12 months	n	Kidney survival rate, proportion (95% CI)		Kidney failure risk (10-year), Hazard Ratio (95% Wald CL)		n	eGFR slope, 6-30 months (mL/min/1.73m^2/year)		n	eGFR slope, 6 months to 10 years (mL/min/1.73m^2/year)	
		5-year	10-year	Unadjusted	Adjusted		Mean (SD)	Median (IQR)		Mean (SD)	Median (IQR)
**Combined**	365	0.89 (0.85, 0.92)	0.79 (0.72, 0.85)	N/A	N/A	333	-3.8 (14.4)	-1.1 (-7.6, 3.1)	353	-3.9 (11.9)	-1.2 (-4.6, 0.6)
**Classical**											
**CR**	240	0.95 (0.91, 0.97)	0.87 (0.78, 0.92)	0.23 (0.12, 0.45)	0.23 (0.11, 0.49)	223	-2.2 (10.4)	-0.8 (-5.5, 2.9)	233	-1.8 (7.6)	-0.9 (-3.6, 0.7)
**PR**	64	0.84 (0.71, 0.92)	0.76 (0.60, 0.86)	0.59 (0.28, 1.24)	0.45 (0.20, 1.02)	57	-7.7 (19.6)	-1.1 (-13.5, 2.8)	61	-7.8 (18.1)	-1.3 (-7.2, 0.7)
**NR**	61	0.73 (0.59, 0.83)	0.60 (0.43, 0.73)	Ref	Ref	53	-6.4 (20.1)	-4.7 (-14.2, 3.7)	59	-8.0 (15.6)	-4.7 (-11.7, 0.2)
**FPR**											
**CR**	240	0.95 (0.91, 0.97)	0.87 (0.78, 0.92)	0.24 (0.13, 0.47)	0.28 (0.14, 0.58)	223	-2.2 (10.4)	-0.8 (-5.5, 2.9)	233	-1.8 (7.6)	-0.9 (-3.6, 0.7)
**FPR**	53	0.83 (0.67, 0.92)	0.76 (0.58, 0.87)	0.63 (0.29, 1.40)	0.59 (0.25, 1.40)	47	-7.2 (16.9)	-0.9 (-13.5, 2.7)	51	-7.3 (15.6)	-1.6 (-7.2, 0.5)
**N-FPR**	72	0.75 (0.63, 0.85)	0.62 (0.46, 0.74)	Ref	Ref	63	-7.1 (21.8)	-4.7 (-14.8, 4.5)	69	-8.4 (17.9)	-4.1 (-11.4, 0.4)
**Threshold**											
**<0.3 g/g**	240	0.95 (0.91, 0.97)	0.87 (0.78, 0.92)	0.16 (0.07, 0.34)	0.13 (0.06, 0.32)	223	-2.2 (10.4)	-0.8 (-5.5, 2.9)	233	-1.8 (7.6)	-0.9 (-3.6, 0.7)
**0.3 to <1.5 g/g**	65	0.82 (0.68, 0.90)	0.70 (0.50, 0.83)	0.44 (0.20, 1.01)	0.31 (0.13, 0.76)	57	-6.8 (16.1)	-1.1 (-13.5, 2.7)	62	-6.9 (15.2)	-1.4 (-7.3, 0.9)
**1.5 to <3.5 g/g**	32	0.88 (0.68, 0.96)	0.70 (0.43, 0.86)	0.39 (0.14, 1.04)	0.23 (0.08, 0.68)	27	-6.0 (19.4)	-2.5 (-11.6, 5.7)	30	-6.9 (17.3)	-3.7 (-7.2, -0.3)
**≥3.5 g/g**	28	0.61 (0.39, 0.77)	0.55 (0.32, 0.73)	Ref	Ref	26	-8.9 (27.1)	-7.2 (-25.0, 4.2)	28	-11.1 (19.9)	-5.0 (-16.3, 0.3)

Notes: CR, complete remission; eGFR, estimated glomerular filtration rate; FPR, FSGS partial remission; FSGS, Focal Segmental Glomerulosclerosis; MCD, minimal change disease; NR, no remission; PR, partial remission.

Supplemental Table 8. Clinical outcomes for prevalent (A) FSGS-biopsy and (B) MCD-biopsy proteinuria analysis populations: complete remission *(*CR*)*, FSGS partial remission *(*FPR*)*and Threshold approaches applying lowest proteinuria value within 6-24 months post-baseline.

(A)

Prevalent FSGS-biopsy<br><br>Lowest proteinuria value, 6-24 months	n	Kidney survival rate, proportion (95% CI)		Kidney failure risk (10-year), Hazard Ratio (95% Wald CL)		n	eGFR slope, 6-30 months (mL/min/1.73m^2/year)		n	eGFR slope, 6 months to 10 years (mL/min/1.73m^2/year)	
		5-year	10-year	Unadjusted	Adjusted		Mean (SD)	Median (IQR)		Mean (SD)	Median (IQR)
**Combined**	428	0.72 (0.67, 0.76)	0.55 (0.49, 0.61)	N/A	N/A	356	-4.9 (16.2)	-3.5 (-10.1, 0.9)	379	-5.6 (11.1)	-3.3 (-7.9, -0.6)
**Classical**											
**CR**	136	0.94 (0.87, 0.97)	0.82 (0.70, 0.89)	0.15 (0.08, 0.26)	0.14 (0.07, 0.25)	117	-1.9 (11.6)	-1.4 (-5.6, 2.1)	124	-1.7 (5.9)	-1.1 (-3.3, 0.6)
**PR**	143	0.70 (0.61, 0.77)	0.51 (0.39, 0.61)	0.61 (0.43, 0.87)	0.58 (0.40, 0.84)	122	-3.4 (18.7)	-3.2 (-9.1, 1.0)	131	-5.1 (9.8)	-3.9 (-7.7, -0.8)
**NR**	149	0.55 (0.47, 0.63)	0.38 (0.28, 0.48)	Ref	Ref	117	-9.6 (16.6)	-6.7 (-14.6, -1.8)	124	-10.1 (14.4)	-6.1 (-12.9, -2.4)
**FPR**											
**CR**	136	0.94 (0.87, 0.97)	0.82 (0.70, 0.89)	0.15 (0.09, 0.27)	0.14 (0.08, 0.26)	117	-1.9 (11.6)	-1.4 (-5.6, 2.1)	124	-1.7 (5.9)	-1.1 (-3.3, 0.6)
**FPR**	87	0.77 (0.65, 0.85)	0.60 (0.44, 0.72)	0.46 (0.30, 0.72)	0.47 (0.30, 0.74)	74	-1.6 (21.5)	-2.0 (-9.1, 4.2)	80	-3.8 (10.6)	-2.8 (-5.9, 0.2)
**N-FPR**	205	0.56 (0.49, 0.63)	0.38 (0.29, 0.46)	Ref	Ref	165	-8.6 (15.7)	-6.1 (-13.7, -1.4)	175	-9.2 (13.0)	-5.5 (-12.0, -2.4)
**Threshold**											
**<0.3 g/g**	136	0.94 (0.87, 0.97)	0.82 (0.70, 0.89)	0.13 (0.07, 0.23)	0.12 (0.06, 0.22)	117	-1.9 (11.6)	-1.4 (-5.6, 2.1)	124	-1.7 (5.9)	-1.1 (-3.3, 0.6)
**0.3 to <1.5 g/g**	126	0.73 (0.63, 0.80)	0.55 (0.43, 0.66)	0.44 (0.28, 0.68)	0.42 (0.26, 0.68)	109	-3.2 (18.4)	-3.1 (-9.1, 1.0)	117	-4.3 (9.1)	-3.5 (-7.0, -0.8)
**1.5 to <3.5 g/g**	95	0.57 (0.46, 0.67)	0.36 (0.25, 0.48)	0.80 (0.53, 1.22)	0.70 (0.44, 1.11)	74	-4.9 (13.1)	-4.9 (-11.2, -0.1)	79	-6.6 (7.9)	-4.9 (-10.2, -2.4)
**≥3.5 g/g**	71	0.51 (0.39, 0.62)	0.36 (0.23, 0.50)	Ref	Ref	56	-14.8 (20.1)	-10.9 (-23.2, -2.9)	59	-15.0 (18.9)	-9.7 (-22.0, -4.4)

(B)

Prevalent MCD-biopsy<br><br>Lowest proteinuria value, 6-24 months	n	Kidney survival rate, proportion (95% CI)		Kidney failure risk (10-year), Hazard Ratio (95% Wald CL)		n	eGFR slope, 6-30 months (mL/min/1.73m²/year)		n	eGFR slope, 6 months to 10 years (mL/min/1.73m²/year)	
		5-year	10-year	Unadjusted	Adjusted		Mean (SD)	Median (IQR)		Mean (SD)	Median (IQR)
**Combined**	452	0.91 (0.87, 0.93)	0.80 (0.74, 0.85)	N/A	N/A	407	-3.3 (14.0)	-1.2 (-7.2, 3.4)	436	-3.5 (11.1)	-1.3 (-4.3, 0.6)
**Classical**											
**CR**	336	0.96 (0.93, 0.98)	0.90 (0.84, 0.94)	0.17 (0.09, 0.32)	0.15 (0.07, 0.31)	312	-2.6 (10.8)	-1.2 (-6.1, 2.8)	327	-1.8 (8.2)	-1.0 (-3.2, 0.7)
**PR**	61	0.80 (0.66, 0.88)	0.58 (0.41, 0.73)	0.88 (0.45, 1.71)	0.67 (0.32, 1.42)	52	-5.8 (23.7)	-1.1 (-12.6, 6.1)	58	-8.0 (17.6)	-3.0 (-7.3, 0.1)
**NR**	55	0.72 (0.57, 0.83)	0.59 (0.42, 0.73)	Ref	Ref	43	-5.6 (18.4)	-3.7 (-14.8, 3.7)	51	-9.6 (14.3)	-4.7 (-17.2, 0.4)
**FPR**											
**CR**	336	0.96 (0.93, 0.98)	0.90 (0.84, 0.94)	0.17 (0.09, 0.32)	0.14 (0.07, 0.29)	312	-2.6 (10.8)	-1.2 (-6.1, 2.8)	327	-1.8 (8.2)	-1.0 (-3.2, 0.7)
**FPR**	53	0.79 (0.64, 0.88)	0.58 (0.39, 0.73)	0.89 (0.46, 1.73)	0.61 (0.29, 1.29)	46	-3.9 (21.7)	-0.9 (-10.6, 7.1)	51	-6.8 (14.6)	-3.1 (-7.3, 0.1)
**N-FPR**	63	0.74 (0.60, 0.84)	0.59 (0.42, 0.72)	Ref	Ref	49	-7.5 (21.1)	-4.7 (-14.8, 3.7)	58	-10.4 (17.3)	-4.7 (-17.2, 0.4)
**Threshold**											
**<0.3 g/g**	336	0.96 (0.93, 0.98)	0.90 (0.84, 0.94)	0.15 (0.07, 0.32)	0.09 (0.04, 0.22)	312	-2.6 (10.8)	-1.2 (-6.1, 2.8)	327	-1.8 (8.2)	-1.0 (-3.2, 0.7)
**0.3 to <1.5 g/g**	63	0.75 (0.61, 0.85)	0.57 (0.40, 0.71)	0.84 (0.39, 1.80)	0.41 (0.17, 1.00)	55	-3.6 (20.7)	-1.0 (-12.4, 6.6)	60	-6.9 (14.3)	-3.0 (-7.3, 0.4)
**1.5 to <3.5 g/g**	26	0.91 (0.68, 0.98)	0.60 (0.29, 0.81)	0.62 (0.22, 1.71)	0.41 (0.13, 1.30)	17	-7.6 (26.2)	-5.0 (-13.7, 1.3)	22	-9.8 (19.5)	-4.6 (-12.7, -0.1)
**≥3.5 g/g**	27	0.65 (0.44, 0.80)	0.59 (0.37, 0.76)	Ref	Ref	23	-9.4 (19.2)	-3.7 (-20.9, 3.7)	27	-12.1 (16.8)	-4.8 (-20.9, 0.2)

Notes: CR, complete remission; eGFR, estimated glomerular filtration rate; FPR, FSGS partial remission; FSGS, Focal Segmental Glomerulosclerosis; MCD, minimal change disease; NR, no remission; PR, partial remission.

Supplemental Table 9. Clinical outcomes for incident FSGS-biopsy proteinuria analysis population: Proteinuria response category approach applying lowest proteinuria value within 6-24 months post-baseline

	n (%)	Proteinuria at disease onset (g/g)	Kidney failure or death event	Kidney survival rate, proportion (95% CI)		Kidney failure risk (10-year), Hazard Ratio (95% Wald CL)		n	eGFR slope, 6 months to 10 years (mL/min/1.73m²/year)	
**Lowest proteinuria value, 6-24 months**		**Median (Q1, Q3)**	**n (%)**	**5-year**	**10-year**	**Unadjusted**	**Adjusted**		**Mean (SD)**	**Median (IQR)**
**Combined**	277 (100)	6.1 (3.6, 10.0)	101 (36)	0.67 (0.61, 0.73)	0.55 (0.48, 0.62)	NA	NA	259	-7.1 (20.0)	-3.1 (-9.2, 0.1)
**<0.3 g/g**	86 (31)	7.4 (4.5, 10.3)	8 (9)	0.93 (0.85, 0.97)	0.87 (0.75, 0.94)	0.08 (0.04, 0.17)	0.07 (0.03, 0.15)	82	-1.3 (12.5)	-1.3 (-4.1, 1.7)
**<0.5 g/g**	108 (39)	7.2 (4.4, 10.6)	15 (14)	0.87 (0.78, 0.92)	0.82 (0.71, 0.89)	0.12 (0.07, 0.22)	0.11 (0.06, 0.21)	104	-1.0 (12.7)	-1.3 (-4.2, 2.0)
**<0.75 g/g**	127 (46)	7.0 (3.8, 10.6	20 (16)	0.85 (0.76, 0.90)	0.80 (0.71, 0.87)	0.14 (0.08, 0.25)	0.13 (0.08, 0.24)	121	-1.6 (12.8)	-1.5 (-4.8, 1.7)
**<1.0 g/g**	148 (53)	6.5 (3.7, 10.1)	27 (18)	0.83 (0.75, 0.89)	0.77 (0.67, 0.84)	0.17 (0.10, 0.27)	0.16 (0.09, 0.26)	142	-2.8 (15.0)	-1.6 (-5.0, 1.4)
**<1.5 g/g**	177 (64)	6.1 (3.6, 9.4)	37 (21)	0.81 (0.74, 0.87)	0.71 (0.61, 0.79)	0.19 (0.12, 0.30)	0.18 (0.11, 0.29)	171	-3.0 (13.8)	-1.9 (-5.4, 0.9)
**1.5 to <3.5 g/g**	52 (19)	4.3 (3.0, 7.0)	30 (58)	0.53 (0.38, 0.66)	0.34 (0.20, 0.48)	0.59 (0.36, 0.96)	0.57 (0.34, 0.93)	45	-6.8 (14.2)	-5.8 (-10.8, -1.0)
**≥3.5 g/g**	48 (17)	8.9 (5.7, 18.6)	34 (71)	0.35 (0.22, 0.48)	0.25 (0.12, 0.39)	Ref	Ref	43	-23.4 (33.7)	-13.6 (-29.1, -3.0)

Notes eGFR, estimated glomerular filtration rate; FSGS, Focal Segmental Glomerulosclerosis.

Supplemental Table 10. Clinical outcomes for incident FSGS-biopsy proteinuria analysis population: Proteinuria response category approach applying time-averaged proteinuria within 6-12 months post-baseline

	n (%)	Proteinuria at disease onset (g/g)	Kidney failure or death event	Kidney survival rate, proportion (95% CI)		Kidney failure risk (10-year), Hazard Ratio (95% Wald CL)		n	eGFR slope, 6 months to 10 years (mL/min/1.73m^2/year)	
**Time-averaged proteinuria, 6-12 months**		**Median (Q1, Q3)**	**n (%)**	**5-year**	**10-year**	**Unadjusted**	**Adjusted**		**Mean (SD)**	**Median (IQR)**
**Combined**	234 (100)	6.1 (3.7, 9.7)	79 (34)	0.69 (0.62, 0.75)	0.58 (0.50, 0.65)	NA	NA	222	-7.1 (21.1)	-3.0 (-8.7, 0.3)
**<0.3 g/g**	1 (0)	1.5 (1.5, 1.5)	0 (0)	1.00 (1.00, 1.00)	1.00 (1.00, 1.00)	0.00 (0.00, .)	0.00 (0.00, .)	1	-4.1 (.)	-4.1 (-4.1, -4.1)
**<0.5 g/g**	8 (3)	3.4 (1.8, 5.5)	0 (0)	1.00 (1.00, 1.00)	1.00 (1.00, 1.00)	0.00 (0.00, .)	0.00 (0.00, .)	7	-2.2 (1.1)	-2.2 (-2.7, -1.4)
**<0.75 g/g**	12 (5)	4.1 (1.8, 5.5)	0 (0)	1.00 (1.00, 1.00)	1.00 (1.00, 1.00)	0.00 (0.00, .)	0.00 (0.00, .)	11	-1.6 (1.6)	-1.9 (-2.7, -0.7)
**<1.0 g/g**	20 (9)	3.8 (1.8, 5.6)	1 (5)	0.95 (0.69, 0.99)	0.95 (0.69, 0.99)	0.10 (0.01, 0.72)	0.10 (0.01, 0.70)	18	-1.3 (2.7)	-1.7 (-2.7, -0.6)
**<1.5 g/g**	47 (20)	4.8 (2.3, 7.2)	4 (9)	0.92 (0.78, 0.98)	0.88 (0.70, 0.96)	0.16 (0.06, 0.43)	0.15 (0.05, 0.41)	45	-1.1 (3.6)	-1.2 (-3.8, 1.7)
**1.5 to <3.5 g/g**	92 (39)	4.0 (2.7, 6.8)	28 (30)	0.67 (0.56, 0.76)	0.63 (0.51, 0.73)	0.62 (0.39, 0.99)	0.58 (0.36, 0.95)	88	-2.8 (15.1)	-2.9 (-7.4, 0.5)
**≥3.5 g/g**	95 (41)	9.3 (6.1, 15.5)	47 (49)	0.59 (0.48, 0.69)	0.43 (0.31, 0.54)	Ref	Ref	89	-14.3 (28.3)	-6.6 (-18.6, -0.3)

Notes eGFR, estimated glomerular filtration rate; FSGS, Focal Segmental Glomerulosclerosis.

Supplemental Table 11. Clinical outcomes for incident (A) FSGS-biopsy and (B) MCD-biopsy proteinuria analysis populations: complete remission *(*CR*)*, FSGS partial remission *(*FPR*)* and Threshold approaches applying time-averaged proteinuria within 6-12 months post-baseline

(A)

Incident FSGS-biopsy	n	Kidney survival rate, proportion (95% CI)		Kidney failure risk (10-year), Hazard Ratio (95% Wald CL)		n	eGFR slope, 6-30 months (mL/min/1.73m^2/year)		n	eGFR slope, 6 months to 10 years (mL/min/1.73m^2/year)	
**Time-averaged proteinuria, 6-12 months**		**5-year**	**10-year**	**Unadjusted**	**Adjusted**		**Mean (SD)**	**Median (IQR)**		**Mean (SD)**	**Median (IQR)**
**Combined**	234	0.69 (0.62, 0.75)	0.58 (0.50, 0.65)	N/A	N/A	210	-8.1 (23.9)	-4.3 (-14.0, 2.0)	219	-7.7 (22.2)	-3.0 (-9.3, 0.4)
**Classical**											
**CR**	1	1.00 (1.00, 1.00)	NE	0 (0, NE)	0 (0,NE)	1	-4.6 (.)	-4.6 (-4.6, -4.6)	1	-4.1 (.)	-4.1 (-4.1, -4.1)
**PR**	76	0.94 (0.84, 0.98)	0.94 (0.84, 0.98)	0.10 (0.04, 0.26)	0.09 (0.03, 0.26)	74	-1.6 (18.7)	-0.2 (-8.3, 3.3)	75	-0.6 (16.5)	-1.1 (-4.1, 2.0)
**NR**	157	0.58 (0.49, 0.65)	0.44 (0.35, 0.53)	Ref	Ref	135	-11.6 (25.8)	-6.0 (-15.8, 0.5)	143	-11.5 (23.9)	-5.9 (-14.8, -0.4)
**FPR**											
**CR**	1	1.00 (1.00, 1.00)	NE	0 (0, NE)	0 (0, NE)	1	-4.6 (.)	-4.6 (-4.6, -4.6)	1	-4.1 (.)	-4.1 (-4.1, -4.1)
**FPR**	35	1.00 (1.00, 1.00)	1.00 (1.00, 1.00)	0 (0, NE)	0 (0, NE)	34	-1.9 (9.7)	0.0 (-4.4, 3.3)	34	-0.6 (3.1)	-1.2 (-2.7, 1.9)
**N-FPR**	198	0.64 (0.56, 0.70)	0.52 (0.43, 0.60)	Ref	Ref	175	-9.3 (25.7)	-5.5 (-14.1, 1.9)	184	-9.1 (23.9)	-4.4 (-11.9, 0.1)
**Threshold**											
**<0.3 g/g**	1	1.00 (1.00, 1.00)	NE	0 (0, NE)	0 (0, NE)	1	-4.6 (.)	-4.6 (-4.6, -4.6)	1	-4.1 (.)	-4.1 (-4.1, -4.1)
**0.3 to <1.5 g/g**	46	0.92 (0.77, 0.98)	0.88 (0.69, 0.95)	0.16 (0.06, 0.44)	0.10 (0.03, 0.29)	43	-1.6 (9.0)	-0.2 (-4.9, 3.3)	43	-0.9 (3.7)	-1.2 (-3.6, 1.9)
**1.5 to <3.5 g/g**	92	0.67 (0.56, 0.76)	0.63 (0.51, 0.73)	0.62 (0.39, 0.98)	0.35 (0.19, 0.63)	80	-3.6 (19.5)	-3.8 (-9.5, 2.1)	86	-3.4 (17.7)	-2.3 (-7.3, 0.9)
**≥3.5 g/g**	95	0.59 (0.48, 0.69)	0.43 (0.31, 0.54)	Ref	Ref	86	-15.5 (30.2)	-8.9 (-20.3, 0.1)	89	-15.2 (28.5)	-7.1 (-20.0, -1.0)

(B)

Incident MCD-biopsy<br>Time-averaged proteinuria, 6-12 months	n	Kidney survival rate, proportion (95% CI)		Kidney failure risk (10-year), Hazard Ratio (95% Wald CL)		n	eGFR slope, 6-30 months (mL/min/1.73m²/year)		n	eGFR slope, 6 months to 10 years (mL/min/1.73m²/year)	
		5-year	10-year	Unadjusted	Adjusted		Mean (SD)	Median (IQR)		Mean (SD)	Median (IQR)
**Combined**	228	0.90 (0.85, 0.93)	0.83 (0.74, 0.88)	N/A	N/A	204	-3.0 (11.1)	-0.8 (-8.7, 3.3)	215	-2.9 (7.2)	-1.0 (-4.6, 0.6)
**Classical**											
**CR**	10	1.00 (1.00, 1.00)	1.00 (1.00, 1.00)	0. (0, NE)	0. (0, NE)	10	-3.8 (9.3)	-0.9 (-9.1, 2.6)	10	-3.5 (7.1)	-1.5 (-5.0, 1.4)
**PR**	133	0.94 (0.88, 0.97)	0.87 (0.71, 0.94)	0.38 (0.17, 0.86)	0.33 (0.13, 0.80)	116	-2.0 (8.8)	-0.3 (-6.3, 3.5)	123	-2.1 (6.0)	-0.8 (-3.4, 0.6)
**NR**	85	0.83 (0.72, 0.90)	0.75 (0.63, 0.84)	Ref	Ref	78	-4.3 (14.0)	-2.5 (-13.3, 3.0)	82	-4.1 (8.6)	-1.3 (-7.0, 0.7)
**FPR**											
**CR**	10	1.00 (1.00, 1.00)	1.00 (1.00, 1.00)	0. (0, NE)	0. (0, NE)	10	-3.8 (9.3)	-0.9 (-9.1, 2.6)	10	-3.5 (7.1)	-1.5 (-5.0, 1.4)
**FPR**	71	0.96 (0.85, 0.99)	0.96 (0.85, 0.99)	0.19 (0.04, 0.80)	0.21 (0.05, 0.93)	63	-2.4 (8.6)	-0.5 (-6.2, 3.3)	66	-1.7 (4.6)	-1.0 (-2.9, 0.4)
**N-FPR**	147	0.87 (0.79, 0.92)	0.77 (0.66, 0.85)	Ref	Ref	131	-3.2 (12.3)	-1.1 (-10.8, 3.5)	139	-3.4 (8.2)	-1.0 (-5.7, 0.8)
**Threshold**											
**<0.3 g/g**	10	1.00 (1.00, 1.00)	1.00 (1.00, 1.00)	0. (0, NE)	0. (0, NE)	10	-3.8 (9.3)	-0.9 (-9.1, 2.6)	10	-3.5 (7.1)	-1.5 (-5.0, 1.4)
**0.3 to <1.5 g/g**	74	0.96 (0.85, 0.99)	0.96 (0.85, 0.99)	0.10 (0.02, 0.43)	0.08 (0.02, 0.37)	66	-2.4 (8.8)	-0.6 (-6.2, 3.3)	69	-1.9 (4.8)	-1.1 (-2.9, 0.4)
**1.5 to <3.5 g/g**	91	0.94 (0.85, 0.97)	0.84 (0.68, 0.93)	0.30 (0.13, 0.69)	0.20 (0.08, 0.52)	79	-2.0 (9.5)	-0.2 (-5.3, 3.6)	84	-2.3 (6.8)	-0.4 (-3.9, 0.8)
**≥3.5 g/g**	53	0.76 (0.61, 0.85)	0.64 (0.47, 0.77)	Ref	Ref	49	-5.1 (15.7)	-5.4 (-15.3, 1.1)	52	-5.2 (9.8)	-1.8 (-8.1, 0.8)

Notes: CR, complete remission; eGFR, estimated glomerular filtration rate; FPR, FSGS partial remission; FSGS, Focal Segmental Glomerulosclerosis; MCD, minimal change disease; NR, no remission; PR, partial remission; NE, not estimable (no events in group).

Supplemental Table 12. Clinical outcomes for prevalent (A) FSGS-biopsy and (B) MCD-biopsy proteinuria analysis populations: complete remission (CR), FSGS partial remission (FPR) and Threshold approaches applying time-averaged proteinuria within 6-12 months post-baseline

(A)

**Prevalent FSGS-biopsy**	**n**	**Kidney survival rate, proportion (95% CI)**		**Kidney failure risk (10-year), Hazard Ratio (95% Wald CL)**		**n**	**eGFR slope, 6-30 months (mL/min/1.73m^2/year)**		**n**	**eGFR slope, 6 months to 10 years (mL/min/1.73m^2/year)**	
**Time-averaged proteinuria, 6-12 months**		**5-year**	**10-year**	**Unadjusted**	**Adjusted**		**Mean (SD)**	**Median (IQR)**		**Mean (SD)**	**Median (IQR)**
**Combined**	364	0.70 (0.65, 0.75)	0.54 (0.47, 0.61)	N/A	N/A	308	-5.1 (16.6)	-3.7 (-10.8, 0.6)	324	-5.7 (11.0)	-3.3 (-8.4, -0.6)
**Classical**											
**CR**	13	1.00 (1.00, 1.00)	1.00 (1.00, 1.00)	0 (0, NE)	0 (0, NE)	13	0.0 (7.6)	-1.4 (-3.3, 0.2)	13	-0.4 (2.5)	-1.0 (-2.0, 1.1)
**PR**	95	0.92 (0.84, 0.97)	0.83 (0.70, 0.91)	0.20 (0.10, 0.38)	0.18 (0.09, 0.35)	78	-0.3 (18.6)	-1.3 (-5.1, 2.1)	84	-2.4 (5.9)	-1.1 (-4.5, 0.4)
**NR**	256	0.61 (0.55, 0.67)	0.43 (0.35, 0.51)	Ref	Ref	217	-7.2 (15.8)	-5.0 (-13.5, 0.0)	227	-7.3 (12.3)	-4.5 (-10.9, -1.2)
**FPR**											
**CR**	13	1.00 (1.00, 1.00)	1.00 (1.00, 1.00)	0 (0, NE)	0 (0, NE)	13	0.0 (7.6)	-1.4 (-3.3, 0.2)	13	-0.4 (2.5)	-1.0 (-2.0, 1.1)
**FPR**	64	0.92 (0.80, 0.97)	0.81 (0.61, 0.91)	0.23 (0.11, 0.50)	0.24 (0.11, 0.53)	52	1.3 (21.1)	-1.2 (-4.5, 2.8)	56	-1.5 (4.9)	-0.7 (-2.3, 0.5)
**N-FPR**	287	0.65 (0.58, 0.70)	0.48 (0.40, 0.55)	Ref	Ref	243	-6.8 (15.5)	-4.8 (-12.9, 0.1)	255	-7.0 (11.9)	-4.3 (-10.3, -1.1)
**Threshold**											
**<0.3 g/g**	13	1.00 (1.00, 1.00)	1.00 (1.00, 1.00)	0 (0, NE)	0 (0, NE)	13	0.0 (7.6)	-1.4 (-3.3, 0.2)	13	-0.4 (2.5)	-1.0 (-2.0, 1.1)
**0.3 to <1.5 g/g**	104	0.87 (0.78, 0.93)	0.77 (0.63, 0.86)	0.21 (0.12, 0.37)	0.19 (0.11, 0.35)	86	-0.2 (17.1)	-1.4 (-4.9, 2.0)	92	-2.1 (4.8)	-1.6 (-4.6, 0.2)
**1.5 to <3.5 g/g**	126	0.75 (0.65, 0.82)	0.55 (0.42, 0.66)	0.46 (0.31, 0.69)	0.40 (0.25, 0.63)	106	-4.0 (13.9)	-4.5 (-9.7, 0.1)	113	-4.5 (7.0)	-3.3 (-6.9, -1.0)
**≥3.5 g/g**	121	0.50 (0.40, 0.58)	0.35 (0.26, 0.45)	Ref	Ref	103	-11.0 (17.8)	-7.5 (-16.1, -1.0)	106	-10.9 (16.0)	-6.9 (-14.4, -2.2)

(B)

Prevalent MCD-biopsy<br><br>Time-averaged proteinuria, 6-12 months	n	Kidney survival rate, proportion (95% CI)<br>5-year	 10-year	Kidney failure risk (10-year), Hazard Ratio (95% Wald CL)<br>Unadjusted	 Adjusted	n	eGFR slope, 6-30 months (mL/min/1.73m2/year)<br>Mean (SD)	 Median (IQR)	n	eGFR slope, 6 months to 10 years (mL/min/1.73m2/year)<br>Mean (SD)	 Median (IQR)
**Combined**	365	0.89 (0.85, 0.92)	0.79 (0.72, 0.85)	N/A	N/A	333	-3.8 (14.4)	-1.1 (-7.6, 3.1)	353	-3.9 (11.9)	-1.2 (-4.6, 0.6)
**Classical**											
**CR**	52	1.00 (1.00, 1.00)	1.00 (1.00, 1.00)	0 (0, NE)	0 (0, NE)	45	-0.8 (6.9)	-1.3 (-4.3, 3.4)	49	-1.3 (3.1)	-1.0 (-1.8, 0.1)
**PR**	175	0.95 (0.89, 0.97)	0.83 (0.72, 0.90)	0.33 (0.18, 0.63)	0.35 (0.18, 0.69)	164	-2.6 (12.3)	-0.4 (-5.6, 3.1)	169	-2.3 (9.7)	-0.7 (-3.8, 0.8)
**NR**	138	0.78 (0.69, 0.85)	0.66 (0.54, 0.76)	Ref	Ref	124	-6.5 (18.2)	-3.6 (-14.0, 2.6)	135	-6.7 (15.4)	-2.8 (-8.6, 0.4)
**FPR**											
**CR**	52	1.00 (1.00, 1.00)	1.00 (1.00, 1.00)	0 (0, NE)	0 (0, NE)	45	-0.8 (6.9)	-1.3 (-4.3, 3.4)	49	-1.3 (3.1)	-1.0 (-1.8, 0.1)
**FPR**	115	0.94 (0.85, 0.97)	0.80 (0.64, 0.90)	0.47 (0.23, 0.96)	0.71 (0.34, 1.48)	112	-1.2 (10.1)	-0.4 (-4.9, 3.0)	114	-1.7 (8.7)	-0.7 (-3.6, 1.0)
**N-FPR**	198	0.84 (0.77, 0.89)	0.73 (0.63, 0.80)	Ref	Ref	176	-6.2 (17.4)	-2.4 (-13.6, 3.1)	190	-5.8 (14.5)	-1.8 (-7.2, 0.7)
**Threshold**											
**<0.3 g/g**	52	1.00 (1.00, 1.00)	1.00 (1.00, 1.00)	0 (0, NE)	0 (0, NE)	45	-0.8 (6.9)	-1.3 (-4.3, 3.4)	49	-1.3 (3.1)	-1.0 (-1.8, 0.1)
**0.3 to <1.5 g/g**	149	0.94 (0.87, 0.97)	0.83 (0.70, 0.91)	0.28 (0.13, 0.59)	0.32 (0.13, 0.75)	142	-1.7 (10.1)	-0.6 (-5.4, 3.0)	146	-1.7 (8.2)	-0.7 (-3.8, 1.1)
**1.5 to <3.5 g/g**	103	0.85 (0.75, 0.91)	0.71 (0.56, 0.82)	0.67 (0.34, 1.32)	0.55 (0.26, 1.16)	92	-5.0 (13.7)	-1.3 (-9.5, 2.6)	99	-4.8 (11.3)	-2.5 (-6.1, 0.5)
**≥3.5 g/g**	61	0.76 (0.62, 0.85)	0.65 (0.47, 0.78)	Ref	Ref	54	-9.7 (24.6)	-5.2 (-18.6, 3.7)	59	-9.8 (20.4)	-2.4 (-11.7, 0.4)

Notes: CR, complete remission; eGFR, estimated glomerular filtration rate; FPR, FSGS partial remission; FSGS, Focal Segmental Glomerulosclerosis; MCD, minimal change disease; NR, no remission; PR, partial remission; NE, not estimable (no events in group).

Supplemental Table 13. Clinical outcomes for prevalent (A) FSGS-biopsy and (B) MCD-biopsy proteinuria analysis populations: complete remission *(*CR*)*, FSGS partial remission *(*FPR*)*and Threshold approaches applying time-averaged proteinuria within 6-24 months post-baseline

(A)

Prevalent FSGS-biopsy<br><br>Time-averaged proteinuria, 6-24 months	n	Kidney survival rate, proportion (95% CI)		Kidney failure risk (10-year), Hazard Ratio (95% Wald CL)		n	eGFR slope, 6-30 months (mL/min/1.73m^2/year)		n	eGFR slope, 6 months to 10 years (mL/min/1.73m^2/year)	
		5-year	10-year	Unadjusted	Adjusted		Mean (SD)	Median (IQR)		Mean (SD)	Median (IQR)
Combined	428	0.72 (0.67, 0.76)	0.55 (0.49, 0.61)	N/A	N/A	356	-4.9 (16.2)	-3.5 (-10.1, 0.9)	379	-5.6 (11.1)	-3.3 (-7.9, -0.6)
**Classical**											
CR	24	1.00 (1.00, 1.00)	1.00 (1.00, 1.00)	0 (0, NE)	0 (0, NE)	22	-0.4 (6.2)	-1.4 (-3.4, 1.6)	23	-0.3 (3.5)	-0.5 (-2.1, 1.0)
PR	116	0.93 (0.86, 0.97)	0.76 (0.61, 0.86)	0.23 (0.13, 0.41)	0.16 (0.09, 0.30)	93	-1.0 (17.5)	-1.4 (-5.4, 2.8)	101	-1.6 (5.2)	-1.1 (-4.3, 0.5)
NR	288	0.62 (0.56, 0.67)	0.45 (0.38, 0.52)	Ref	Ref	241	-6.8 (16.1)	-5.2 (-12.6, 0.1)	255	-7.7 (12.6)	-4.8 (-11.2, -1.4)
**FPR**											
CR	24	1.00 (1.00, 1.00)	1.00 (1.00, 1.00)	0 (0, NE)	0 (0, NE)	22	-0.4 (6.2)	-1.4 (-3.4, 1.6)	23	-0.3 (3.5)	-0.5 (-2.1, 1.0)
FPR	82	0.98 (0.90, 1.00)	0.82 (0.63, 0.92)	0.16 (0.07, 0.35)	0.14 (0.06, 0.32)	64	0.0 (19.6)	-2.5 (-5.6, 2.0)	70	-1.3 (4.6)	-1.1 (-3.3, 0.3)
N-FPR	322	0.64 (0.58, 0.69)	0.47 (0.40, 0.54)	Ref	Ref	270	-6.4 (15.7)	-4.6 (-12.0, 0.3)	286	-7.1 (12.2)	-4.5 (-10.3, -1.3)
**Threshold**											
<0.3 g/g	24	1.00 (1.00, 1.00)	1.00 (1.00, 1.00)	0 (0, NE)	0 (0, NE)	22	-0.4 (6.2)	-1.4 (-3.4, 1.6)	23	-0.3 (3.5)	-0.5 (-2.1, 1.0)
0.3 to <1.5 g/g	138	0.90 (0.82, 0.94)	0.76 (0.63, 0.84)	0.21 (0.13, 0.35)	0.16 (0.09, 0.28)	115	-0.7 (16.2)	-1.6 (-5.6, 2.1)	122	-1.8 (5.2)	-1.4 (-4.3, 0.0)
1.5 to <3.5 g/g	130	0.70 (0.61, 0.78)	0.51 (0.39, 0.61)	0.59 (0.41, 0.84)	0.42 (0.28, 0.63)	105	-4.6 (11.7)	-4.5 (-9.3, 0.1)	115	-4.6 (6.9)	-3.7 (-7.0, -1.4)
≥3.5 g/g	136	0.53 (0.44, 0.61)	0.38 (0.28, 0.47)	Ref	Ref	114	-10.3 (19.5)	-8.7 (-18.9, -0.5)	119	-11.5 (16.2)	-7.9 (-16.7, -1.6)

(B)

Prevalent MCD-biopsy<br><br>Time-averaged proteinuria, 6-24 months	n	Kidney survival rate, proportion (95% CI)		Kidney failure risk (10-year), Hazard Ratio (95% Wald CL)		n	eGFR slope, 6-30 months (mL/min/1.73m²/year)		n	eGFR slope, 6 months to 10 years (mL/min/1.73m²/year)	
		**5-year**	**10-year**	**Unadjusted**	**Adjusted**		**Mean (SD)**	**Median (IQR)**		**Mean (SD)**	**Median (IQR)**
**Combined**	452	0.91 (0.87, 0.93)	0.80 (0.74, 0.85)	N/A	N/A	407	-3.3 (14.0)	-1.2 (-7.2, 3.4)	436	-3.5 (11.1)	-1.3 (-4.3, 0.6)
**Classical**											
**CR**	84	0.97 (0.87, 0.99)	0.90 (0.73, 0.96)	0.18 (0.06, 0.51)	0.25 (0.09, 0.73)	77	-1.7 (10.6)	-0.9 (-4.4, 2.8)	81	-1.6 (8.4)	-0.8 (-2.1, 0.4)
**PR**	229	0.95 (0.90, 0.97)	0.89 (0.82, 0.94)	0.24 (0.13, 0.44)	0.22 (0.11, 0.43)	211	-2.4 (11.1)	-1.0 (-6.6, 3.4)	222	-1.9 (7.9)	-1.0 (-3.7, 0.7)
**NR**	139	0.80 (0.72, 0.87)	0.64 (0.52, 0.73)	Ref	Ref	119	-5.9 (19.4)	-3.7 (-11.6, 3.7)	133	-7.5 (15.4)	-2.8 (-11.4, 0.4)
**FPR**											
**CR**	84	0.97 (0.87, 0.99)	0.90 (0.73, 0.96)	0.23 (0.08, 0.65)	0.33 (0.12, 0.96)	77	-1.7 (10.6)	-0.9 (-4.4, 2.8)	81	-1.6 (8.4)	-0.8 (-2.1, 0.4)
**FPR**	170	0.97 (0.91, 0.99)	0.89 (0.79, 0.94)	0.27 (0.13, 0.55)	0.26 (0.12, 0.57)	159	-1.6 (8.4)	-0.8 (-5.9, 3.2)	166	-1.3 (6.0)	-0.9 (-3.5, 0.7)
**N-FPR**	198	0.83 (0.77, 0.88)	0.71 (0.61, 0.78)	Ref	Ref	171	-5.7 (18.6)	-3.2 (-11.6, 3.8)	189	-6.4 (14.5)	-2.4 (-7.3, 0.4)
**Threshold**											
**<0.3 g/g**	84	0.97 (0.87, 0.99)	0.90 (0.73, 0.96)	0.15 (0.05, 0.43)	0.16 (0.05, 0.50)	77	-1.7 (10.6)	-0.9 (-4.4, 2.8)	81	-1.6 (8.4)	-0.8 (-2.1, 0.4)
**0.3 to <1.5 g/g**	199	0.97 (0.92, 0.99)	0.89 (0.80, 0.94)	0.16 (0.08, 0.35)	0.11 (0.04, 0.27)	186	-1.6 (8.8)	-0.9 (-6.0, 3.1)	194	-1.4 (6.1)	-0.9 (-3.6, 0.8)
**1.5 to <3.5 g/g**	105	0.83 (0.73, 0.89)	0.75 (0.62, 0.84)	0.56 (0.30, 1.06)	0.45 (0.23, 0.89)	91	-5.6 (17.1)	-3.3 (-13.7, 4.3)	100	-5.3 (12.7)	-2.5 (-5.7, 0.5)
**≥3.5 g/g**	64	0.78 (0.65, 0.87)	0.59 (0.42, 0.72)	Ref	Ref	53	-8.0 (23.3)	-3.7 (-15.0, 3.5)	61	-10.3 (18.6)	-4.8 (-18.2, -0.2)

Notes: CR, complete remission; eGFR, estimated glomerular filtration rate; FPR, FSGS partial remission; FSGS, Focal Segmental Glomerulosclerosis; MCD, minimal change disease; NR, no remission; PR, partial remission; NE, not estimable (no events in group).

Supplemental Table 14. Clinical outcomes for prevalent FSGS-biopsy proteinuria analysis population: Proteinuria response category approach applying lowest proteinuria value within (A) 6-12 months and (B) 6-24 months post-baseline

(A)

	n (%)	Proteinuria at disease onset (g/g)	Kidney failure or death event	Kidney survival rate, proportion (95% CI)		Kidney failure risk (10-year), Hazard Ratio (95% Wald CL)		n	eGFR slope, 6 months to 10 years (mL/min/1.73m^2/year)	
**Proteinuria response category:**		**Median (Q1, Q3)**	**n (%)**	**5-year**	**10-year**	**Unadjusted**	**Adjusted**		**Mean (SD)**	**Median (IQR)**
**6-12 months**										
**Combined**	364 (100)	3.4 (2.1, 5.8)	124 (34)	0.70 (0.65, 0.75)	0.54 (0.47, 0.61)	NA	NA	332	-5.4 (10.2)	-3.3 (-7.8, -0.5)
**<0.3 g/g**	92 (25)	3.4 (2.2, 5.9)	8 (9)	0.95 (0.86, 0.98)	0.82 (0.65, 0.91)	0.10 (0.05, 0.22)	0.10 (0.05, 0.22)	84	-1.9 (5.0)	-1.1 (-2.9, 0.4)
**<0.5 g/g**	111 (30)	3.4 (2.1, 5.5)	13 (12)	0.93 (0.85, 0.96)	0.78 (0.63, 0.88)	0.15 (0.08, 0.27)	0.14 (0.08, 0.27)	99	-2.0 (6.1	-1.3 (-3.4, 0.2)
**<0.75 g/g**	134 (37)	3.4 (2.2, 5.5)	17 (13)	0.91 (0.84, 0.95)	0.78 (0.65, 0.87)	0.16 (0.09, 0.28)	0.16 (0.09, 0.28)	121	-2.5 (7.0)	-1.3 (-4.5, 0.4)
**<1.0 g/g**	155 (43)	3.3 (2.0, 5.1)	25 (16)	0.86 (0.79, 0.91)	0.75 (0.64, 0.83)	0.21 (0.13, 0.34)	0.20 (0.12, 0.33)	141	-2.8 (7.1)	-1.6 (-4.9, 0.2)
**<1.5 g/g**	193 (53)	3.0 (2.0, 4.5)	35 (18)	0.86 (0.80, 0.91)	0.70 (0.59, 0.78)	0.23 (0.15, 0.36)	0.22 (0.14, 0.35)	177	-3.2 (7.2)	-1.8 (-5.4, -0.1)
**1.5 to <3.5 g/g**	89 (24)	3.0 (2.1, 4.5)	39 (44)	0.57 (0.45, 0.67)	0.45 (0.31, 0.57)	0.69 (0.45, 1.05)	0.64 (0.42, 0.98)	81	-4.6 (7.1)	-3.8 (-8.0, -1.0)
**≥3.5 g/g**	82 (23)	5.7 (4.2, 8.4)	50 (61)	0.50 (0.38, 0.60)	0.31 (0.20, 0.43)	Ref	Ref	74	-11.8 (15.4)	-7.7 (-14.8, -4.1)

(B)

	n (%)	Proteinuria at disease onset (g/g)	Kidney failure or death event	Kidney survival rate, proportion (95% CI)		Kidney failure risk (10-year), Hazard Ratio (95% Wald CL)		n	eGFR slope, 6 months to 10 years (mL/min/1.73m^2/year)	
**Proteinuria response category: 6-24 months**		**Median (Q1, Q3)**	**n (%)**	**5-year**	**10-year**	**Unadjusted**	**Adjusted**		**Mean (SD)**	**Median (IQR)**
**Combined**	428 (100)	3.3 (2.1, 5.7)	141 (33)	0.72 (0.67, 0.76)	0.55 (0.49, 0.61)	NA	NA	388	-5.3 (10.4)	-3.3 (-7.7, -0.5)
**<0.3 g/g**	136 (32)	3.4 (2.2, 6.0)	14 (10)	0.94 (0.87, 0.97)	0.82 (0.70, 0.89)	0.13 (0.07, 0.23)	0.12 (0.06, 0.22)	124	-1.7 (5.8)	-1.1 (-3.3, 0.6)
**<0.5 g/g**	170 (40)	3.3 (2.1, 5.3)	22 (13)	0.92 (0.86, 0.95)	0.78 (0.67, 0.86)	0.16 (0.10, 0.28)	0.16 (0.09, 0.27)	152	-1.9 (6.9)	-1.2 (-3.7, 0.6)
**<0.75 g/g**	201 (47)	3.3 (2.0, 5.1)	33 (16)	0.89 (0.83, 0.93)	0.72 (0.62, 0.80)	0.21 (0.13, 0.34)	0.20 (0.13, 0.33)	182	-2.3 (7.6)	-1.3 (-4.8, 0.6)
**<1.0 g/g**	224 (52)	3.1 (2.0, 4.7)	42 (19)	0.86 (0.80, 0.90)	0.71 (0.62, 0.78)	0.24 (0.16 ,0.38)	0.23 (0.15, 0.36)	205	-2.6 (7.6)	-1.7 (-5.2, 0.3)
**<1.5 g/g**	262 (61)	2.9 (1.9, 4.6)	52 (20)	0.84 (0.78, 0.88)	0.69 (0.60, 0.76)	0.26 (0.17, 0.40)	0.25 (0.16, 0.38)	241	-3.0 (7.7)	-2.0 (-5.4, 0.0)
**1.5 to <3.5 g/g**	95 (22)	3.3 (2.4, 4.8)	49 (52)	0.57 (0.46, 0.67)	0.36 (0.25, 0.48)	0.80 (0.53, 1.22)	0.74 (0.49, 1.14)	84	-6.0 (7.2)	-4.8 (-8.9, -2.1)
**≥3.5 g/g**	71 (17)	5.8 (4.0, 9.5)	40 (56)	0.51 (0.39, 0.62)	0.36 (0.23, 0.50)	Ref	Ref	63	-13.4 (17.0)	-8.7 (-18.3, -4.4)

Notes eGFR, estimated glomerular filtration rate; FSGS, Focal Segmental Glomerulosclerosis.

Supplemental Table 15. Clinical outcomes for prevalent FSGS-biopsy proteinuria analysis population: Proteinuria response category approach applying time-averaged proteinuria within (A) 6-12 months and (B) 6-24 months post-baseline

(A)

	n (%)	Proteinuria at disease onset (g/g)	Kidney failure or death event	Kidney survival rate, proportion (95% CI)		Kidney failure risk (10-year), Hazard Ratio (95% Wald CL)		n	eGFR slope, 6 months to 10 years (mL/min/1.73m^2/year)	
**Time-averaged proteinuria, 6-12 months**		**Median (Q1, Q3)**	**n (%)**	**5-year**	**10-year**	**Unadjusted**	**Adjusted**		**Mean (SD)**	**Median (IQR)**
**Combined**	364 (100)	3.4 (2.1, 5.8)	124 (34)	0.70 (0.65, 0.75)	0.54 (0.47, 0.61)	NA	NA	332	-5.4 (10.2)	-3.3 (-7.8, -0.5)
**<0.3 g/g**	13 (4)	3.3 (2.2, 6.1)	0 (0)	1.00 (1.00, 1.00)	1.00 (1.00, 1.00)	0.00 (0.00, NE)	0.00 (0.00, NE)	13	-0.4 (2.5)	-1.0 (-2.0, 1.1)
**<0.5 g/g**	23 (6)	3.1 (2.2, 6.1)	0 (0)	1.00 (1.00, 1.00)	1.00 (1.00, 1.00)	0.00 (0.00, NE)	0.00 (0.00, NE)	23	-1.1 (2.6)	-1.0 (-2.1, 0.4)
**<0.75 g/g**	48 (13)	2.7 (1.8, 3.9)	4 (8)	0.92 (0.78, 0.97)	0.88 (0.70, 0.95)	0.11 (0.04, 0.31)	0.13 (0.05, 0.35)	45	-2.2 (4.2)	-1.0 (-2.7, 0.1)
**<1.0 g/g**	66 (18)	2.6 (1.8, 4.2)	6 (9)	0.90 (0.79, 0.96)	0.87 (0.73, 0.94)	0.13 (0.06, 0.30)	0.13 (0.05, 0.30)	59	-2.0 (4.2)	-1.1 (-4.9, 0.3)
**<1.5 g/g**	117 (32)	2.6 (1.9, 4.0)	16 (14)	0.89 (0.81, 0.94)	0.79 (0.66, 0.87)	0.19 (0.11, 0.33)	0.17 (0.10, 0.29)	105	-1.9 (4.6)	-1.6 (-4.3, 0.2)
**1.5 to <3.5 g/g**	126 (35)	2.7 (2.0, 3.7)	39 (31)	0.75 (0.65, 0.82)	0.55 (0.42, 0.66)	0.47 (0.31, 0.69)	0.42 (0.28, 0.63)	117	-4.5 (6.8)	-3.3 (-7.2, -1.0)
**≥3.5 g/g**	121 (33)	5.7 (4.2, 8.0)	69 (57)	0.50 (0.40, 0.58)	0.35 (0.26, 0.45)	Ref	Ref	110	-9.8 (14.6)	-5.8 (-13.7, -2.3)

(B)

	n (%)	Proteinuria at disease onset (g/g)	Kidney failure or death event	Kidney survival rate, proportion (95% CI)		Kidney failure risk (10-year), Hazard Ratio (95% Wald CL)		n	eGFR slope, 6 months to 10 years (mL/min/1.73m^2/year)	
**Time-averaged proteinuria, 6-24 months**		**Median (Q1, Q3)**	**n (%)**	**5-year**	**10-year**	**Unadjusted**	**Adjusted**		**Mean (SD)**	**Median (IQR)**
**Combined**	428 (100)	3.3 (2.1, 5.7)	141 (33)	0.72 (0.67, 0.76)	0.55 (0.49, 0.61)	NA	NA	388	-5.3 (10.4)	-3.3 (-7.7, -0.5)
**<0.3 g/g**	24 (6)	3.3 (2.2, 6.3)	0 (0)	1.00 (1.00, 1.00)	1.00 (1.00, 1.00)	0.00 (0.00, NE)	0.00 (0.00, NE)	23	-0.3 (3.5)	-0.5 (-2.1, 1.0)
**<0.5 g/g**	45 (11)	2.9 (2.1, 4.6)	0 (0)	1.00 (1.00, 1.00)	1.00 (1.00, 1.00)	0.00 (0.00, NE)	0.00 (0.00, NE)	41	-1.1 (3.8)	-0.7 (-2.4, 0.5)
**<0.75 g/g**	75 (18)	3.0 (2.0, 4.7)	3 (4)	0.98 (0.90, 1.00)	0.91 (0.73, 0.97)	0.06 (0.02, 0.19)	0.05 (0.02, 0.17)	63	-0.7 (4.0)	-0.7 (-2.2, 0.8)
**<1.0 g/g**	103 (24)	2.9 (2.0, 5.0)	8 (8)	0.95 (0.87, 0.98)	0.84 (0.68, 0.92)	0.12 (0.06, 0.25)	0.11 (0.05, 0.22)	88	-1.3 (4.6)	-1.1 (-3.3, 0.5)
**<1.5 g/g**	162 (38)	2.6 (1.9, 4.2)	19 (12)	0.91 (0.85, 0.95)	0.79 (0.67, 0.87)	0.18 (0.11, 0.30)	0.15 (0.09, 0.25)	145	-1.6 (4.9)	-1.3 (-3.8, 0.1)
**1.5 to <3.5 g/g**	130 (30)	2.7 (2.0, 4.0)	48 (37)	0.70 (0.61, 0.78)	0.51 (0.39, 0.61)	0.59 (0.41, 0.84)	0.50 (0.34, 0.72)	120	-4.5 (6.7)	-3.7 (-6.9, -1.3)
**≥3.5 g/g**	136 (32)	5.6 (3.7, 8.4)	74 (54)	0.53 (0.44, 0.61)	0.38 (0.28, 0.47)	Ref	Ref	123	-10.6 (15.0)	-7.6 (-16.2, -2.2)

Notes eGFR, estimated glomerular filtration rate; FSGS, Focal Segmental Glomerulosclerosis; NE, not estimable (no events in group).

Supplemental Table 16. Univariable and multivariable analysis of 10-year survival for FSGS-biopsy and MCD-biopsy proteinuria analysis populations

			FSGS-biopsy				MCD-biopsy			
			Univariable analysis		Multivariable analysis		Univariable analysis		Multivariable analysis	
	Variable	Reference	Hazard Ratio (95% CI)	P > ChiSq	Hazard Ratio (95% CI)	P > ChiSq	Hazard Ratio (95% CI)	P > ChiSq	Hazard Ratio (95% CI)	P > ChiSq
**10-year survival**										
Age group (years) at disease onset	20, <40	<20	1.03 (0.81, 1.31)	0.79	0.93 (0.73, 1.19)	0.57	0.18 (0.07, 0.47)	0.00	0.16 (0.06, 0.41)	0.00
Age group (years) at disease onset	40, <60	<20	1.12 (0.89, 1.43)	0.34	0.92 (0.72, 1.18)	0.52	0.94 (0.57, 1.54)	0.79	0.69 (0.41, 1.18)	0.18
Age group (years) at disease onset	60+	<20	1.62 (1.25, 2.11)	0.00	0.92 (0.67, 1.25)	0.58	3.04 (1.99, 4.64)	<.0001	1.70 (1.03, 2.83)	0.04
Sex	Female	Male	0.81 (0.67, 0.97)	0.02	0.81 (0.67, 0.97)	0.02	0.68 (0.47, 0.99)	0.04	0.62 (0.42, 0.91)	0.02
Ethnicity	Asian	White	0.98 (0.73, 1.31)	0.88	0.99 (0.74, 1.34)	0.97	0.39 (0.19, 0.81)	0.01		
Ethnicity	Missing	White	0.77 (0.53, 1.11)	0.16	0.75 (0.52, 1.08)	0.13	0.50 (0.22, 1.14)	0.10		
Ethnicity	Other	White	1.45 (1.1, 1.9)	0.01	1.43 (1.08, 1.89)	0.01	1.02 (0.52, 2.03)	0.94		
Year of disease onset	2005, <2010	2010, <2015	0.85 (0.66, 1.11)	0.23	0.9 (0.69, 1.18)	0.46	0.73 (0.39, 1.34)	0.31	0.91 (0.48, 1.7)	0.76
Year of disease onset	2015+	2010, <2015	1.33 (1.05, 1.68)	0.02	1.15 (0.9, 1.46)	0.27	1.4 (0.92, 2.14)	0.12	1.21 (0.77, 1.88)	0.40
Year of disease onset	<2005	2010, <2015	0.72 (0.56, 0.93)	0.01	0.76 (0.58, 0.99)	0.04	0.4 (0.21, 0.78)	0.01	0.52 (0.25, 1.05)	0.07
eGFR category at disease onset	G3a	G1&2	2.06 (1.33, 3.17)	0.00	1.93 (1.23, 3.01)	0.00	0.92 (0.32, 2.64)	0.88	0.58 (0.2, 1.7)	0.32
eGFR category at disease onset	G3b	G1&2	3.48 (2.33, 5.18)	<.0001	3.46 (2.29, 5.24)	<.0001	4.24 (1.93, 9.35)	0.00	2.78 (1.22, 6.31)	0.01
eGFR category at disease onset	G4+5	G1&2	9.79 (6.61, 14.51)	<.0001	10.02 (6.51, 15.43)	<.0001	7.25 (3.74, 14.07)	<.0001	4.55 (2.25, 9.21)	<.0001
eGFR category at disease onset	Missing	G1&2	1.49 (1.12, 1.98)	0.01	1.91 (1.39, 2.63)	<.0001	0.79 (0.5, 1.23)	0.29	1.02 (0.61, 1.69)	0.94
PCR category (g/g) at disease onset	0, <1.5	3.5+	1.03 (0.61, 1.72)	0.92	1.23 (0.73, 2.08)	0.44	0.56 (0.2, 1.57)	0.27	0.70 (0.25, 2)	0.51
PCR category (g/g) at disease onset	1.5, <3.5	3.5+	1.30 (0.87, 1.93)	0.20	0.98 (0.65, 1.48)	0.91	0.41 (0.15, 1.14)	0.09	0.35 (0.12, 0.98)	0.05
PCR category (g/g) at disease onset	Missing	3.5+	0.72 (0.57, 0.9)	0.00	0.74 (0.56, 0.97)	0.03	0.37 (0.25, 0.55)	<.0001	0.58 (0.36, 0.91)	0.02

BMI category at disease onset	Quartile 1	Quartile 2	0.85 (0.51, 1.42)	0.53		2.45 (0.87, 6.87)	0.09	
BMI category at disease onset	Quartile 3	Quartile 2	1.30 (0.8, 2.14)	0.29		1.92 (0.63, 5.89)	0.25	
BMI category at disease onset	Missing	Quartile 2	0.73 (0.5, 1.05)	0.09		0.74 (0.3, 1.82)	0.51	
Albmin category at disease onset	Quartile 1	Quartile 3	0.96 (0.7, 1.31)	0.78		0.98 (0.56, 1.74)	0.96	
Albmin category at disease onset	Quartile 2	Quartile 3	0.94 (0.67, 1.33)	0.73		0.90 (0.49, 1.63)	0.72	
Albmin category at disease onset	Missing	Quartile 3	0.69 (0.53, 0.89)	0.00		0.45 (0.27, 0.73)	0.00	
SBP category at disease onset	Quartile 2	Quartile 1	1.08 (0.68, 1.71)	0.75		0.90 (0.37, 2.21)	0.82	
SBP category at disease onset	Quartile 3	Quartile 1	1.37 (0.87, 2.18)	0.18		1.24 (0.53, 2.92)	0.62	
SBP category at disease onset	Missing	Quartile 1	0.80 (0.56, 1.12)	0.19		0.46 (0.24, 0.88)	0.02	

Notes: BMI, body mass index; eGFR, estimated glomerular filtration rate; FSGS, Focal Segmental Glomerulosclerosis; MCD, minimal change disease.

Supplemental Table 17. Summary statistics for (A) the number of UPCR measurements per patient during follow-up windows and (B) the number of eGFR measurements per patient used to calculate eGFR slopes.

(A)

Analysis population	Group	Patients	Variable	N with data	Median (IQR)
Incident	FSGS	277	PCR count in 6-12month window	234	3 (1, 6)
			PCR count in 6-24month window	277	5 (2, 10)
Incident	MCD	260	PCR count in 6-12month window	228	3.5 (2, 6)
			PCR count in 6-24month window	260	7 (3, 13)
Prevalent	FSGS	428	PCR count in 6-12month window	364	2 (1, 4)
			PCR count in 6-24month window	428	5 (3, 9)
Prevalent	MCD	452	PCR count in 6-12month window	365	3 (1, 5)
			PCR count in 6-24month window	452	5 (2, 10)

(B)

Analysis population	Group	Patients	Variable	N with data	Median (IQR)
Incident	FSGS	277	eGFR count in 6-30month window	266	10 (5, 19)
			eGFR count in 6mos-10year window	271	23 (11, 41)
Incident	MCD	260	eGFR count in 6-30month window	251	12 (6, 21)
			eGFR count in 6mos-10year window	258	25 (14, 47)
Prevalent	FSGS	428	eGFR count in 6-30month window	409	10 (5, 16)
			eGFR count in 6mos-10year window	419	22 (12, 41)
evalent	MCD	452	eGFR count in 6-30month window	439	10 (5, 17)
			eGFR count in 6mos-10year window	451	22 (11, 42)

Notes: eGFR, estimated glomerular filtration rate; FSGS, Focal Segmental Glomerulosclerosis; INS, idiopathic nephrotic syndrome patients; MCD, minimal change disease; UPCR, urine protein:creatinine ratio.

Supplemental Figures

Supplemental Figure 1. (A) Summary of the total RaDaR INS population, and (B) eligibility criteria, patient disposition, and study attrition for the incident and prevalent proteinuria analysis populations of FSGS and MCD.

(A)

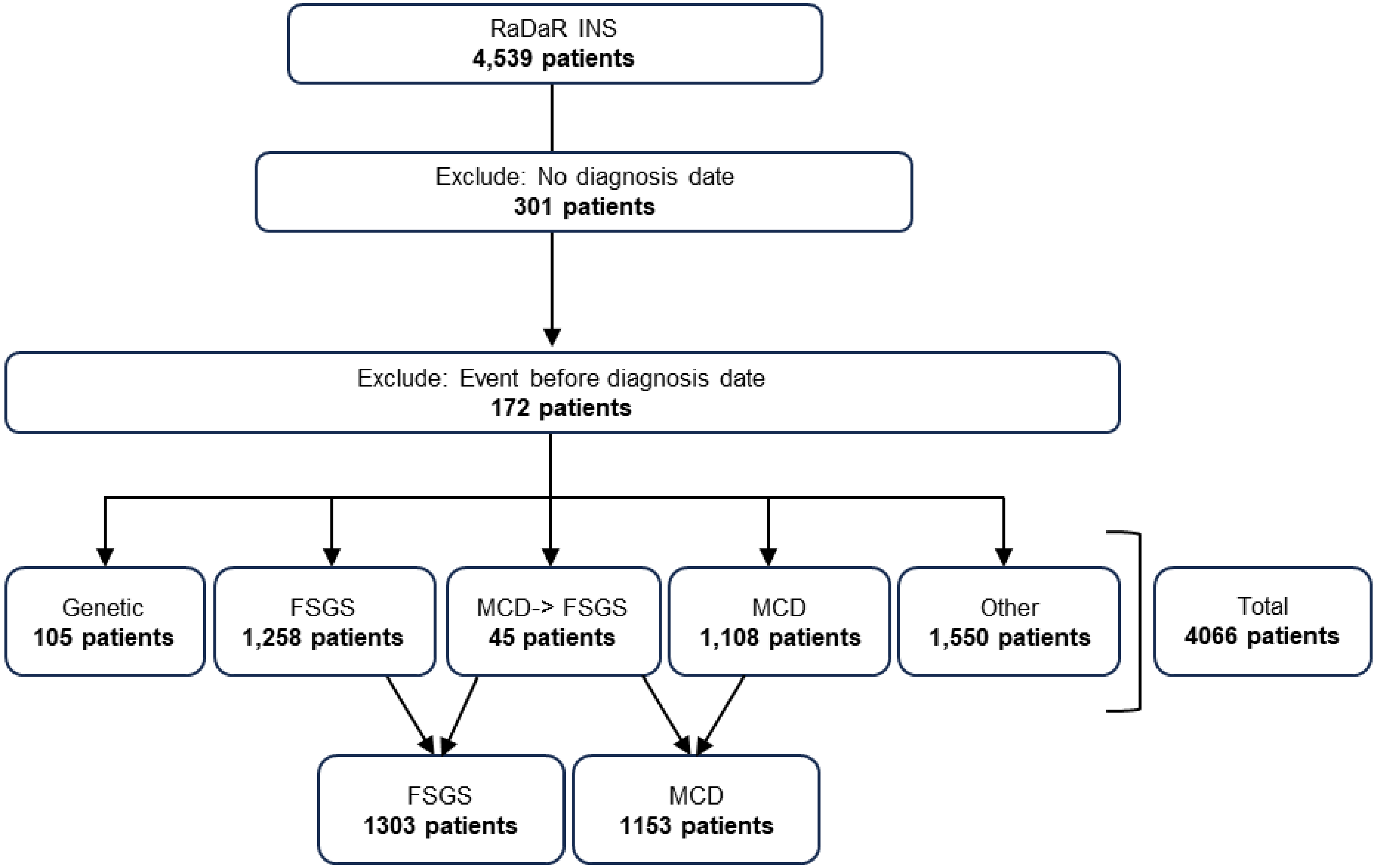

(B)

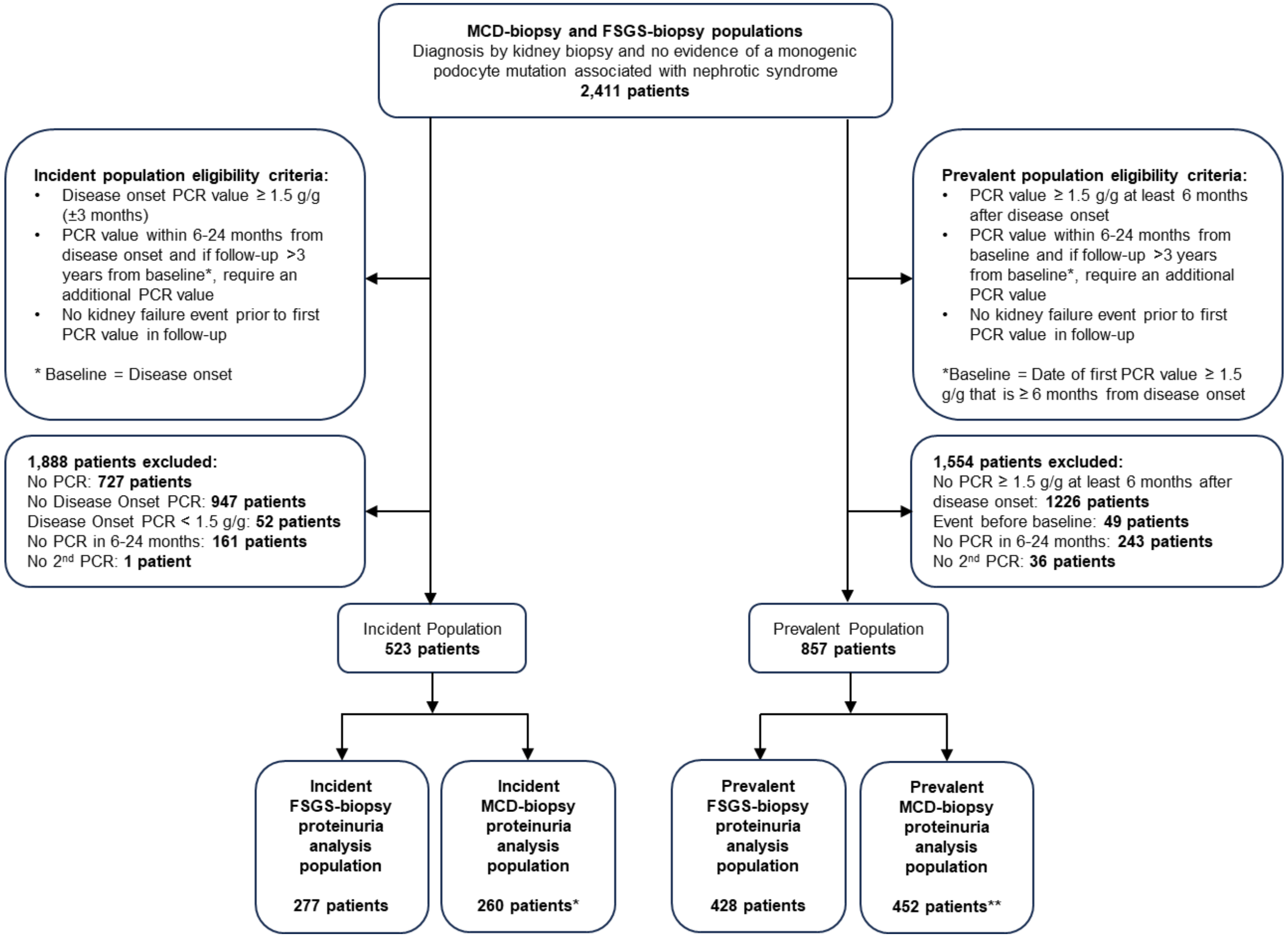

Notes: *14 MCD-biopsy patients progressing to FSGS-biopsy are included in both the MCD-biopsy and FSGS-biopsy incident proteinuria analysis populations; **23 MCD-biopsy patients progressing to FSGS-biopsy are included in both the MCD-biopsy and FSGS-biopsy prevalent proteinuria analysis populations.

Supplemental Figure 2. Venn diagram displaying the degree of overlap between proteinuria analysis populations within the idiopathic nephrotic syndrome (INS) cohort (A) FSGS-biopsy sub-group and (B) MCD-biopsy sub-group

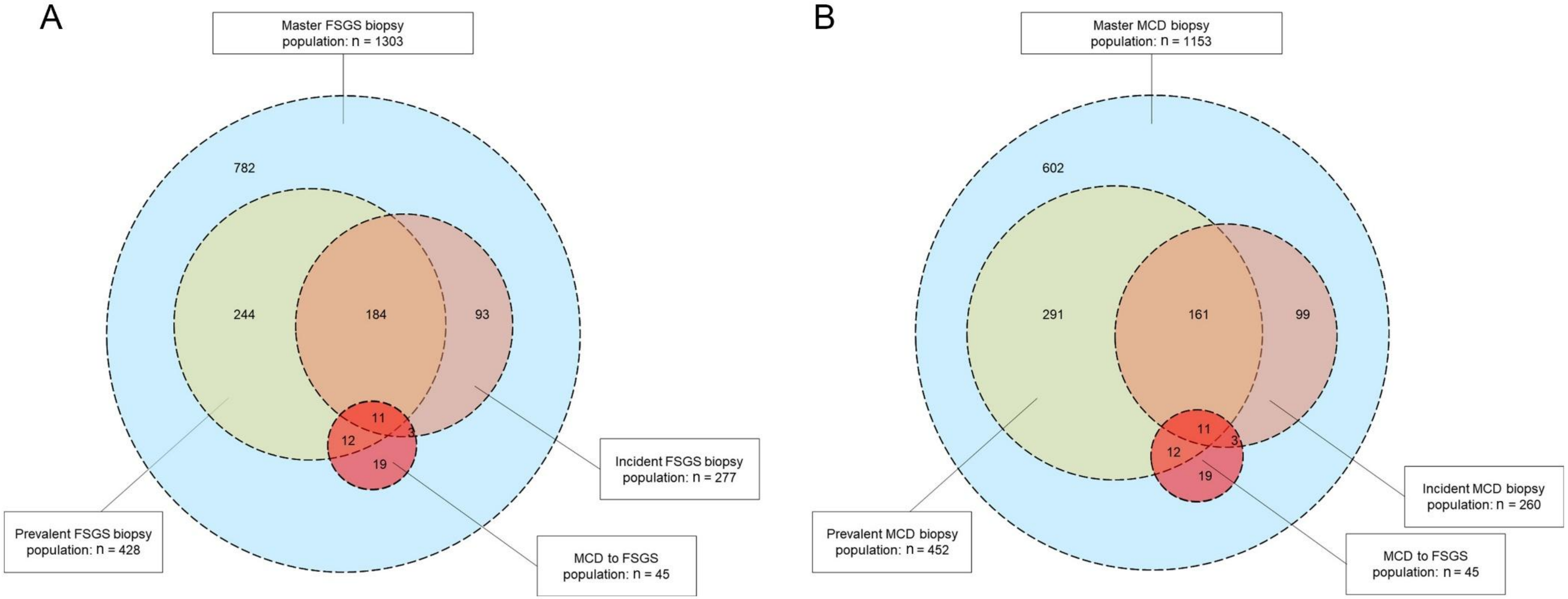

Notes: FSGS, Focal Segmental Glomerulosclerosis; MCD, minimal change disease.

Supplemental Figure 3. Kaplan-Meier survival curves by age at kidney failure/death for idiopathic nephrotic syndrome patients (A) idiopathic nephrotic syndrome (INS) cohort, (B) INS cohort – Pediatrics, (C) INS cohort – Adults, by diagnosis category

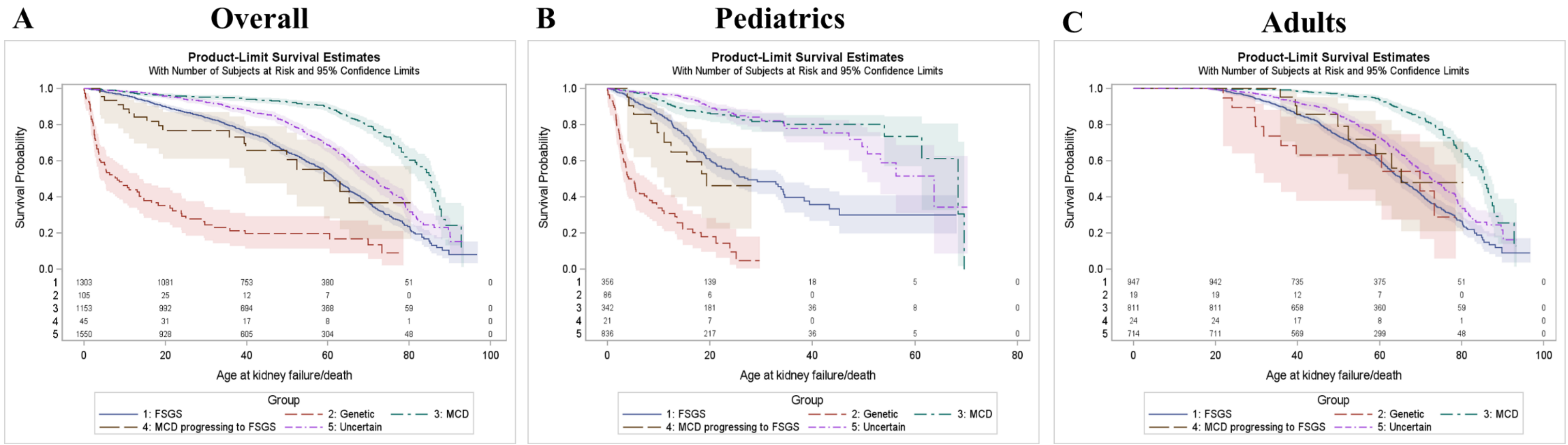

Notes: FSGS, Focal Segmental Glomerulosclerosis; INS, idiopathic nephrotic syndrome patients; MCD, minimal change disease.

Supplemental Figure 4. Kaplan-Meier survival curves for incident biopsy populations: (A) FSGS classical remissions, (B) FSGS partial remission, (C) FSGS threshold approach, (D) MCD classical remissions, (E) MCD partial remission, and (F) MCD threshold approaches. All analyses were based on the lowest proteinuria value recorded within 6-24 months post-baseline.

FSGS

MCD

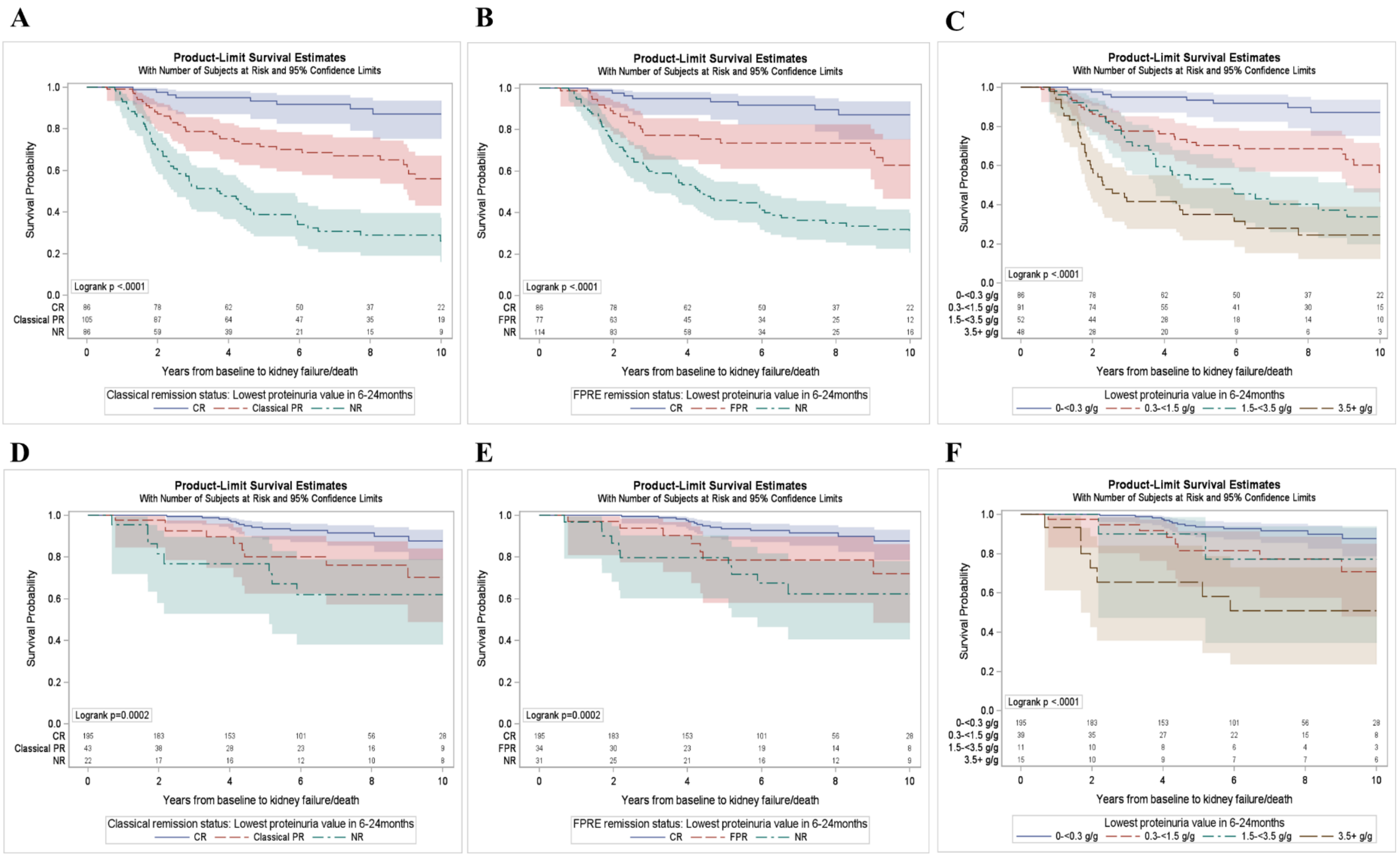

Notes: FSGS, Focal Segmental Glomerulosclerosis; MCD, minimal change disease; CR, complete remission; FPR, FSGS partial remission; FSGS, focal segmental glomerulosclerosis; NR, no remission; PR, partial remission.

Supplemental Figure 5. Kaplan-Meier survival curves for prevalent biopsy populations: (A) FSGS classical remissions, (B) FSGS partial remission, (C) FSGS threshold approach, (D) MCD classical remissions, (E) MCD partial remission, and (F) MCD threshold approaches. All analyses were based on the lowest proteinuria value recorded within 6-12 months post-baseline.

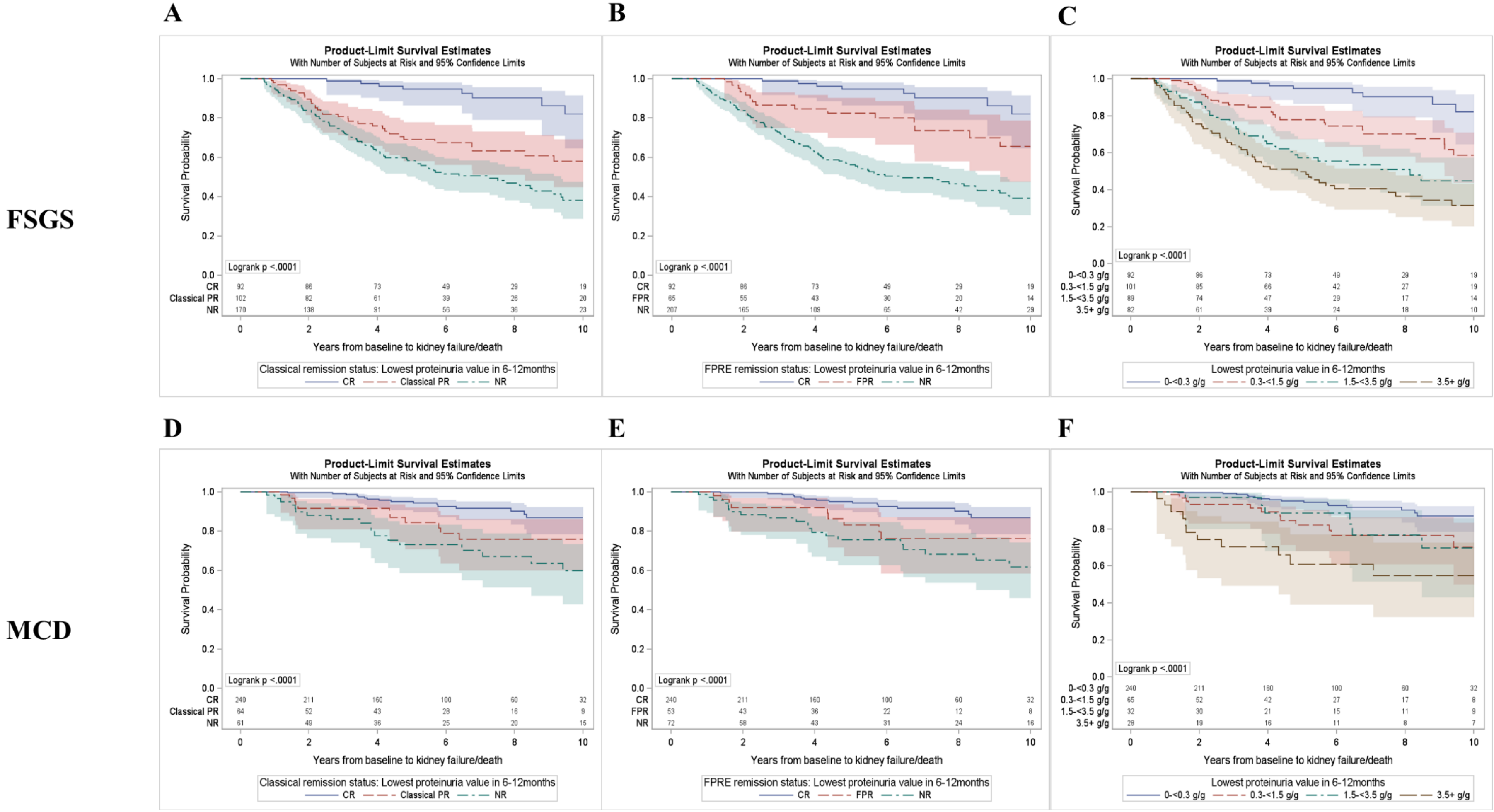

Notes: FSGS, Focal Segmental Glomerulosclerosis; MCD, minimal change disease; CR, complete remission; FPR, FSGS partial remission; FSGS, focal segmental glomerulosclerosis; NR, no remission; PR, partial remission.

Supplemental Figure 6. Kaplan-Meier survival curves for incident biopsy populations: (A) FSGS classical remissions, (B) FSGS partial remission, (C) FSGS threshold approach, (D) MCD classical remissions, (E) MCD partial remission, and (F) MCD threshold approaches. All analyses were based on the time-averaged proteinuria within 6-12 months post-baseline.

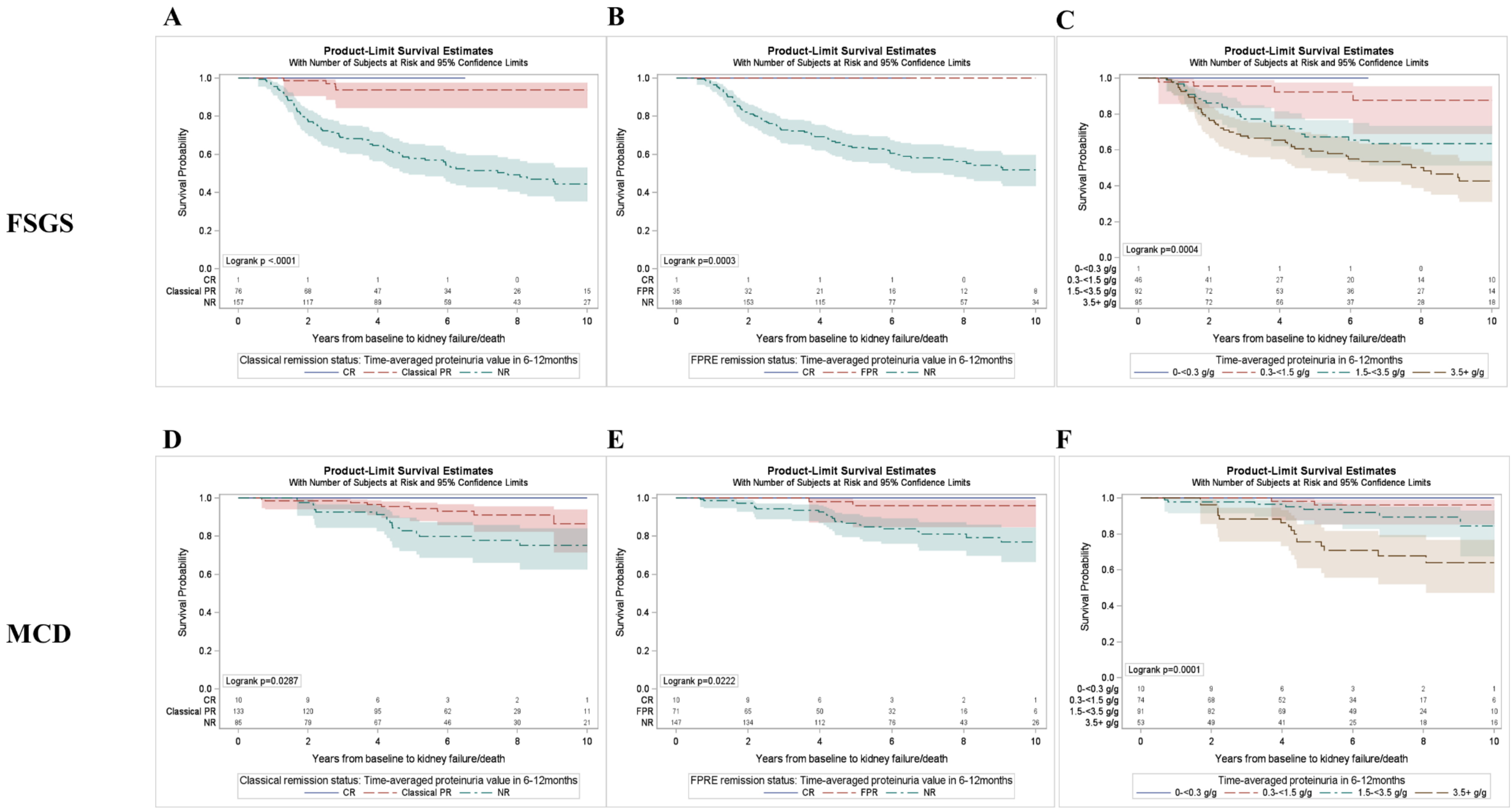

Notes: FSGS, Focal Segmental Glomerulosclerosis; MCD, minimal change disease; CR, complete remission; FPR, FSGS partial remission; FSGS, focal segmental glomerulosclerosis; NR, no remission; PR, partial remission.

Supplemental Figure 7. Kaplan-Meier survival curves for prevalent biopsy populations: (A) FSGS classical remissions, (B) FSGS partial remission, (C) FSGS threshold approach, (D) MCD classical remissions, (E) MCD partial remission, and (F) MCD threshold approaches. All analyses were based on the time-averaged proteinuria within 6-24 months post-baseline.

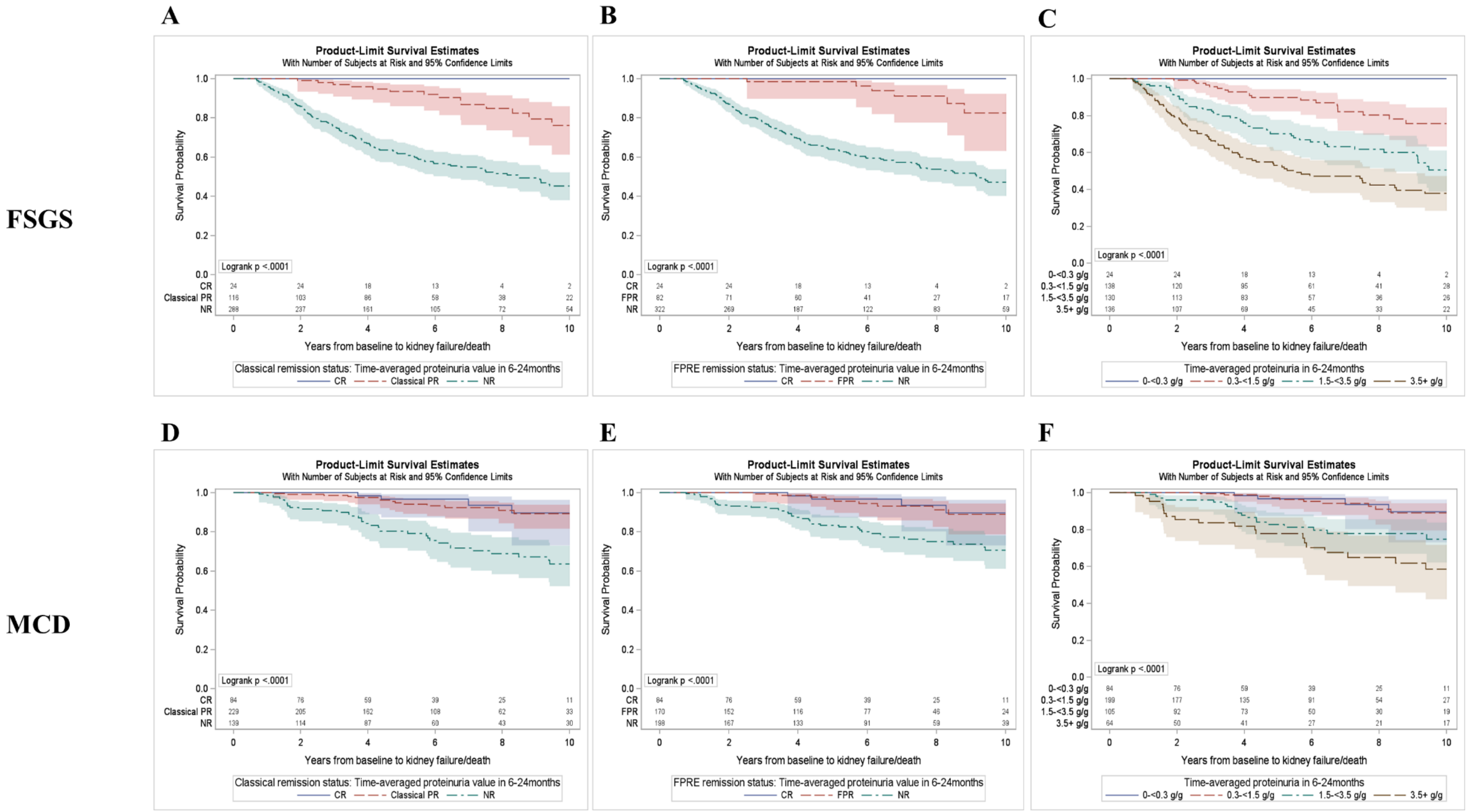

Notes: FSGS, Focal Segmental Glomerulosclerosis; MCD, minimal change disease; CR, complete remission; FPR, FSGS partial remission; FSGS, focal segmental glomerulosclerosis; NR, no remission; PR, partial remission.

Supplemental Figure 8. Forest plots of prevalent FSGS-biopsy proteinuria analysis population. Percentage change from baseline for lowest proteinuria value (LPV) within 6-12 months post-baseline vs (A) Hazard Ratio for kidney failure/death event and vs (B) eGFR slope over 6 months to 10 years. Percentage change from baseline for time-averaged proteinuria (TAP) within 6-24 months post-baseline vs (C) Hazard Ratio for kidney failure/death event and vs (D) eGFR slope over 6 months to 10 years.

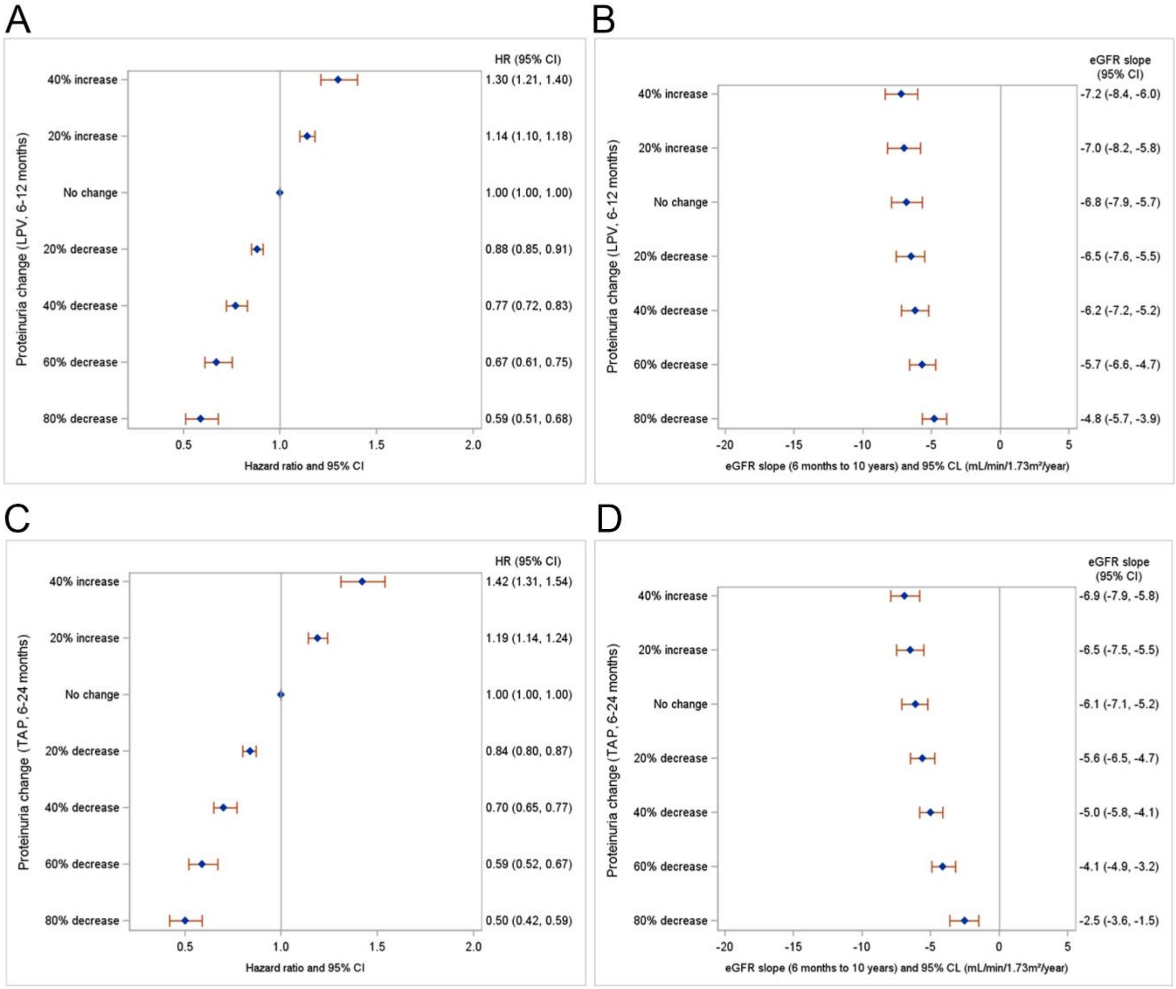

Notes: eGFR, estimated glomerular filtration rate; FSGS, Focal Segmental Glomerulosclerosis; TAP, time-averaged proteinuria, LPV, lowest proteinuria value.

Supplemental Figure 9. Forest plots of incident MCD-biopsy proteinuria analysis population. Percentage change from baseline for lowest proteinuria value (LPV) within 6-12 months post-baseline vs (A) Hazard Ratio for kidney failure/death event and vs (B) eGFR slope over 6 months to 10 years. Percentage change from baseline for time-averaged proteinuria (TAP) within 6-24 months post-baseline vs (C) Hazard Ratio for kidney failure/death event and vs (D) eGFR slope over 6 months to 10 years.

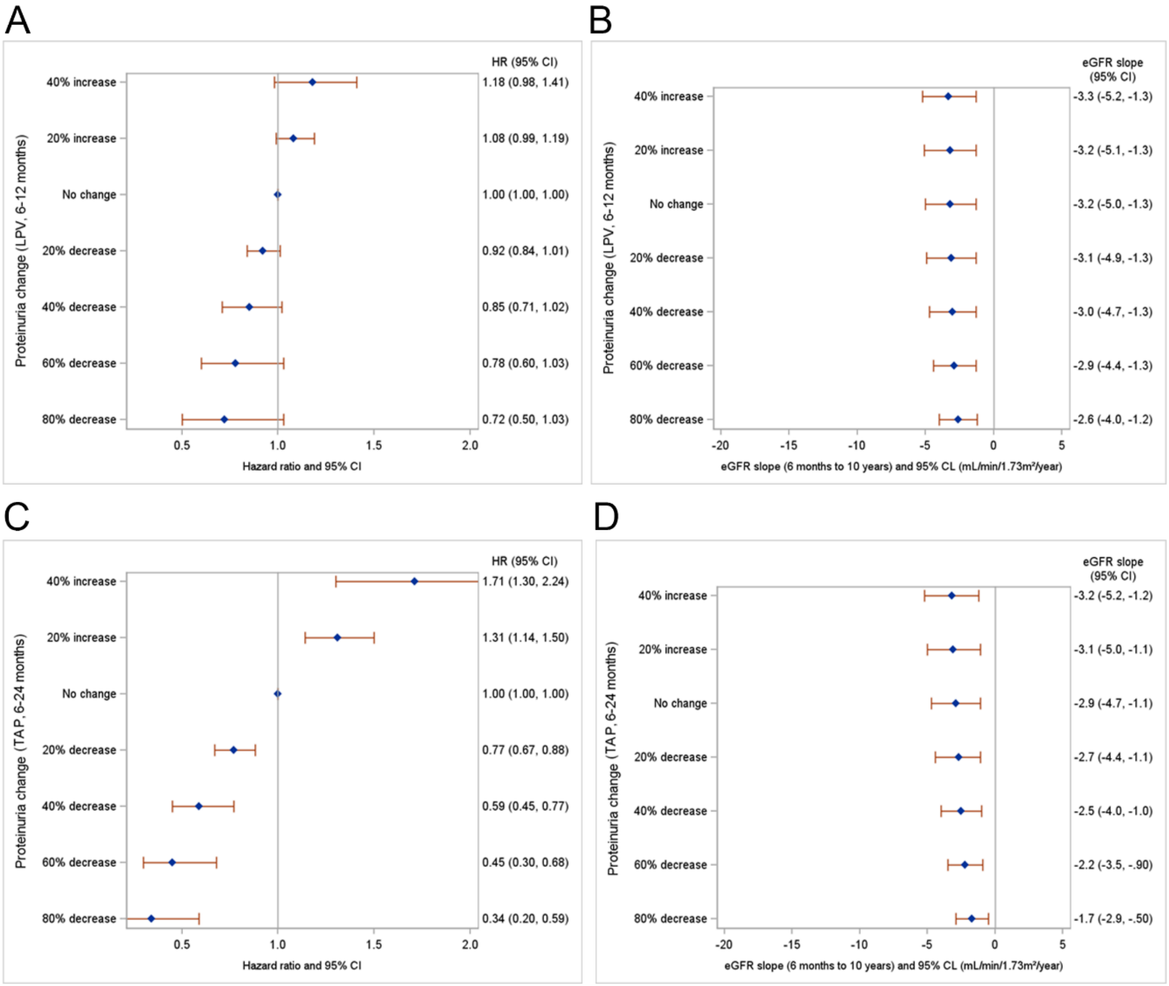

Notes: eGFR, estimated glomerular filtration rate; MCD, minimal change disease; TAP, time-averaged proteinuria, LPV, lowest proteinuria value.

Supplemental Figure 10. Forest plots of prevalent MCD-biopsy proteinuria analysis population. Percentage change from baseline for lowest proteinuria value (LPV) within 6-12 months post-baseline vs (A) Hazard Ratio for kidney failure/death event and vs (B) eGFR slope over 6 months to 10 years. Percentage change from baseline for time-averaged proteinuria (TAP) within 6-24 months post-baseline vs (C) Hazard Ratio for kidney failure/death event and vs (D) eGFR slope over 6 months to 10 years.

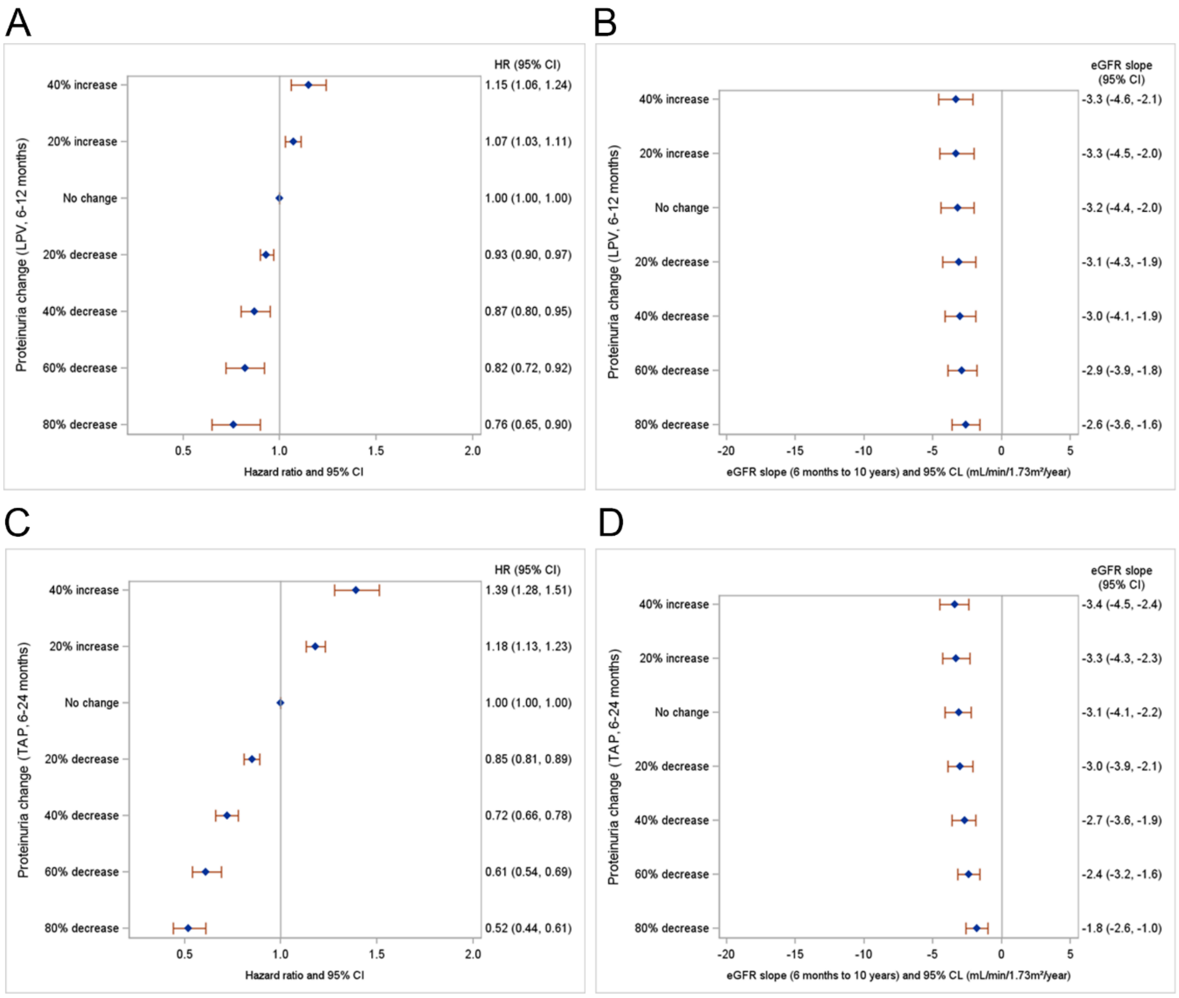

Notes: eGFR, estimated glomerular filtration rate; MCD, minimal change disease; TAP, time-averaged proteinuria, LPV, lowest proteinuria value.

Supplemental Figure 11. Kaplan-Meier survival curves of incident FSGS-biopsy proteinuria analysis population: Proteinuria response category approach applying (A) lowest proteinuria value within 6-24 months post-baseline and (B) time-averaged proteinuria within 6-12 months post-baseline

A: Lowest proteinuria value 6-24 months

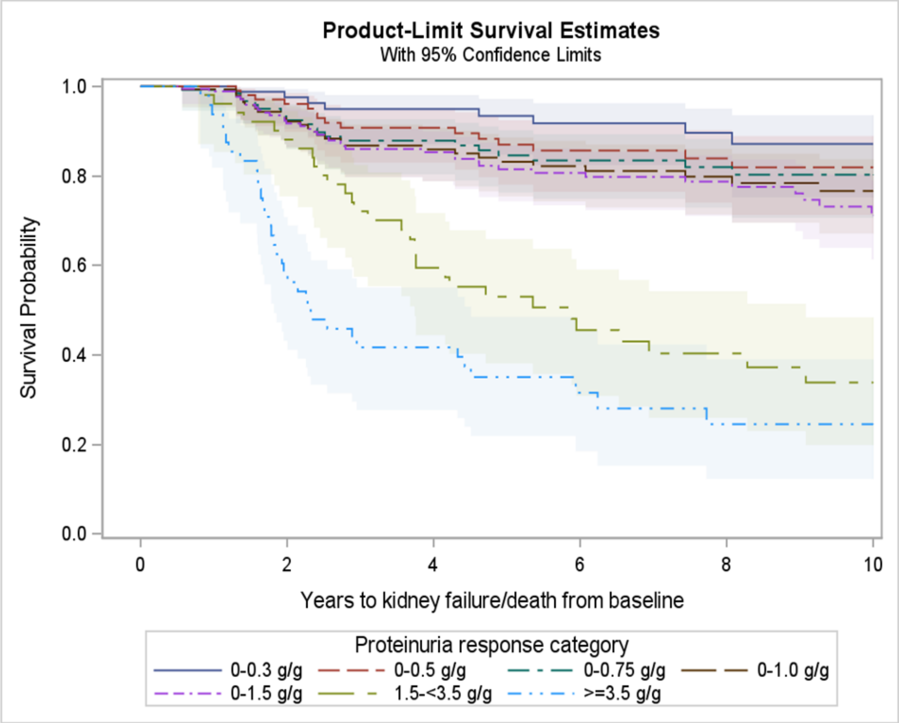

B: Time-averaged proteinuria 6-12 months

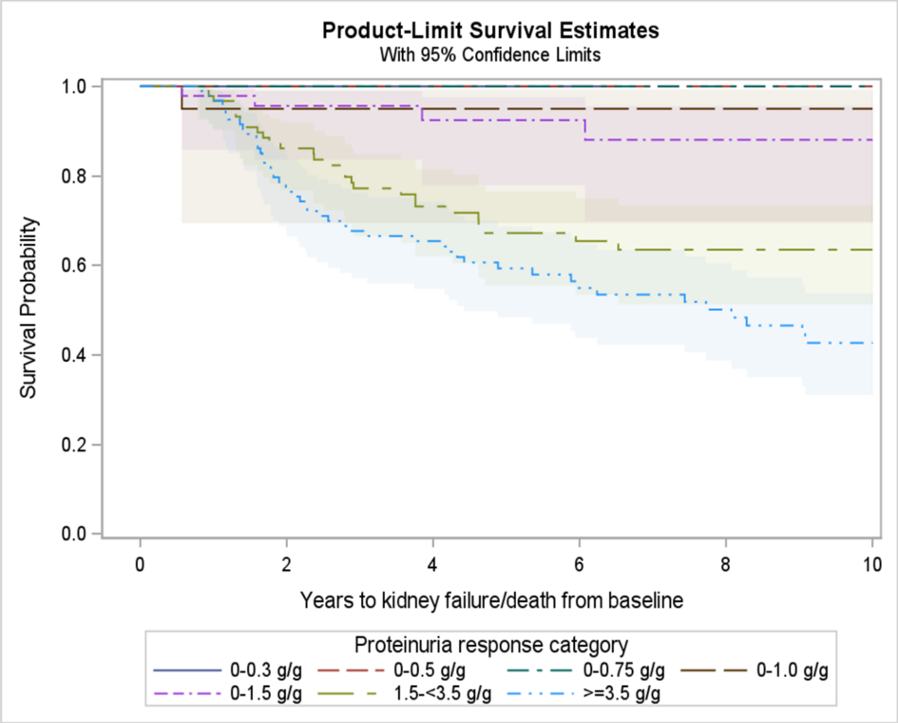

Notes: FSGS, Focal Segmental Glomerulosclerosis.

Supplemental Figure 12. Kaplan-Meier survival curves of prevalent FSGS-biopsy proteinuria analysis population: Proteinuria response category approach applying (A) lowest proteinuria value within 6-12 months post-baseline and (B) time-averaged proteinuria within 6-24 months post-baseline

A: Lowest proteinuria value 6-12 months

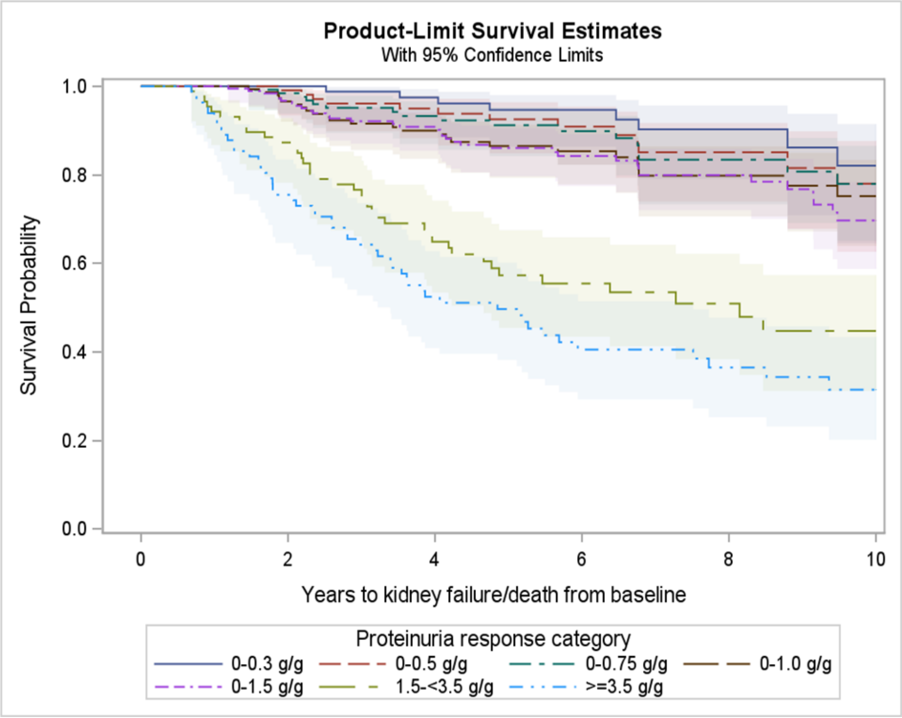

B: Time-averaged proteinuria 6-24 months

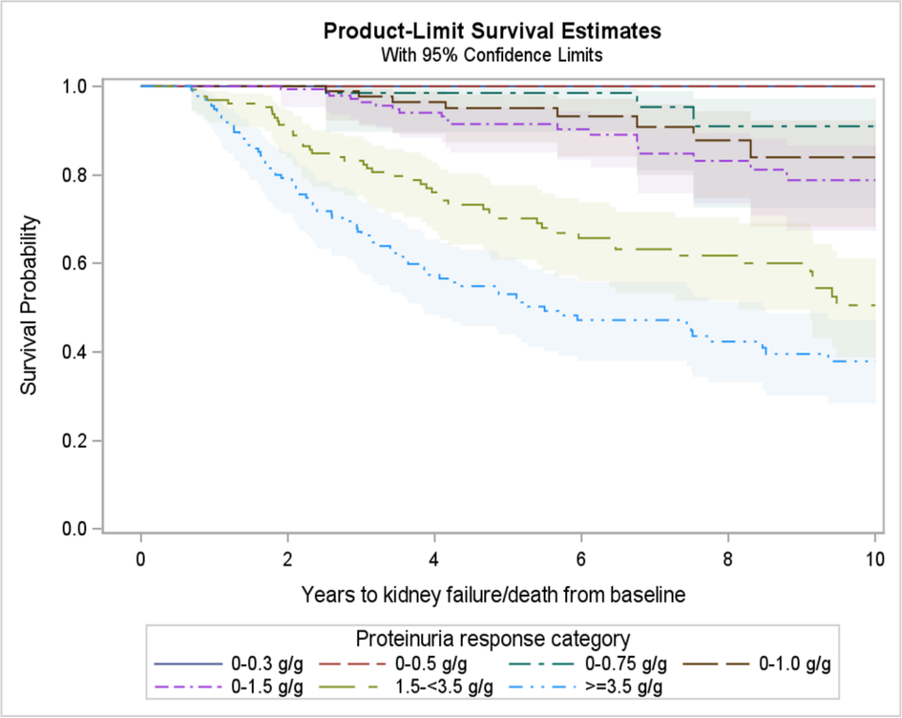

Notes: FSGS, Focal Segmental Glomerulosclerosis.

Supplemental Figure 13. Number of patients contributing data in each calendar year.

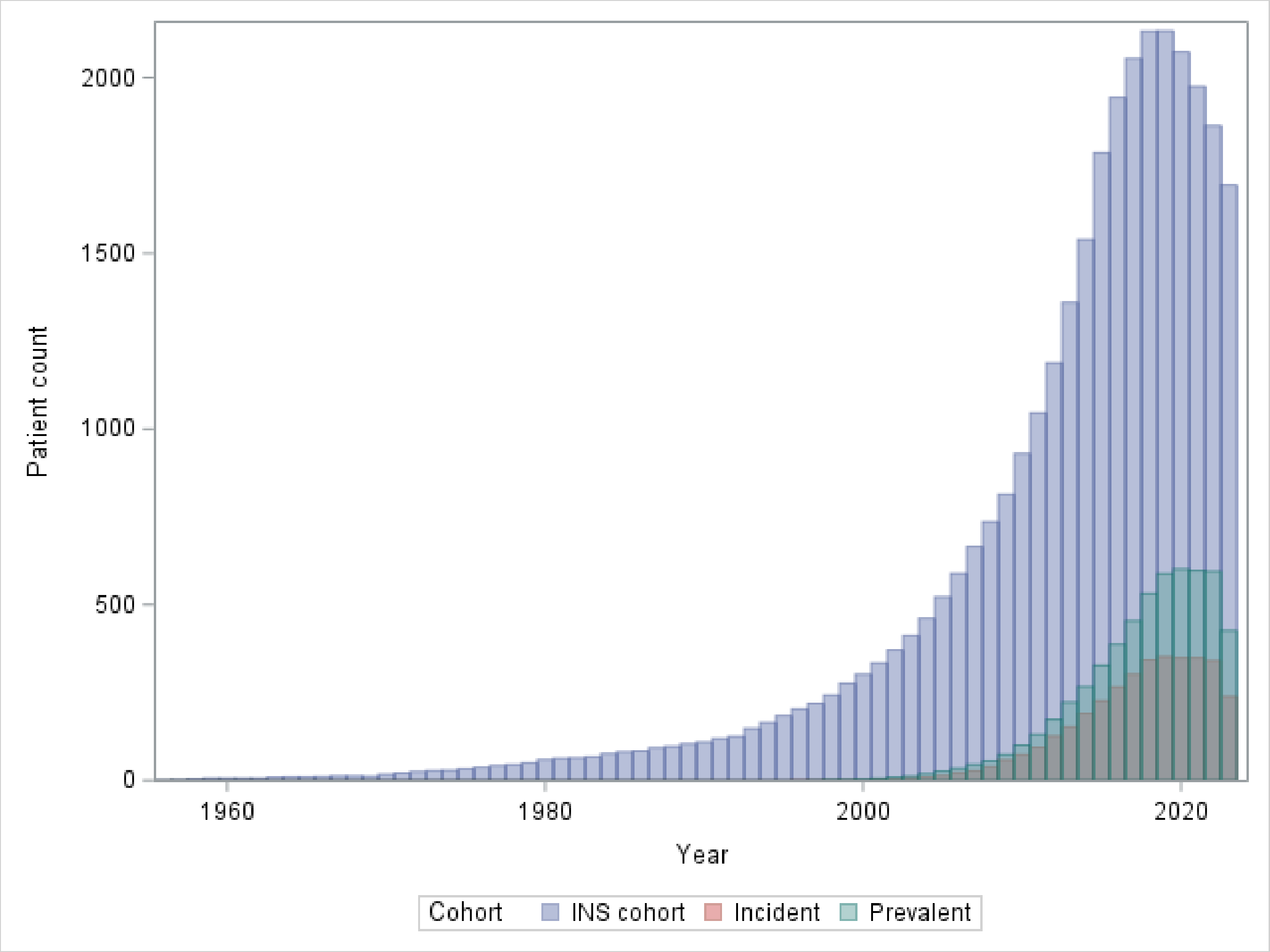

;

Notes: INS, idiopathic nephrotic syndrome patients; MCD, minimal change disease; UPCR, urine protein:creatinine ratio.

Supplemental Figure 14. Association between the number of UPCR measurements used in the calculation of proteinuria values and outcomes.

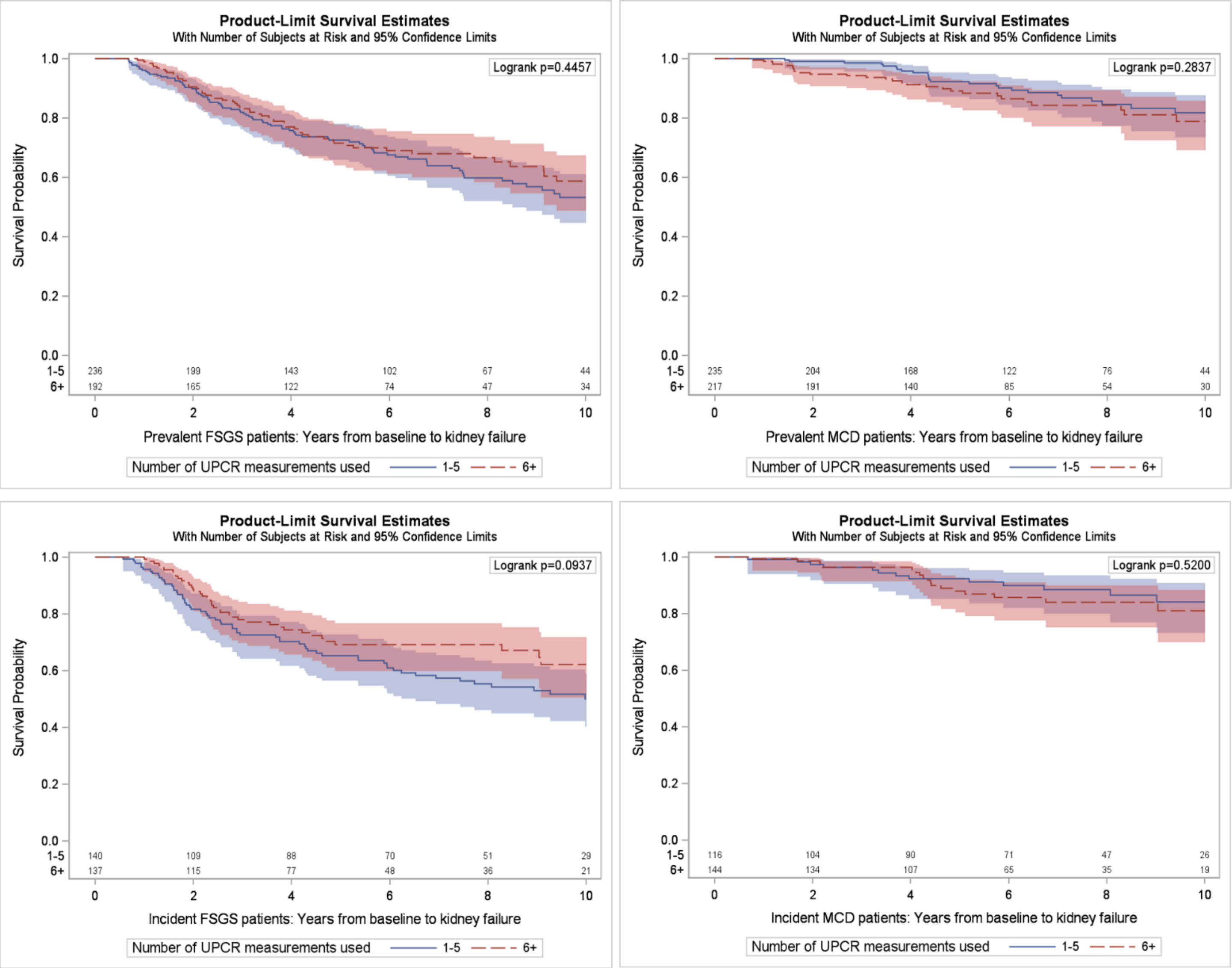

Notes: FSGS, Focal Segmental Glomerulosclerosis; MCD, minimal change disease; UPCR, urine protein:creatinine ratio.

References

1. Wong K, Pitcher D, Braddon F, et al. Description and Cross-Sectional Analyses of 25,880 Adults and Children in the UK National Registry of Rare Kidney Diseases Cohort. *Kidney International Reports*. doi:10.1016/j.ekir.2024.04.062

2. Le W, Liang S, Hu Y, et al. Long-term renal survival and related risk factors in patients with IgA nephropathy: results from a cohort of 1155 cases in a Chinese adult population. *Nephrol Dial Transplant*. Apr 2012;27(4):1479-1485. doi:10.1093/ndt/gfr527

3. Levey AS, Stevens LA, Schmid CH, et al. A new equation to estimate glomerular filtration rate. *Ann Intern Med*. May 5 2009;150(9):604-612. doi:10.7326/0003-4819-150-9-200905050-00006

4. Schwartz GJ, Muñoz A, Schneider MF, et al. New equations to estimate GFR in children with CKD. *J Am Soc Nephrol*. Mar 2009;20(3):629-637. doi:10.1681/asn.2008030287

5. Atkins RC, Briganti EM, Zimmet PZ, Chadban SJ. Association between albuminuria and proteinuria in the general population: the AusDiab Study. *Nephrol Dial Transplant*. Oct 2003;18(10):2170-2174. doi:10.1093/ndt/gfg314

6. Troost JP, Trachtman H, Spino C, et al. Proteinuria Reduction and Kidney Survival in Focal Segmental Glomerulosclerosis. *Am J Kidney Dis*. Feb 2021;77(2):216-225. doi:10.1053/j.ajkd.2020.04.014
